# Cardiovascular events and all‐cause mortality in patients with chronic obstructive pulmonary disease using olodaterol and other long‐acting beta2‐agonists

**DOI:** 10.1002/pds.5432

**Published:** 2022-05-13

**Authors:** Cristina Rebordosa, Dóra Körmendiné Farkas, Jukka Montonen, Kristina Laugesen, Florian Voss, Jaume Aguado, Ulrich Bothner, Kenneth J. Rothman, Kristina Zint, Daniel Mines, Vera Ehrenstein

**Affiliations:** ^1^ RTI Health Solutions Barcelona Spain; ^2^ Aarhus University Aarhus Denmark; ^3^ Boehringer Ingelheim International GmbH Ingelheim am Rhein Germany; ^4^ RTI Health Solutions Waltham Massachusetts USA; ^5^ RTI Health Solutions Philadelphia Pennsylvania USA

**Keywords:** adrenergic beta‐2 receptor agonists, cardiac arrhythmias, channelling, Denmark, myocardial ischaemia, olodaterol, routinely collected health data

## Abstract

**Purpose:**

We examined the effect of olodaterol on the risk of myocardial ischaemia, cardiac arrhythmia, and all‐cause mortality compared with use of other long‐acting beta2‐agonists (LABAs). Channelling bias was also explored.

**Methods:**

This Danish population‐based cohort study used data linked from registries of hospital diagnoses, outpatient dispensings, and deaths. It included patients (aged ≥40 years) with a diagnosis of chronic obstructive pulmonary disease (COPD) who initiated olodaterol or another LABA. Using matching and propensity score (PS) stratification, we calculated adjusted incidence rate ratios (IRRs) using Poisson regression, followed by several additional analyses to evaluate and control channelling bias.

**Results:**

The IRRs of cardiac arrhythmias or myocardial ischaemia among users of olodaterol (*n* = 14 239) compared to users of other LABAs (*n* = 51 167) ranged from 0.96 to 1.65 in various analyses, although some estimates had low precision. Initial analysis suggested an increased risk for death with olodaterol compared with other LABAs (IRR, 1.63; 95% CI, 1.44–1.84). Because olodaterol prescribing was associated with COPD severity, the mortality association was attenuated by using different methods of tighter confounding control: the IRRs were 1.26 (95% CI, 0.97–1.64) among LABA‐naïve LABA/LAMA users without recent COPD hospitalisation; 1.27 (95% CI, 1.03–1.57) in a population with additional trimming from the tails of the PS distribution; and 1.32 (95% CI, 1.19–1.48) after applying overlap‐weights analysis.

**Conclusions:**

Olodaterol users had a similar risk for cardiac arrhythmias or myocardial ischaemia as other LABA users. The observed excess all‐cause mortality associated with olodaterol use could be due to uncontrolled channelling bias.

**Key Points**

- This post‐authorisation safety study examined the risks of myocardial ischaemia, cardiac arrhythmia, and all‐cause mortality among olodaterol users versus users of other long‐acting beta2‐agonists (LABAs).
- Olodaterol users were at similar risk for myocardial ischaemia or cardiac arrhythmia as other LABAs.
- Olodaterol was preferentially prescribed to patients with more severe chronic obstructive pulmonary disease, suggesting the presence of a channelling bias.
- The observed increased risk for all‐cause mortality among olodaterol users was attenuated in response to tighter confounding control.

**Plain Language Summary**

Olodaterol and other long‐acting beta2‐agonist (LABA) drugs are used to treat chronic obstructive pulmonary disease (COPD). In a Danish population study, we examined risks of myocardial ischaemia, cardiac arrhythmia, and mortality by calculating incidence rate ratios (IRRs) for olodaterol use compared with other LABAs. We used hospital diagnosis and outpatient prescription data to identify patients aged ≥40 years with COPD who started olodaterol or another LABA. IRRs of cardiac arrhythmias or myocardial ischaemia among users of olodaterol (n=14 239) versus other LABAs (n=51 167) ranged from 0.96 to 1.65 and 95% confidence intervals (CIs) for some outcomes were wide. Initial analysis suggested increased mortality risk with olodaterol versus other LABAs (IRR, 1.63; 95% CI, 1.44‐1.84). However, olodaterol prescribing was associated with COPD severity; increased mortality risk lessened to 1.26 (95% CI, 0.97‐1.64) when we accounted for preferential prescribing of olodaterol to patients with more severe COPD and other potential differences in baseline characteristics. In conclusion, olodaterol users had similar risks of cardiac arrhythmias and myocardial ischaemia as other LABA users, and the observed increase in mortality with olodaterol use may be due to prescribing of olodaterol to patients with more severe COPD.

## 1 INTRODUCTION

Inhaled long‐acting beta2‐agonist (LABA) drugs are used as maintenance treatment in chronic obstructive pulmonary disease (COPD) to relieve bronchial constriction and associated symptoms. Olodaterol is a LABA used as a monotherapy (Striverdi®; Boehringer Ingelheim, Ingelheim am Rhein, Germany) or as a fixed‐dose combination (FDC) with the long‐acting muscarinic antagonist (LAMA) tiotropium (Spiolto®; Boehringer Ingelheim). As a possible side effect outside the lungs, nonselective stimulation of beta1‐adrenoreceptors by LABAs in the heart may lead to positive inotropic and chronotropic responses, increased heart rate and myocardial oxygen demand as well as myocardial ischaemia and arrhythmia, particularly with pre‐existing coronary artery disease. Stimulation of vascular beta2‐adrenoreceptors can also lead to peripheral vasodilation and reflex tachycardia. Finally, LABAs may also lead to hypokalemia, and consequent ventricular tachycardia or fibrillation might occur through activation of beta2‐adrenoreceptors in the skeletal muscles.^1^, ^2^ Olodaterol clinical trials did not detect increased frequency of any cardiovascular events, including myocardial ischaemia (i.e., ischaemic heart disease), cardiac arrhythmias, or a persistent increase in heart rate.^3^, ^4^, ^5^, ^6^ Nevertheless, cardiac arrhythmia and myocardial ischaemia are acknowledged pharmacological class effects of LABAs which were also mentioned in the newly approved product information for Striverdi Respimat®. At the time of the approval in 2013, the health authorities of the European Union requested a post‐authorisation safety study (PASS) to evaluate the long‐term safety of olodaterol. In response to this request, we conducted a PASS using data from the Danish population‐based registries to evaluate the extent to which the use of olodaterol alone or olodaterol in FDC with tiotropium is associated with an increased risk of myocardial ischaemia, cardiac arrhythmia, or all‐cause mortality compared with the use of other LABAs. Channelling is a type of selection bias that occurs when prescribing is related to prognosis, despite treatment indications similar to that of alternatives. Here, we report the results of this PASS and discuss the methodological challenges, including the potential role of channelling bias on the results.

## 2 METHODS

### 2.1 Study design, data source, and study population

This cohort study was conducted in Denmark using data from nationwide, population‐based registries. The Danish health care system provides tax‐supported universal health care to all residents.^7^, ^8^ Relevant inpatient and outpatient hospital diagnoses were identified in the Danish National Patient Registry,^9^ and outpatient dispensings were identified in the Danish National Prescription Registry.^10^ Information on all‐cause mortality and emigration originated from the Danish Civil Registration System. Patient‐level data across registries were linked using unique, government‐issued identity numbers assigned to all Danish residents at birth or immigration.^7^

This study examined patients aged ≥40 years with a diagnosis of COPD who initiated treatment with olodaterol (either alone or in free‐dose combination or FDC with a LAMA) or with an alternative LABA (either alone or in free‐dose combination or FDC with a LAMA) (Figure S1). Patients included in the study were new users of olodaterol or new users of another LABA (i.e., formoterol, salmeterol, indacaterol, or vilanterol) from 1 March 2014 through 31 January 2019. The date of first dispensing during the study period that fulfilled all eligibility criteria was the index date. Eligible patients also met the following criteria: (1) they had been dispensed a study LABA medication during the study period with no dispensings of the same medication during the 180 days before the index date, (2) they had at least 1 year of uninterrupted residence in Denmark before the index date, (3) they were 40 years or older at the index date, and (4) they had a recorded hospital diagnosis of COPD, with or without asthma, at any time before the index date.

LABA exposure was assessed from dispensings, and patients were assigned to the olodaterol cohort or the comparator cohort. For each patient, duration of use (or days' supply) was estimated based on the number of defined daily doses. For the exposure period, we considered only the first episode of continuous use, defined as the period that started on the dispensing date and ended 14 days after the end of the days' supply (carryover period), allowing for gaps of no drug supply of up to 14 days between sequential dispensings. Follow‐up started at the index date and ended at the first of these events: end of the first episode of continuous use, addition of a second LABA, earliest occurrence of an outcome of interest, death, emigration, or end of the study period. Diagnoses of COPD were identified through inpatient primary hospital discharge diagnoses or outpatient hospital specialist clinic diagnoses recorded in the Danish National Patient Registry^9^ (*International Classification of Diseases, Tenth Revision* [ICD‐10] codes; see Table S1).^11^

Patients in the comparator cohort were individually matched, with replacement, to patients in the olodaterol cohort in a ratio up to 4:1 by age, sex, and calendar year. Within this study population, we used multivariable logistic regression to estimate a propensity score (PS) for each patient, as the conditional probability of receiving olodaterol instead of another LABA, given a set of observed covariables. Covariates were selected as potential risk factors for cardiac arrhythmias and myocardial ischaemia, the primary outcomes of the study. Use of LAMA in free‐dose combination or FDC with the study medications at index date was not included due to its strong association with exposure, leading to over‐separation of the PS distribution curves.^12^ LAMA use at the index date was included as a covariate in the Poisson regression model. Patients with a PS below the 2.5 percentile of the olodaterol‐exposed distribution of scores and all those with a PS greater than the 97.5 percentile of the “other LABA” distribution of scores were trimmed (i.e., excluded) (Figure S2). Patients with broken matches after trimming were also excluded, i.e., if patients in the olodaterol cohort were left without any match to another LABA patient, they were excluded, and if patients in the other LABA cohort were left with no olodaterol match, they were also excluded.

### 2.2 Study outcomes and covariables

The study primary outcomes were cardiac arrhythmias and myocardial ischaemia, which were defined using validated algorithms.^11^, ^13^, ^14^, ^15^, ^16^, ^17^, ^18^ Cardiac arrhythmias were assessed through primary hospital discharge diagnoses or outpatient specialist clinic visits for atrial fibrillation or flutter (AF) and supraventricular tachycardia (SVT) as well as through primary hospital discharge diagnoses for ventricular tachycardia (VT), including ventricular fibrillation/flutter and cardiac arrest. Myocardial ischaemia was assessed by examining primary hospital discharge diagnosis of acute myocardial infarction (AMI) or of serious acute coronary heart disease (SACHD), including angina and other acute ischaemic heart disease events. No outpatient data were used to identify VT, AMI, and SACHD because these serious conditions require in‐hospital admission.^15^ All‐cause mortality (a secondary outcome) was ascertained from the Danish Civil Registration System, which is a reliable source of death information.^8^ History of comorbidities was ascertained as of the index date for most conditions (Figure S1). Severity of COPD was categorised based on an algorithm proposed by Verhamme et al. (Table S6).^19^ Prior use of medications was ascertained within 180 days before the index date, except concomitant use of inhaled corticosteroid and LAMAs, which included 30 days before and after the index date (Figure S1). See codes for diagnoses and medications in Tables S1–S5.

### 2.3 Statistical analyses

For each outcome, we calculated crude and adjusted incidence rate ratios (IRRs), incidence rate differences (IRDs), and their 95% confidence intervals (CIs) for the overall study population, using Poisson regression with robust estimation of the variance to account for repeated observations by some patients.^20^ We used matching by age, sex, and calendar year and adjustment for PS strata, which were included in the Poisson model as an indicator variable, to control measured confounding. Patients from each cohort were categorised into five mutually exclusive strata defined by PS quintiles using the distribution of the olodaterol‐exposed population.^21^ Balance, measured as the standardised bias (SB) or difference in proportions of each level of the covariable for the “other LABA” cohort minus the olodaterol cohort divided by the standard deviation of the covariable in the “other LABA” cohort, was considered satisfactory if the overall SB was no greater than 0.2 (see definitions in the Online Supporting Information).

Initial results indicated channelling of olodaterol prescription to patients with more severe COPD. To evaluate the observed differences in the study cohorts and the effect of uncontrolled confounding in the study results, we performed additional analyses (see Supporting Information). The original analysis focused on risk factors for the primary outcomes, and only a proxy for COPD severity was included. However, several factors that are known predictors of mortality were originally not included in the PS and showed imbalances between treatment groups within the PS strata. These factors were prior hospitalisations for any cause, hospitalisations for COPD, and COPD exacerbations within 90 or 180 days before the index date. In the additional analyses, we adjusted for prognostic variables that had not been included in the original analyses. In these analyses, restriction was used to balance cohort characteristics more closely at baseline. We also performed an analysis using PSs with overlap weights to upweight those with the most overlap in observed characteristics between treatments, i.e., the population with greatest treatment equipoise.^22^ In addition, we performed a series of analyses to assess the robustness of results with respect to the analysis techniques used to evaluate all‐cause mortality (Tables S7 and S8). To improve balance in the distribution of the baseline characteristics in additional analyses, we used a threshold of 0.1 for an average SB.

We also used bias analysis to evaluate the potential effects of confounding from unmeasured factors. Factors not available included body mass index (BMI), smoking, dyspnoea, and measures of airway obstruction. We obtained estimates of the effects of these factors from the results of the TIOtropium Safety and Performance In Respimat (TIOSPIR) trial.^23^ These estimates were shown to be independent risk factors for mortality after adjusting for other important risk factors. We evaluated the potential effect of each variable measured at cohort entry. Each assessment evaluated the effect of the potential unmeasured confounder across a range of empirically observed prevalence differences in the study data.

## 3 RESULTS

### 3.1 Study population

**FIGURE 1:**
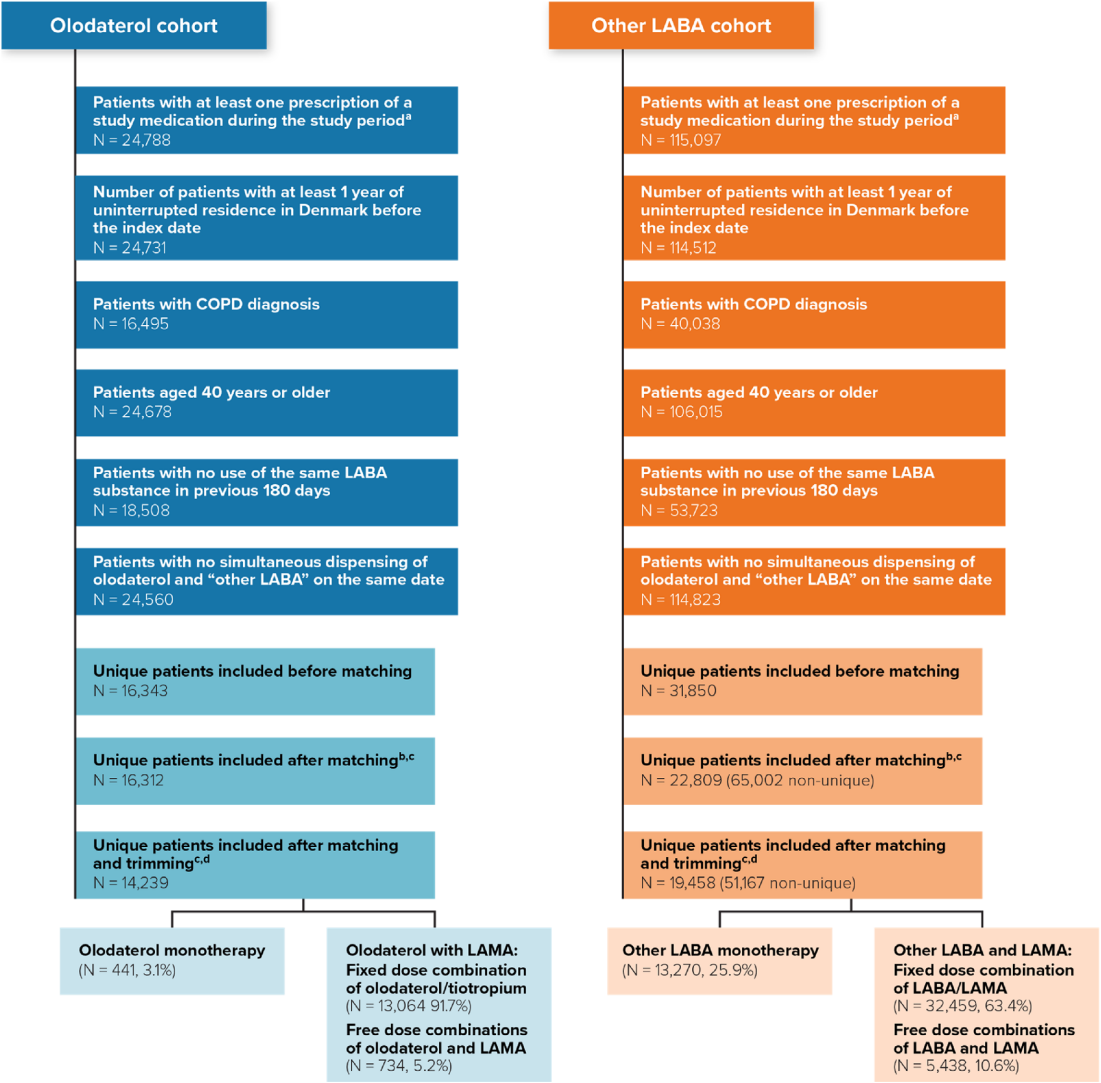
Cohort attrition. COPD, chronic obstructive pulmonary disease; LABA, inhaled long‐acting beta2‐agonists; LAMA, long‐acting muscarinic antagonist. The different inclusion criteria were applied independently and are not mutually exclusive. Percentages in this table represent the percentage among the patients who were potentially eligible for the study as noted in the first row (i.e., those with at least one prescription of a study medication during the study period). The other LABA cohort comprised inhaled long‐acting beta2‐agonists other than olodaterol. ^a^Patients of all ages with at least one prescription of olodaterol or LABA other than olodaterol recorded in the Danish National Prescription Registry from March 2014 through January 2019. ^b^Matching ratio up to 1:4, olodaterol patients to “other LABA” patients. ^c^Among those patients who were excluded because of broken matches after trimming, 6239 patients were unique. However, it is still possible that these patients were matched to other olodaterol patients who were not excluded. ^d^A total of <5 users of olodaterol and 908 users of other LABA were excluded by broken matches

The study population after matching and trimming comprised 14 239 patients who used olodaterol (3.1% monotherapy) and 51 167 patients who used another LABA (25.9% monotherapy) (Figure 1). The median (quarter 1, quarter 3) follow‐up time was 2.7 (1.5, 5. 5) months for the olodaterol cohort and 2.4 (1.5, 5.4) months for the other LABA cohort. Duration of follow‐up was more than 6 months for 23% of the patients in the olodaterol cohort and for 22% of the patients in the other LABA cohort. The most frequent reason (61.2%–63.7%) for ending follow‐up was discontinuation of the first episode of continuous use (Table S9). The description of baseline characteristics after trimming was consistent with preferential prescription (channelling) of olodaterol to patients with more severe COPD (Table 1). Additional analysis showed imbalances between olodaterol and other LABA cohorts within PS strata with respect to important risk factors for mortality, such as the number of hospitalisations with COPD and exacerbations before the index date (Table S10). These analyses also showed an increasing COPD severity with increasing PS strata (Table S11).

**TABLE 1:** Patient demographics, clinical characteristics, and medications at the index date in each study cohort after trimming and matching

	Olodaterol		Other LABA	
	*N* = 14 239		*N* = 51 167	
Age (years)				
Mean (SD)	72.7	10	72.7	10
Median (Q1, Q3)	73	66–80	73	66–80
Min, max	40	100	40	100
Age group (years), %				
40–60	1700	11.9	6273	12.3
61–74	6089	42.8	21 817	42.6
75–84	4819	33.8	17 166	33.5
85 or more	1631	11.5	5911	11.6
Female, %	7649	53.7	27 253	53.3
Calendar year at index, %				
2014	390	2.7	1320	2.6
2015	1407	9.9	4829	9.4
2016	3344	23.5	11 847	23.2
2017	4561	32.0	16 424	32.1
2018	4197	29.5	15 573	30.4
2019	340	2.4	1174	2.3
Cardiovascular diseases, %	10 500	73.7	37 435	73.2
Ischaemic heart disease	4138	29.1	14 652	28.6
Angina pectoris	3072	21.6	10 968	21.4
Acute myocardial infarction	1479	10.4	5278	10.3
Other acute or subacute ischaemic heart disease	170	1.2	619	1.2
Chronic ischaemic heart disease	2661	18.7	9401	18.4
Coronary reperfusion surgery and procedures	1558	10.9	5671	11.1
Conduction disorders	473	3.3	1641	3.2
Cardiac arrest	92	0.6	261	0.5
Arrhythmias (hospitalisation for)	3285	23.1	11 295	22.1
Paroxysmal tachycardia	673	4.7	2238	4.4
Ventricular tachycardia	182	1.3	535	1.0
Supraventricular tachycardia and unspecified	437	3.1	1494	2.9
Atrial fibrillation or flutter	2740	19.2	9373	18.3
Other cardiac arrhythmias	773	5.4	2705	5.3
Ventricular fibrillation and flutter	56	0.4	192	0.4
Other cardiac arrhythmias	727	5.1	2554	5.0
Heart failure	2236	15.7	7469	14.6
Cerebrovascular disease	2456	17.2	8535	16.7
Cerebral haemorrhage	234	1.6	724	1.4
Cerebral infarction and stroke	1543	10.8	5277	10.3
Transient ischaemic attack	815	5.7	2941	5.7
Other cerebrovascular disease and sequelae of cerebrovascular disease	1277	9.0	4283	8.4
Hypertension and hypertensive heart disease	5204	36.5	19 362	37.8
Diseases of arteries, arterioles, and capillaries	2779	19.5	9767	19.1
Peripheral arterial revascularisation procedures	1154	8.1	3946	7.7
Other form of heart diseases	3988	28.0	14 014	27.4
Hyperlipidaemia	2591	18.2	9470	18.5
Diabetes mellitus	2532	17.8	9195	18.0
Renal disease	4139	29.1	14 081	27.5
Chronic kidney disease	684	4.8	2339	4.6
Other renal disorders	3640	25.6	12 268	24.0
Anaemias	1619	11.4	5411	10.6
Nutritional anaemias	659	4.6	2018	3.9
Iron deficiency anaemias	563	4.0	1679	3.3
Other anaemias	1324	9.3	4510	8.8
Peptic ulcer disease	1313	9.2	4467	8.7
Liver disease	562	3.9	1962	3.8
Osteoporosis	3374	23.7	10 484	20.5
Rheumatoid arthritis and other inflammatory arthropathies	1369	9.6	4706	9.2
Systemic connective tissue diseases	627	4.4	2348	4.6
Malignancy	3471	24.4	12 122	23.7
Depressive disorders	805	5.7	2835	5.5
Asthma	2426	17.0	8682	17.0
Pregnancy (at the index date)	0	0	0	0
Respiratory medications	13 109	92.1	43 585	85.2
SAMAs	48	0.3	183	0.4
LAMAs	7219	50.7	20 115	39.3
SABAs	8591	60.3	26 365	51.5
LABAs	1346	9.5	2774	5.4
ICS	2366	16.6	7063	13.8
Fixed combinations of SABA and SAMA	666	4.7	1417	2.8
Fixed combinations of SABA and ICS	0	0	0	0
Fixed combinations of LABA and ICS	5404	38.0	14 600	28.5
Systemic glucocorticoids	4429	31.1	12 133	23.7
Systemic beta2‐agonists	66	0.5	242	0.5
Xanthines and adrenergics	109	0.8	256	0.5
Roflumilast	25	0.2	50	0.1
Nasal glucocorticoids	733	5.1	2620	5.1
Omalizumab	0	0	0	0
Leukotriene receptor antagonists	252	1.8	787	1.5
Cromoglicic acid	0	0	0	0
Nedocromil	0	0	0	0
Oxygen therapy	2092	14.7	5701	11.1
Nebuliser therapy	10	0.1	14	~0
Cardiovascular medications	10 658	74.9	37 750	73.8
Cardiac glycosides and antiarrhythmics, class I and III	937	6.6	2926	5.7
Vasodilators used in cardiac diseases	1066	7.5	3605	7.0
Cardiac stimulants and other cardiac preparations	71	0.5	209	0.4
Diuretics	5759	40.4	18 931	37.0
Peripheral vasodilators	0	0	<5	~0
Vasoprotective agents	348	2.4	1301	2.5
Beta blocking agents	3952	27.8	14 386	28.1
Calcium channel blockers	3261	22.9	11 841	23.1
Antihypertensives	173	1.2	553	1.1
Agents acting on the renin‐angiotensin system	5282	37.1	19 806	38.7
Angiotensin‐converting‐enzyme inhibitors	2719	19.1	10 064	19.7
Angiotensin II receptor antagonists	2656	18.7	10 137	19.8
Renin‐inhibitors	<5	~0	12	~0
Lipid‐modifying agents	5428	38.1	19 891	38.9
Statins (HMG‐CoA reductase inhibitors)	5334	37.5	19 539	38.2
Other lipid‐modifying agents	190	1.3	708	1.4
Statins, other combinations with acetylsalicylic acid	0	0	0	0
Antithrombotic agents	6887	48.4	23 956	46.8
Platelet aggregation inhibitors	4704	33.0	16 341	31.9
Systemic antibacterials	8214	57.7	26 105	51.0
Iron preparations	290	2.0	900	1.8
Proton pump inhibitors	4973	34.9	17 075	33.4
Drugs used for diabetes	1837	12.9	6742	13.2
Insulins	602	4.2	2053	4.0
Blood glucose–lowering drugs	1599	11.2	5912	11.6
Drugs for musculoskeletal system	1982	13.9	7242	14.2
Anti‐inflammatory and antirheumatic products, non‐steroidal^a^	1916	13.5	7041	13.8
Acetylsalicylic acid	89	0.6	273	0.5
Other antirheumatic agents^b^	0	0	0	0
Antidepressants	3228	22.7	10 753	21.0
Selective serotonin reuptake inhibitors	1845	13.0	6171	12.1
Antineoplastic agents	11	0.1	27	0.1
Immunosuppressants	196	1.4	738	1.4
Antivirals for systemic use	175	1.2	568	1.1
Hormone‐replacement therapy^c^	1082	7.6	4141	8.1
Drugs used in nicotine dependence, %	414	2.9	1210	2.4
COPD severity category				
Mild	802	5.6	5777	11.3
Moderate	3447	24.2	15 674	30.6
Severe	6651	46.7	20 444	40.0
Very severe	3339	23.4	9272	18.1
CCI score				
1	4622	32.5	16 865	33.0
2	2806	19.7	10 590	20.7
3+	6811	47.8	23 712	46.3
Hip fracture	144	1.0	436	0.9
Lung cancer	432	3.0	1000	2.0
Other markers of bad fall	91	0.6	343	0.7
Metastatic cancer	110	0.8	287	0.6
Pulmonary cachexia	69	0.5	168	0.3
Right‐sided heart failure	135	0.9	274	0.5
Number of hospitalisations within 180 days				
0	7448	52.3	33 178	64.8
1	4029	28.3	12 143	23.7
2	1519	10.7	3511	6.9
3–4	963	6.8	1874	3.7
5+	280	2.0	461	0.9
Number of hospitalisations within 365 days				
0	5906	41.5	26 565	51.9
1	4050	28.4	13 860	27.1
2	1959	13.8	5610	11.0
3–4	1539	10.8	3602	7.0
5+	785	5.5	1530	3.0
Number of hospitalisations for COPD within 90 days				
0	9702	68.1	41 596	81.3
1	3577	25.1	8189	16.0
2+	960	6.7	1382	2.7
Number of hospitalisations for COPD within 180 days				
0	8859	62.2	38 753	75.7
1	3882	27.3	10 058	19.7
2+	1498	10.5	2356	4.6
Number of COPD exacerbations within 90 days				
0	6260	44.0	28 582	55.9
1	4425	31.1	14 666	28.7
2	2354	16.5	5787	11.3
3+	1200	8.4	2132	4.2
Number of COPD exacerbations within 180 days				
0	4680	32.9	22 400	43.8
1	4129	29.0	14 909	29.1
2	2643	18.6	8196	16.0
3+	2787	19.6	5662	11.1

Abbreviations: CCI, Charlson Comorbidity Index; COPD, chronic obstructive pulmonary disease; HMG‐CoA, hydroxymethylglutaryl‐coenzyme A; ICS, inhaled corticosteroid; LABA, long‐acting beta agonist; LAMA, long‐acting muscarinic antagonist; Q1, quarter 1; Q3, quarter 3; SABA, short‐acting beta agonist; SAMA, short‐acting muscarinic antagonist; SD, standard deviation.

^a^ Anti‐inflammatory/antirheumatic agents in combination, specific antirheumatic agents.

^b^ Estrogens, progestogens, and progestogens and estrogens in combination.

^c^ Nonsteroidal anti‐inflammatory drugs.

### 3.2 Incidence rate ratios of myocardial ischaemia, cardiac arrhythmia, and all‐cause mortality and the impact of channelling on risk assessment

The number of events, IRDs, and IRRs of primary outcomes for cardiac arrhythmias or myocardial ischaemia associated with the use of olodaterol as compared with other LABAs are shown in Table 2. In these data, among users of olodaterol compared with users of other LABA, the IRR point estimates of the primary outcomes ranged from 0.96 to 1.65 in the analyses adjusted for additional variables, and 95% CI for some of the outcomes were wide. Further adjustment made by refitting the PS to include additional variables resulted in only slight changes to the results (Table 2).

**TABLE 2:** IRs (per 1000 person‐years), IRDs (per 1000 person‐years), and IRRs for each primary outcome (propensity score, trimmed analysis cohort)

Results	AF		SVT		VT		AMI		SACHD	
	Olodaterol	Other LABA	Olodaterol	Other LABA	Olodaterol	Other LABA	Olodaterol	Other LABA	Olodaterol	Other LABA
No. of patients	14 239	51 167	14 239	51 167	14 239	51 167	14 239	51 167	14 239	51 167
No. of events	246	725	19	38	29	80	56	137	41	147
Person‐years	5173	19 038	5247	19 253	5252	19 260	5238	19 202	5239	19 204
Crude IR (95% CI)^a^	48 (42–53)	38 (35–41)	4 (2–5)	2 (1–3)	6 (4–8)	4 (3–5)	11 (8–13)	7 (6–8)	8 (5–10)	8 (6–9)
Crude IRR (95% CI)^a^	1.25 (1.03–1.51)	Reference	1.83 (0.90–3.72)	Reference	1.33 (0.76–2.34)	Reference	1.50 (0.99–2.27)	Reference	1.02 (0.67–1.56)	Reference
Crude IRD (95% CI)^a^	9 (1–18)	Reference	2 (0–4)	Reference	1 (−1 to 4)	Reference	4 (0–7)	Reference	0 (−3 to 3)	Reference
Adj. IRR (95% CI)^b^	1.20 (0.98–1.47)	Reference	1.83 (0.90–3.74)	Reference	1.30 (0.71–2.36)	Reference	1.22 (0.79–1.87)	Reference	1.00 (0.64–1.56)	Reference
Adj. IRD (95% CI)^b^	8 (−1 to 16)	Reference	2 (−0 to 4)	Reference	1 (−2 to 4)	Reference	1 (−2 to 5)	Reference	0 (−3 to 3)	Reference
Adjusted IRR from the analysis of the primary outcomes, including additional variables in propensity score										
Adj. IRR (95% CI)^b^	1.14 (0.93–1.40)	Reference	1.65 (0.80–3.41)	Reference	1.32 (0.69–2.52)	Reference	1.26 (0.82–1.93)	Reference	0.96 (0.61–1.53)	Reference

Abbreviations: Adj., adjusted; AF, hospitalisation or hospital outpatient specialist visit for atrial fibrillation or flutter; AMI, hospitalisation for acute myocardial infarction; CI, confidence interval; IR, incidence rate; IRD, incidence rate difference; IRR, incidence rate ratio; LABA, long‐acting beta2‐agonist; SACHD, hospitalisation for serious acute coronary heart disease, including angina and other acute ischaemic heart disease events; SVT, hospitalisation or hospital outpatient specialist visit for supraventricular tachycardia (other than atrial fibrillation/flutter); VT, hospitalisation for ventricular tachycardia, including ventricular fibrillation/flutter and cardiac arrest.

^a^ Estimates were generated with a Poisson regression model, including exposure and the natural logarithm of the person‐years at risk as the offset.

^b^ Estimates were generated with a Poisson regression model, including exposure, the natural logarithm of the person‐years at risk as the offset, propensity score quintiles, and LAMA use at the index date as a covariate.

In the initial analysis, there was an increased risk of death among users of olodaterol compared with other LABAs, with an adjusted IRR of 1.63 (95% CI, 1.44–1.84) and an adjusted IRD of 59 per 1000 person‐years (95% CI, 43–74) (Table 3).

**TABLE 3:** IRs (per 1000 person‐years), IRDs (per 1000 person‐years), and IRRs for all‐cause mortality using various methods to control confounding

Analysis	Measure	Olodaterol Cohort	Other LABA Cohort
Overall population (initial model)	Number of patients	14 239	51 167
	Number of events	859	1.872
	Person‐years	5254	19 266
	Crude IR (95% CI)^a^	163 (153–174)	97 (93–102)
	Crude IRD (95% CI)^a^	66 (52–80)	Reference
	Adjusted IRD (95% CI)^b^	59 (43–74)	Reference
	Crude IRR (95% CI)^a^	1.68 (1.50–1.88)	Reference
	Adjusted IRR (95% CI)^b^	1.63 (1.44–1.84)	Reference
Overall population (adjusting outcome model by specific additional variables)	Adjusted IRR (95% CI)^c^	1.40 (1.24–1.59)	Reference
LABA/LAMA, LABA naive	Number of patients	5677	20 514
	Number of events	357	777
	Adjusted IRR (95% CI)^d^	1.48 (1.23–1.78)	Reference
LABA/LAMA LABA naive, no hospitalisation for COPD in the last 90 days	Number of patients	3843	14 029
	Number of events	137	370
	Adjusted IRR (95% CI)^e^	1.26 (0.97–1.64)	Reference
LABA/LAMA, LABA naive, using 10% trimming	Number of patients	4191	11 504
	Number of events	231	473
	Adjusted IRR (95% CI)^f^	1.27 (1.03–1.57)	Reference
Overall population, using overlap weights	Number of events	379	302
	Weighted adjusted IRR (95% CI)^g^	1.32 (1.19–1.48)	Reference
	Weighted adjusted HR (95% CI)^h^	1.29 (1.16–1.44)	Reference

Abbreviations: CI, confidence interval; COPD, chronic obstructive pulmonary disease; HR, hazard ratio; IR, incidence rate; IRD, incidence rate difference; IRR, incidence rate ratio; LABA, long‐acting beta2‐agonist; LAMA, long‐acting muscarinic antagonist; SABA, short‐acting beta agonist; SAMA, short‐acting muscarinic antagonist.

*Note*: The other LABA cohort comprised inhaled long‐acting beta2‐agonists other than olodaterol; IR and IRD are per 1000 person‐years.

^a^ Using Poisson regression model, including exposure and the natural logarithm of the person‐years at risk as the offset.

^b^ Using a Poisson regression model, including exposure, the natural logarithm of the person‐years at risk as the offset, the propensity score quintiles, and LAMA use at the index date as a binary covariate.

^c^ Using a Poisson regression model, including exposure, the natural logarithm of person‐years at risk as the offset, propensity score quintiles, LAMA use at the index date as a binary covariate and further adjusted for variables that were not included in the propensity score calculation but were considered to be confounders: number of hospitalisations in the 180 days before index date, number of COPD hospitalisations in the 90 days before index date, number of COPD exacerbations in the 180 days before index date, and history of lung cancer.

^d^ Using a Poisson regression model, including exposure, the natural logarithm of person‐years at risk as the offset, the propensity score deciles, and adjustment for number of all‐cause hospitalisation (180 days), number of COPD exacerbations (180 days), previous LAMA use, previous oxygen use, and previous ICS use with further adjustment for unbalanced variables: prior use of respiratory medications, fixed combinations of SABA and SAMA, systemic antibacterials, COPD severity, number of all‐cause hospitalisations in the 365 days before index date, number of COPD hospitalisations in the 90 days before index date, number of COPD hospitalisations in the 180 days before index date, and number of COPD exacerbations in the 90 days before index date.

^e^ Using a Poisson regression model, including exposure, the natural logarithm of person‐years at risk as the offset, propensity score deciles, and adjustment for number of all‐cause hospitalisation (180 days), number of COPD exacerbations (180 days), previous LAMA use, previous oxygen use, and previous ICS use, with further adjustment for imbalanced variables: prior use of respiratory medications and COPD severity.

^f^ Using a Poisson regression model, including exposure, the natural logarithm of person‐years at risk as the offset, propensity score decile, and number of COPD hospitalisations (90 days).

^g^ Using a Poisson regression model, including exposure, the natural logarithm of person‐years at risk as the offset, and LAMA at index date as a binary covariate.

^h^ Using Cox proportional hazard ratios and adjusted by LAMA use at index date as a binary covariate.

Several findings indicated channelling of olodaterol to patients with more severe COPD, comorbidity, and likely a worse prognosis. The mortality rates in both cohorts as well as the probability of receiving olodaterol increased with each PS quintile. Ascertainment of additional potential risk factors for death (such as proxies for frailty and number of hospitalisations and exacerbations, either overall or due to COPD) showed that the distributions of these variables were imbalanced between study cohorts within PS strata (Table S10), confirming that there was channelling of olodaterol prescriptions to a high‐risk population. Patients receiving olodaterol had a higher prevalence of prior hospitalisations for any cause, a higher prevalence of prior hospitalisations for COPD, and a higher prevalence of COPD exacerbations in the prior 90 days and 180 days (Table 1).

In a first step to reduce confounding, these prognostic variables were included in the analysis model. With the addition of these imbalanced variables into the model, the IRR decreased from the initially observed 1.63 adjusted IRR to an adjusted IRR of 1.40 (95% CI, 1.24–1.59).

The proportion of patients on a LABA/LAMA FDC differed between the study cohorts (92% vs. 63%) (Figure 1). When restricting the population to users of FDCs of LABA/LAMA who were LABA naive and calculating a new PS that included the additional variables, the adjusted IRR of death among users of olodaterol compared with other LABAs decreased to 1.48 (95% CI, 1.23–1.78). For this analysis, baseline characteristics still showed some imbalance in important risk factors, with patients in the olodaterol cohort having more hospitalisations for COPD in the last 90 days (32% in the olodaterol cohort versus 18% in the other LABA cohort) (Tables S12 and S13).

The study population was then further restricted to those patients with no hospitalisations for COPD in the last 90 days (Table S14). For this comparison, there were no notable imbalances (Table S15), and the adjusted IRR of death was 1.26 (95% CI, 0.97–1.64) for patients who used olodaterol compared with patients who used other LABAs.

Similar results were obtained by alternative approaches to obtain more comparable patient populations. First, trimming the tails of the PS distributions at 10% and 90% gave an adjusted IRR of death of 1.27 (95% CI, 1.03–1.57) based on a patient population with a more similar distribution of COPD severity and hospitalisations at baseline (Table S16). Second, using PSs with overlap weights, we obtained a similar effect estimate, with an adjusted hazard ratio of 1.29 (95% CI, 1.16–1.44) and an adjusted IRR of 1.32 (95% CI, 1.19–1.48) (Table 3) in the overall study population.

In the bias analysis, we considered two scenarios, beginning with an apparent IRR of 1.40 and 1.26, obtained from the restricted populations of patients who used LABA/LAMA, were LABA naive, and had no COPD hospitalisations in the last 90 days. In both scenarios, the additionally adjusted IRR point estimates attenuated to 1.0 when the prevalence of the unmeasured factors differed between the olodaterol and “other LABA” cohorts by approximately 10% (see Online Supporting Information for more details, Tables S17 and S18).

## 4 DISCUSSION

In this study among adults aged ≥40 years with COPD, a similar risk of ischaemic events and arrhythmias was observed among users of olodaterol compared with users of other LABAs. Despite observed differences in the baseline characteristics by cohort and evidence of preferential prescribing of olodaterol to a population with more severe COPD, results remained consistent when adjusting for additional variables that were not initially included. This analysis suggests that those additional variables did not have an important effect on the primary analysis and outcomes and that most main confounders were reasonably well controlled. Confounding by unmeasured characteristics such as smoking, dyspnoea, level of airway obstruction, or BMI remains possible.

In the initial analyses, new use of olodaterol compared with new use of other LABAs was associated with an increased risk of death due to any cause. Additional analyses suggested that these findings were affected by channelling bias. We suspect that at least two possible reasons may explain channelling of olodaterol prescribing. First, clinicians may tend to use newly marketed drugs on subgroups of patients with more severe COPD and poor response to established drugs. Second, patients with many COPD exacerbations or hospitalisations are likely to be referred to pulmonologists, who, relative to (for instance) general practitioners, are more inclined to prescribe newly marketed drugs for COPD treatment. Both the matched cohort study design and the PS methodology aimed to create comparable treatment cohorts for the incidence analyses. We interpret the observed association between olodaterol use and mortality as likely to be spurious, confounded by baseline differences in disease state related to COPD severity and frailty between study cohorts, which were not accounted for in the primary analysis.

The results of the additional analyses performed to further evaluate the initial study results showed strong attenuation of the IRR for the risk of death after adjusting for additional variables, consistent with the presence of channelling bias. The considerable discrepancy between the effect estimates indicated considerable confounding. This considerable confounding implies that residual confounding may also be considerable due to unmeasured or imperfectly measured factors. Additional analyses, which (1) restricted the population to users of LABA/LAMA who were LABA naive and further trimmed this population at 10% of the tails of the PS distributions, (2) restricted the population to those with no hospitalisations for COPD in the last 90 days, or (3) used overlap weights, resulted in cohorts that were more comparable and produced substantial attenuation of the estimated effect on risk of death. A bias analysis indicated that adjustment for unmeasured clinical factors could fully attenuate the mortality association under a plausible range of assumptions about the distribution of these factors between cohorts and the strength of association between these factors and mortality.

Only a subset of the study population had lengthy exposure, thus limiting inferences about long‐term safety of olodaterol. However, important additional context for the interpretation of this association can be found in the results of randomised clinical trials of olodaterol, both as monotherapy and in combination with tiotropium. In four large, long‐term (48 weeks), parallel‐group, placebo‐ and active comparator–controlled, phase 3 olodaterol (Striverdi®) trials, no strong association was observed between olodaterol or formoterol and death compared with placebo (olodaterol 5 μg: relative risk [RR], 0.94 [95% CI, 0.44–2.02]; olodaterol 10 μg: RR, 1.24 [95% CI, 0.60–2.55]; and formoterol 12 μg: RR, 0.87 [95% CI, 0.37–2.05]). Similarly, outcomes of major adverse cardiovascular events, AMI, cardiac tachyarrhythmias, and ventricular tachyarrhythmias were comparable across treatment groups.^6^ In three large, long‐lasting trials (52 weeks) of olodaterol combined with tiotropium (Stiolto Respimat®), the adjusted rate ratio of death for combination therapy versus tiotropium alone was 0.93 (95% CI, 0.71–1.21).^4^

Our study had several limitations. The mortality results are likely subject to a form of selection bias called channelling bias, which occurs when different therapies are preferentially prescribed to groups of patients with different baseline disease states.^24^ In the absence of a pure new‐user design based on drug class, the fact that users of olodaterol were more frequently prior users of other LABAs may reflect that patients in the olodaterol cohort were in a more advanced stage of COPD and, as a consequence, had a higher risk of death. Due to imbalances in important risk factors for death (e.g., COPD severity, COPD hospitalisations, or COPD exacerbations) that were observed between the olodaterol and “other LABA” cohorts and the fact that other important risk factors for mortality (e.g., low BMI, smoking status, airflow impairment, dyspnoea) could not be measured in the Danish registers, the risk of substantial residual confounding was high.^23^, ^25^, ^26^ Compared with the initial analysis, alternative approaches showed substantial attenuation of the IRR after adjustment by approximately 25%. Taken together, these results indicate that the observed association between olodaterol and all‐cause mortality may well be the result of confounding. As the amount of residual confounding tends to reflect the overall confounding in the data,^27^, ^28^, ^29^ adjustment for measured confounding was unlikely to compensate fully for a potential bias introduced by systematic channelling of patients with more severe COPD towards olodaterol treatment. Although the target study size was reached, the precision of the adjusted IRRs (as assessed by the width of 95% CIs) for some outcomes, such as SVT and VT, was low due to comparatively few events for those outcomes. Because diagnoses in the Danish National Patient Registry are hospital based, it is likely that patients with milder forms of COPD who had been managed exclusively in primary care were not included in this study.

Results from this cohort study conducted in patients with COPD indicated that the use of olodaterol compared with the use of “other LABA” was not associated with an important increased risk of cardiac arrhythmias or myocardial ischaemia as measured by the adjusted IRDs. Compared with use of “other LABA,” use of olodaterol was associated with an increased risk of all‐cause mortality, which seemed largely attributable to differences in the baseline characteristics of the populations stemming from channelling (preferential prescribing).^30^ This interpretation is supported by evidence from large, long‐term clinical trials performed in parallel that indicated no important effect on all‐cause mortality.

## CONFLICT OF INTEREST

This study was conducted under a research contract between Boehringer Ingelheim and RTI Health Solutions and was funded by Boehringer Ingelheim. CR, JA, KJR, and DM are employees of RTI Health Solutions. JM, FV, UB, and KZ are employees of Boehringer Ingelheim. DKF, KL, and VE are employees of Aarhus University and participated in this study under an institutional research agreement between RTI Health Solutions and Aarhus University.

## ETHICS STATEMENT

This study was approved by the Danish Data Protection Agency (record number: 2016‐051‐000001, serial number 810). According to Danish legislation, informed consent or approval from an ethics committee is not required for registry‐based studies.

## ROLE OF THE SPONSOR

Boehringer Ingelheim participated in the design and conduct of the study; interpretation of the data; and review of the manuscript. The contract between Boehringer Ingelheim granted RTI Health Solutions independent publication rights.

## Supporting information


**Appendix** **S1:** Supporting Information
