# Supplementary material for: Cardiovascular events and all‐cause mortality in patients with chronic obstructive pulmonary disease using olodaterol and other long‐acting beta2‐agonists
Source: Pharmacoepidemiol Drug Saf. 2022 May 13;31(8):827–39. doi: 10.1002/pds.5432 (PMC9545725; doi:10.1002/pds.5432)
Supplement: Supplementary file 1 — Appendix S1: Supporting Information [file PDS-31-827-s001.docx]

# Supporting information

## Supplemental Methods

### Assessment of Balance

To check for balance of the covariates included in the propensity score models between the treatment groups (olodaterol and “other LABA”), the standardised bias (SB) of “other LABA” versus olodaterol for each covariate within each propensity score stratum was calculated. The SB compares the difference in means or proportions in units of the standard deviation. All variables were categorical. The overall SB for categorical variables was computed as the difference in proportions of each level of the covariate (“other LABA” minus olodaterol) divided by the standard deviation of the covariate in the “other LABA” cohort. The strata-specific propensity score was computed using the same approach within each stratum. Within each propensity score stratum, the SB was expected to be low, as olodaterol initiators and “other LABA” initiators had similar values of the propensity score. The average SB was computed as the results of the average of the SB across deciles, keeping the signs.

Imbalance between the cohorts for a specific baseline characteristic was considered important if the overall SB (i.e., the difference in proportions of each level of the covariate [“other LABA” minus olodaterol] divided by the standard deviation of the covariate in the “other LABA” cohort) was greater than 0.20. To reach better balance in the distribution of the baseline characteristics, in the post hoc analysis the threshold used for overall and average SB was 0.1.

### Additional Analysis

| Objective | Population and Outcome | Methods/Analysis |
| --- | --- | --- |
| Assess robustness of results after including additional potential confounders | Overall population, primary outcomes | 1: Estimate the overall IRR for the primary outcomes after modifying the propensity score model by adding variables considered post hoc (Table 2) |
|  | Overall population, all-cause mortality | 2: Estimate the overall IRR for all-cause mortality after modifying the propensity score model by adding variables considered post hoc (Table S7) |
|  | LABA monotherapy users, all-cause mortality | 3: Estimate the overall IRR for all-cause mortality restricting the population to monotherapy users (*population 1,* matched 1:4; *population* *2,* including all available comparators)^a^ (Table S8) |
| Assess robustness of results with respect to the analysis techniques used to evaluate all-cause mortality | Overall population, all-cause mortality outcome | 4: Estimate propensity score stratum–specific IRR, adjusting for additional variables considered post hoc, and obtain overall adjusted IRR using inverse variance weighted average (Table S7)  Estimate the overall adjusted IRR:  5: Using fine stratification (propensity score ventiles, i.e., 20 strata)^b^ (Table S7)  6a: Using propensity score matching and Mantel-Haenszel matched-pairs methods^b^ (Table S7)  6b: Using propensity score matching to estimate the adjusted hazard ratio using Cox regression^b^ (Table S7)  7: Using intention-to-treat analysis instead of as-treated analysis (Table S7) |
| Restrict population to reduce confounding | Population of users of FDC LABA/LAMA who were LABA naive, all-cause mortality outcome | 8: Restricting the population to users of FDC LABA/LAMA who were LABA naive (Table 3)  9: Restricting the population to users of FDC LABA/LAMA who were LABA naive and had no hospitalisations for COPD in the last 90 days prior to index date (Table 3)  10: Restricting the population to users of FDC LABA/LAMA who were LABA naive and applying 10% (instead of 2,5%) trimming in each extreme of the propensity score distribution (Table 3) |
|  | Overall population | 11: Applying overlap weights method to achieve a population with clinical equipoise^b^ (Table 3) |

COPD = chronic obstructive pulmonary disease; FDC = fixed-dose combination; IRR = incidence rate ratio; LABA = long-acting beta2-agonist; LAMA = long-acting muscarinic antagonist.

^a^Two approaches were used to select users of monotherapy. In the first approach (population 1), the study population was selected using the same approach as in the main analysis but restricting the population to users of monotherapy (i.e., same inclusion and exclusion criteria, then restricted to users of olodaterol monotherapy, and finally matched 1:4 with replacement by age, sex, and calendar year with users of “other LABA” monotherapy). In the second approach (population 2), the study population was selected using the same approach, but no matching was performed (i.e., all users of “other LABA” monotherapy that fulfilled the inclusion and exclusion criteria were included).

^b^The study population was selected using the same inclusion and exclusion criteria as in the main study report, except that the population was limited to the first eligibility period in which the patient qualified for cohort entry; patients were not allowed to re-enter the other cohort even if they later met criteria. In addition, patients were not matched by age, sex, and calendar year.

## Supplemental Tables

Table S1. Codes to Define Chronic Obstructive Pulmonary Disease

| ICD‑10 code | Description |
| --- | --- |
| J41.0 | Simple chronic bronchitis |
| J41.1 | Mucopurulent chronic bronchitis |
| J41.9 | Mixed simple and mucopurulent chronic bronchitis |
| J42 | Unspecified chronic bronchitis |
| J43.0 | MacLeod syndrome |
| J43.1 | Panlobular emphysema |
| J43.2 | Centrilobular emphysema |
| J43.8 | Other emphysema |
| J43.9 | Emphysema, unspecified |
| J44.0 | Chronic obstructive pulmonary disease with acute lower respiratory infection |
| J44.1 | Chronic obstructive pulmonary disease with acute exacerbation, unspecified |
| J44.8 | Other specified chronic obstructive pulmonary disease |
| J44.9 | Chronic obstructive pulmonary disease, unspecified |

ICD‑10 = *International Statistical Classification of Diseases and Related Health Problems, 10th Revision*.

Table S2. Codes to Define Study Outcomes

| **Outcome** | **ICD‑10 code** | **Description** |
| --- | --- | --- |
| Atrial Fibrillation and Flutter (AF) | I48 | Atrial fibrillation and flutter |
| Supraventricular tachycardia (SVT) | I47.1 | Supraventricular tachycardia |
| Ventricular tachycardia (VT) | I47.0 | Re-entry ventricular arrhythmia |
| Ventricular tachycardia (VT) | I47.2 | Ventricular tachycardia |
| Ventricular tachycardia (VT) | I49.0 | Ventricular fibrillation and flutter |
| Ventricular tachycardia (VT) | I46.0 | Cardiac arrest with successful resuscitation |
| Ventricular tachycardia (VT) | I46.1 | Sudden cardiac death, so described |
| Ventricular tachycardia (VT) | I46.9 | Cardiac arrest, unspecified |
| Acute myocardial infarction (AMI) | I21.0 | Acute transmural myocardial infarction of anterior wall |
| Acute myocardial infarction (AMI) | I21.1 | Acute transmural myocardial infarction of inferior wall |
| Acute myocardial infarction (AMI) | I21.2 | Acute transmural myocardial infarction of other sites |
| Acute myocardial infarction (AMI) | I21.3 | Acute transmural myocardial infarction of unspecified site |
| Acute myocardial infarction (AMI) | I21.4 | Acute subendocardial myocardial infarction |
| Acute myocardial infarction (AMI) | I21.9 | Acute myocardial infarction, unspecified |
| Angina | I20.0 | Unstable angina |
| Angina | I20.1 | Angina pectoris with documented spasm |
| Angina | I20.8 | Other forms of angina pectoris |
| Angina | I20.9 | Angina pectoris, unspecified |
| Other acute ischaemic heart diseases | I24.0 | Coronary thrombosis not resulting in myocardial infarction |
| Other acute ischaemic heart diseases | I24.1 | Dressler syndrome |
| Other acute ischaemic heart diseases | I24.8 | Other forms of acute ischaemic heart disease |
| Other acute ischaemic heart diseases | I24.9 | Other acute ischaemic heart diseases |

ICD‑10 = *International Statistical Classification of Diseases and Related Health Problems, 10th Revision*.

Table S3. Codes for Other Comorbidities

| Disease description | ICD‑10 code and NCSP codes |
| --- | --- |
| **Cardiovascular diseases** | I00-I99 |
| **Ischaemic heart disease** | I20-I25 or coronary reperfusion surgery and procedures |
| Angina pectoris | I20 |
| Acute myocardial infarction | I21 |
| Other acute or subacute ischaemic heart disease | I22-I24 |
| Chronic ischaemic heart disease | I25 |
| Coronary reperfusion surgery and procedures | KFNG, KFNF, KFNA-KFNE, KFNH20 |
| **Conduction disorders** | I44-I45 |
| **Cardiac arrest** | I46 |
| **Arrhythmias** | I47-I49 |
| Paroxysmal tachycardia | I47 |
| Ventricular tachycardia | I47.0, I47.2 |
| Supraventricular tachycardia and unspecified | I47.1 |
| Atrial fibrillation and flutter | I48 |
| Other cardiac arrhythmias | I49 |
| Ventricular fibrillation and flutter | I49.0 |
| Other cardiac arrhythmias | I49.1-I49.9 |
| **Heart failure** | I50 |
| **Cerebrovascular disease** | I60-I69, G45, G46 |
| Cerebral haemorrhage (subarachnoid, intracerebral, other non-traumatic) | I60-I62 |
| Cerebral infarction and stroke | I63, I64, G46.5 |
| Transient ischaemic attack | G45 |
| Other cerebrovascular disease and sequelae of cerebrovascular disease | I65-I69 |
| **Hypertension and hypertensive heart disease** | Combination treatment of at least two redeemed prescriptions for different types of the following classes of antihypertensive drugs within 180 days prior to the index date:   - α-adrenergic blockers: ATC: C02A, C02B, C02C; - Non-loop diuretics ATC: C02DA, C02L, C03A, C03B, C03D, C03E, C03X, C07C, C07D, C08G, C09BA, C09DA, C09XA52; - Vasodilators, ATC: C02DB, C02DD, C02DG, C04, C05; - β-blockers, ATC: C07; - Calcium channel blockers, ATC: C07F, C08, C09BB, C09DB; and - Renin-angiotensin system inhibitors ATC: C09. |
| **Diseases of arteries, arterioles, and capillaries** | I70-I79 and peripheral arterial revascularisation procedures |
| Peripheral arterial revascularisation procedures | KPA-KPG |
| Other form of heart diseases | I00-I09, I30-I43, I80-I99 |
| **Hyperlipidaemia** | E78 |
| **Diabetes mellitus** | E10-E14, O24 (except O24.4), G63.2, H36.0, N08.3  OR  ATC code: A10A, A10B |
| **Renal disease** | N00-N39 |
| Chronic kidney disease | N18 |
| Other renal disorders | N00-N17, N19, N25-N39 |
| **Anaemias** | D50-D64 |
| Nutritional anaemias | D50-D53 |
| Iron deficiency anaemias | D50 |
| Other anaemias | D55-D64 |
| **Peptic ulcer disease** | K25-K28 |
| **Liver disease** | K70-K77 |
| **Osteoporosis** | M80-M82 |
| **Rheumatoid arthritis and other inflammatory arthropathies** | M05-M14 |
| **Systemic connective tissue diseases** | M30-M36 |
| **Malignancy** | C00-C97 |
| **Depressive disorders** | F32-F33 |
| **Pregnancy (at the index date)** | O00-O99, O82 Among these, different ICD-10 codes were checked in different periods relative to index date |

ATC = Anatomical Therapeutic Chemical; ICD‑10 = *International Statistical Classification of Diseases and Related Health Problems, 10th Revision*; NCSP = Nordic Medico-Statistical Committee Classification of Surgical Procedures.

Table S4. Codes to Define Medications: Inhaled Selective Beta2-Adrenoreceptor Agonists, Anticholinergics, and Glucocorticosteroids

| ATC code | Name | ATC code | Name |
| --- | --- | --- | --- |
| Inhaled short-acting beta2-agonists | | Inhaled long-acting muscarinic antagonists | |
| R03AC02 | Salbutamol | R03BB04 | Tiotropium bromide |
| R03AC03 | Terbutaline | R03BB05 | Aclidinium bromide |
| R03AC04 | Fenoterol | R03BB06 | Glycopyrronium bromide |
| R03AC05 | Rimiterol | R03BB07 | Umeclidinium bromide |
| R03AC06 | Hexoprenaline | R03BB54 | Tiotropium bromide, combinations |
| R03AC07 | Isoetarine | Inhaled glucocorticosteroids | |
| R03AC08 | Pirbuterol | R03BA01 | Beclometasone |
| R03AC09 | Tretoquinol | R03BA02 | Budesonide |
| R03AC10 | Carbuterol | R03BA03 | Flunisolide |
| R03AC11 | Tulobuterol | R03BA04 | Betamethasone |
| R03AC14 | Clenbuterol | R03BA05 | Fluticasone |
| R03AC15 | Reproterol | R03BA06 | Triamcinolone |
| R03AC16 | Procaterol | R03BA07 | Mometasone |
| R03AC17 | Bitolterol | R03BA08 | Ciclesonide |
| Inhaled long-acting beta2-agonists | | R03BA09 | Fluticasone furoate |
| R03AC12 | Salmeterol |  |  |
| R03AC13 | Formoterol |  |  |
| R03AC18 | Indacaterol |  |  |
| R03AC19 | Olodaterol |  |  |
| Inhaled short-acting muscarinic antagonists | |  |  |
| R03BB01 | Ipratropium bromide |  |  |
| R03BB02 | Oxitropium bromide |  |  |

ATC = Anatomical Therapeutic Chemical.

Source: WHO Collaborating Centre for Drug Statistics Methodology. ATC/DDD Index 2013. Updated 20 December 2012. Available at: website: whocc.no/atc_ddd_index/. Accessed 21 January 2013.

Table S5. Anatomical Therapeutic Chemical Codes for Comedications

| Medication description | ATC code^a^ |
| --- | --- |
| Respiratory medications |  |
| Inhaled short-acting muscarinic antagonists (SAMAs) | See Table S4 for ATC codes |
| Inhaled long-acting muscarinic antagonists (LAMAs) | See Table S4 for ATC codes |
| Inhaled short-acting beta2-agonists (SABAs) | See Table S4 for ATC codes |
| Inhaled long-acting beta2-agonists (LABAs) | See Table S4 for ATC codes |
| Inhaled glucocorticosteroids (ICS) | See Table S4 for ATC codes |
| Fixed combinations of SABA and SAMA | R03AL01, R03AL02 |
| Fixed combinations of SABA and ICS | R03AK13 |
| Fixed combinations of LABA and ICS | See Table S4 for ATC codes |
| Systemic glucocorticosteroids | H02AB |
| Systemic beta2-agonists | R03CC |
| Xanthines and adrenergics | R03DA, R03DB |
| Roflumilast | R03DX07 |
| Nasal glucocorticosteroids | R01AD |
| Omalizumab | R03DX05 |
| Leukotriene receptor antagonists | R03DC |
| Cromoglicic acid | R03BC01 |
| Nedocromil | R03BC03 |
| Oxygen therapy | Treatment codes: BGXA5 (all subcodes), BGKH |
| Nebuliser therapy | Treatment code: BGXA10 |
| Cardiovascular medications | All codes listed below in section Cardiovascular medications |
| Cardiac glycosides and antiarrhythmics, Class I and III | C01A, C01B |
| Vasodilators used in cardiac diseases | C01D |
| Cardiac stimulants and other cardiac preparations | C01E, C01C |
| Diuretics | C03 |
| Peripheral vasodilators | C04 |
| Vasoprotective agents | C05 |
| Beta blocking agents | C07 |
| Calcium channel blockers | C08 |
| Antihypertensives | C02 |
| Agents acting on the renin-angiotensin system | C09 |
| Angiotensin-converting-enzyme inhibitors | C09A, C09B |
| Angiotensin II receptor antagonists | C09C, C09D |
| Renin-inhibitors | C09X |
| Lipid-modifying agents | C10 |
| HMG-CoA reductase inhibitors (statins) | C10AA |
| Other lipid-modifying agents | C10AB, C10AC, C10AD, C10AX, |
| HMG-CoA reductase inhibitors (statins), other combinations with acetylsalicylic acid | C10BX |
| Antithrombotic agents | B01 |
| Platelet aggregation inhibitors | B01AC |
| Systemic antibacterials | J01 |
| Iron preparations | B03A |
| Proton pump inhibitors | A02BC |
| Drugs used in diabetes | A10 |
| Insulins | A10A |
| Blood glucose–lowering drugs | A10B, A10X |
| Drugs for musculoskeletal system | M01A, N02BA, M01B, M01C |
| Anti-inflammatory and antirheumatic products, non-steroids (non-steroidal anti-inflammatory drugs) | M01A |
| Acetylsalicylic acid (other analgesics and antipyretics) | N02BA |
| Other antirheumatic agents: anti-inflammatory/antirheumatic agents in combination, specific antirheumatic agents | M01B-M01C |
| Antidepressants | N06A |
| Selective serotonin reuptake inhibitors | N06AB |
| Antineoplastic agents | L01 |
| Immunosuppressants | L04 |
| Antivirals for systemic use | J05 |
| Hormone-replacement therapy: Estrogens, progestogens, progestogens and estrogens in combination | G03C, G03D, G03F |
| Drugs used in nicotine dependence | N07BA |

ATC = Anatomical Therapeutic Chemical.; HMG-CoA = hydroxymethylglutaryl-coenzyme A; ICS = inhaled glucocorticosteroid; LABA = long-acting beta2-agonist; LAMA = long-acting muscarinic antagonist; SABA = short-acting beta2-agonist; SAMA = short-acting muscarinic antagonist.

a The national drug code of each database country was used to identify medications without an individual ATC code.

Table S6. Criteria for Defining COPD Severity Categories

| **Severity category** | **Definition** |
| --- | --- |
| Mild | Less than two dispensings of drugs for obstructive airway disease of the same class in any 6-month interval in the 12 months before the index date (i.e., 0 or 1 prescriptions/dispensings of the same COPD drug class in the 12 months before the index date, or if ≥ 2 dispensings were in the same drug class, the dispensings had to be more than 180 days apart) |
| Moderate | Regular bronchodilator treatment, defined as having at least two dispensings of the same COPD drug class in the 12 months before the index date with an interval of ≤ 180 days between dispensings^a,b^ |
| Severe | Occurrence of at least one of the following events in the 12 months before the index date: |
|  | ·    Hospitalisation for COPD exacerbation^a^ |
|  | ·    At least two COPD exacerbations without hospitalisation, where COPD exacerbation was defined by any of the following^c^: |
|  | -     A course of antibiotics for lower respiratory tract infection (acute bronchitis or pneumonia)^a^ |
|  | -     A course of systemic glucocorticoid for the treatment of COPD exacerbation^a^ |
|  | -     A diagnosis of COPD exacerbation without a hospitalisation |
| Very severe | Occurrence of at least one of the following events in the 12 months before the index date unless another time period was specified: |
|  | ·    Oxygen therapy^a,b^ |
|  | ·    Dispensed nebuliser therapy^a,b^ |
|  | ·    Diagnosis of emphysema at any time before the index date^c^ |

COPD = chronic obstructive pulmonary disease.

^a^Severity criterion also included in definition from Curkendall et al., 2006.^1^

^b^Severity criterion also included in definition from Soriano et al., 2001.^2^

^c^Severity criteria modified to align with the Global Initiative for Chronic Obstructive Lung Disease (GOLD) 2016 definition for severity categories C and D.^3^ If two or more of these criteria occur within a 21-day time period, they will be considered part of the same exacerbation episode and counted only once. If one or more of these criteria occur within a 21-day period of a hospitalisation for COPD, this will be considered part of the same “COPD exacerbation with hospitalisation” episode.

Sources: modified from Verhamme et al., 2012^4^; Soriano et al., 2001^2^; Curkendall et al., 2006^1^; and GOLD, 2016.^3^

Table S7. IRRs and HRs for All-Cause Mortality, Overall Population, Using Different Analysis Methods

| **Method** | **Measure** | **Olodaterol cohort** | **Other LABA cohort** |
| --- | --- | --- | --- |
| Adjusting PS strata by specific additional variables, and estimated using inverse variance weighted average | Adjusted IRR (95% CI)^a^ | 1.42  (1.25-1.62) | Reference |
| Including additional variables in PS | Adjusted IRR (95% CI)^b^ | 1.52  (1.34-1.72) | Reference |
| Using fine strata and Poisson | Adjusted IRR (95% CI)^c^ | 1.42  (1.26-1.59) | Reference |
| Using fine strata and estimated through Mantel-Haenszel pooled estimates of the PS ventile–specific IRRs | Adjusted IRR (95% CI)^d^ | 1.46  (1.29-1.65) | Reference |
| In a PS match population and estimated through Mantel-Haenszel stratified by matched pairs | Adjusted IRR (95% CI)^e^ | 1.40  (1.28-1.53) | Reference |
| In a PS match population and estimated through Cox | Adjusted HR (95% CI)^f^ | 1.62  (1.36-1.94) | Reference |
| Using ITT approach | Adjusted IRR (95% CI)^g^ | 1.49  (1.37-1.61) | Reference |

CI = confidence interval; COPD = chronic obstructive pulmonary disease; HR = hazard ratio; LABA = long-acting beta2-agonist; LAMA = long-acting muscarinic antagonist; IRR = incidence rate ratio; ITT = intention to treat; PS = propensity score.

Note: The other LABA cohort comprised inhaled LABAs other than olodaterol.

^a^ Derived from stratum-specific IRR estimated using a Poisson regression model, including exposure, the natural logarithm of person-years at risk as the offset, PS quintiles, LAMA use at the index date as a binary covariate and further adjusted for variables that were not included in the PS calculation but were considered to be confounders: number of hospitalisations in the 180 days before the index date, number of COPD hospitalisations in the 90 days before the index date, number of COPD exacerbations in the 180 days before the index date, and history of lung cancer.

^b^ Using a Poisson regression model, including exposure, the natural logarithm of the person-years at risk as the offset, the PS quintiles, and LAMA use at the index date as a binary covariate. The PS model was refitted to include all additional variables measured post hoc.

^c^ From a Poisson regression model including exposure, the natural logarithm of the person-years at risk as the offset, the PS ventile, and LAMA at the index date.

^d^ Mantel-Haenszel pooled estimates of the PS ventile–specific IRRs, adjusted for LAMA at the index date.

^e^ Mantel-Haenszel (stratified by matched pairs).

^f^ Hazard ratio adjusted for LAMA use at the index date.

^g^ From a Poisson regression model including exposure, the natural logarithm of the person-years at risk as the offset, the PS quintiles, and LAMA use at the index date as a binary covariate.

Table S8. IRs (per 1,000 person-years), IRDs (per 1,000 person-years), and IRRs for All-Cause Mortality by PS-Trimmed Analysis Monotherapy Cohort

|  | **Olodaterol cohort** | **Other LABA cohort** |
| --- | --- | --- |
| **Monotherapy population 1^a^** | | |
| Number of patients | 366 | 1,167 |
| Number of events | 15 | 36 |
| Person-years | 126.7 | 435.6 |
| Crude IR (95% CI) | 118 (58-178) | 83 (56-110) |
| Crude IRR (95% CI) | 1.43 (0.76-2.70) | Reference |
| Crude IRD (95% CI) | 36 (−33 to 104) | Reference |
| Adjusted IRR (95% CI), (model 1)^b^ | 1.56 (0.81-2.99) | Reference |
| Adjusted IRD (95% CI), (model 1)^b^ | 42 (−26 to 111) | Reference |
| Adjusted IRR (95% CI), (model 2)^c^ | 1.59 (0.82-3.08) | Reference |
| Adjusted IRD (95% CI), (model 2)^c^ | 40 (−23 to 104) | Reference |
| Mantel-Haenszel pooled estimates of the PS ventile–specific IRRs | 1.57 (0.83-3.00) | Reference |
| Mantel-Haenszel pooled estimates of the PS ventile–specific IRDs | 46.1 (−25.2 to 117.5) | Reference |
| **Monotherapy population 2^d^** | | |
| Number of patients | 378 | 7,664 |
| Number of events | 16 | 245 |
| Person-years | 126.2 | 2,702.2 |
| Crude IR (95% CI) | 127 (65-189) | 91 (79-102) |
| Crude IRR (95% CI) | 1.40 (0.83-2.36) | Reference |
| Crude IRD (95% CI) | 36 (−29 to 101) | Reference |
| Adjusted IRR (95% CI), (model 1)^e^ | 1.17 (0.66-2.07) | Reference |
| Adjusted IRD (95% CI), (model 1)^e^ | 15 (−43 to 72) | Reference |
| Mantel-Haenszel pooled estimates of the PS ventile–specific IRRs | 1.16 (0.71-1.90) | Reference |
| Mantel-Haenszel pooled estimates of the PS ventile–specific IRDs | 18.1 (−49.5 to 85.6) | Reference |

CI = confidence interval; COPD = chronic obstructive pulmonary disease; ICS = inhaled glucocorticoids; IR = incidence rate; IRD = incidence rate difference; IRR = incidence rate ratio; LABA = long-acting beta2-agonist; PS = propensity score.

^a^ Monotherapy population 1 was selected using the same approach as in the main analysis (i.e., same inclusion and exclusion criteria) , then restricted to users of olodaterol monotherapy, and finally matched 1:4 with replacement by age, sex, and calendar year with users of “other LABA” monotherapy.

^b^ From a Poisson regression model including exposure, the natural logarithm of the person-years at risk as the offset, and the propensity score quintiles.

^c^ From a Poisson regression model including exposure, the natural logarithm of the person-years at risk as the offset, the propensity score quintiles, transient ischaemic attack, inhaled glucocorticoids (ICS), and number of COPD exacerbations within 90 days.

^d^ Monotherapy population 2 was selected using the same approach as monotherapy population 1, but no matching was performed (i.e., all users of “other LABA” monotherapy that fulfilled the inclusion and exclusion criteria were included).

^e^ From a Poisson regression model including exposure, the natural logarithm of the person-years at risk as the offset, and the propensity score ventiles.

Table S9. Proportion of patients by reason for ending the follow-up after trimming and matching for each outcome and by cohort

| Variable | Olodaterol cohort^a^ | | | | | | Other LABA cohort^b^ | | | | | |
| --- | --- | --- | --- | --- | --- | --- | --- | --- | --- | --- | --- | --- |
|  | AF (%) | SVT (%) | VT (%) | AMI (%) | SACHD (%) | ACM (%) | AF (%) | SVT (%) | VT (%) | AMI (%) | SACHD (%) | ACM (%) |
| Occurrence of the event | 1.7 | 0.1 | 0.2 | 0.4 | 0.3 | NA | 1.4 | 0.1 | 0.2 | 0.3 | 0.3 | NA |
| End of first episode of continuous use^c^ | 61.2 | 62.2 | 62.2 | 62.1 | 62.1 | 62.3 | 62.8 | 63.7 | 63.7 | 63.6 | 63.6 | 63.7 |
| Starting another LABA substance^d^ | 18.8 | 18.9 | 18.9 | 18.9 | 18.9 | 19.0 | 17.8 | 18.0 | 18.0 | 17.9 | 17.9 | 18.0 |
| Death | 5.8 | 6.0 | 5.9 | 6.0 | 6.0 | 6.0 | 3.5 | 3.6 | 3.5 | 3.6 | 3.6 | 3.7 |
| End of study period/emigration | 12.5 | 12.7 | 12.7 | 12.7 | 12.7 | 12.7 | 14.5 | 14.6 | 14.6 | 14.6 | 14.5 | 14.6 |

ACM = all-cause mortality; AF = hospitalisation or hospital outpatient specialist visit for atrial fibrillation or flutter; AMI = hospitalisation for acute myocardial infarction; LABA = long-acting beta2-agonist; SACHD = serious acute coronary heart disease; SVT = hospitalisation or hospital outpatient specialist visit for supraventricular tachycardia (other than atrial fibrillation/flutter); VT = hospitalisation for ventricular tachycardia, including ventricular fibrillation/flutter and cardiac arrest.

Note: The other LABA cohort comprised inhaled long-acting beta2-agonists other than olodaterol.

^a^In all olodaterol outcome-specific cohorts, 30 patients had more than one reason for termination.

^b^In the “other LABA” outcome-specific cohorts, between 70 and 90 patients had more than one reason for termination.

^c^End of first episode of continuous use was defined as 14 days after discontinuation of the last dispensing of olodaterol or LABA.

^d^Starting or addition of a second LABA, irrespective of the concomitant LAMA duration.

Table S10. Standardised Bias for the Comparison of Key Covariates Between New Users of Olodaterol and of Other LABAs in Propensity Score–Trimmed Study Cohort by Propensity Score Quintiles

| **Variables** | | **Untrimmed** | | **Trimmed (overall)** | | **Trimmed (averaged)** | | PSQ1 | | PSQ2 | | PSQ3 | | PSQ4 | | PSQ5 |
| --- | --- | --- | --- | --- | --- | --- | --- | --- | --- | --- | --- | --- | --- | --- | --- | --- |
| Age group (years): 40-60 | | **-0.00309** | | **0.00978** | | **0.002509** | | 0.01789 | | 0.012368 | | 0.021982 | | -0.023952 | | -0.015744 |
| Age group (years): 61-74 | | **0.00316** | | **-0.00251** | | **-0.009670** | | -0.00839 | | -0.000200 | | 0.005120 | | -0.004461 | | -0.040421 |
| Age group (years): 75-84 | | **0.00270** | | **-0.00624** | | **0.000717** | | -0.02747 | | -0.010924 | | -0.057274 | | 0.055600 | | 0.043652 |
| Age group (years): 85 or more | | **-0.00586** | | **0.00306** | | **0.009180** | | 0.03599 | | 0.003551 | | 0.048836 | | -0.054577 | | 0.012095 |
| Female | | **0.00085** | | **-0.00914** | | **-0.006753** | | -0.00917 | | 0.003262 | | 0.008573 | | -0.008754 | | -0.027674 |
| Male | | **-0.00085** | | **0.00914** | | **0.006753** | | 0.00917 | | -0.003262 | | -0.008573 | | 0.008754 | | 0.027674 |
| Calendar year at index: 2014 | | **0.00064** | | **-0.01004** | | **-0.006077** | | 0.03468 | | -0.007687 | | -0.022040 | | -0.009078 | | -0.026260 |
| Calendar year at index: 2015 | | **0.00086** | | **-0.01517** | | **-0.015454** | | -0.00069 | | 0.002652 | | 0.000899 | | -0.020341 | | -0.059787 |
| Calendar year at index: 2016 | | **0.00161** | | **-0.00785** | | **-0.002257** | | -0.04090 | | 0.032346 | | 0.006590 | | 0.014016 | | -0.023341 |
| Calendar year at index: 2017 | | **0.00127** | | **0.00144** | | **-0.000493** | | -0.00805 | | -0.030267 | | 0.002961 | | 0.014825 | | 0.018067 |
| Calendar year at index: 2018-2019 | | **-0.00354** | | **0.01847** | | **0.014278** | | 0.03342 | | 0.001681 | | -0.002010 | | -0.011449 | | 0.049743 |
| Cardiovascular diseases: No | | **0.03269** | | **0.01306** | | **0.000628** | | -0.05764 | | 0.010311 | | 0.003439 | | 0.021372 | | 0.025656 |
| Cardiovascular diseases: Yes | | **-0.03269** | | **-0.01306** | | **-0.000628** | | 0.05764 | | -0.010311 | | -0.003439 | | -0.021372 | | -0.025656 |
| Ischaemic heart disease: No | | **0.01627** | | **0.00941** | | **0.001730** | | -0.04611 | | -0.008457 | | 0.035222 | | 0.006726 | | 0.021271 |
| Ischaemic heart disease: Yes | | **-0.01627** | | **-0.00941** | | **-0.001730** | | 0.04611 | | 0.008457 | | -0.035222 | | -0.006726 | | -0.021271 |
| Angina pectoris: No | | **0.00402** | | **0.00338** | | **0.001657** | | -0.04465 | | 0.005077 | | 0.017896 | | 0.015923 | | 0.014040 |
| Angina pectoris: Yes | | **-0.00402** | | **-0.00338** | | **-0.001657** | | 0.04465 | | -0.005077 | | -0.017896 | | -0.015923 | | -0.014040 |
| Acute myocardial infarction: No | | **0.01026** | | **0.00236** | | **-0.010989** | | -0.01134 | | 0.002868 | | 0.012602 | | -0.033244 | | -0.025832 |
| Acute myocardial infarction: Yes | | **-0.01026** | | **-0.00236** | | **0.010989** | | 0.01134 | | -0.002868 | | -0.012602 | | 0.033244 | | 0.025832 |
| Other acute or subacute ischaemic heart disease: No | | **0.01057** | | **-0.00145** | | **-0.002549** | | -0.02794 | | -0.009770 | | 0.037353 | | -0.020380 | | 0.007994 |
| Other acute or subacute ischaemic heart disease: Yes | | **-0.01057** | | **0.00145** | | **0.002549** | | 0.02794 | | 0.009770 | | -0.037353 | | 0.020380 | | -0.007994 |
| Chronic ischaemic heart disease: No | | **0.01834** | | **0.00813** | | **-0.002465** | | -0.03677 | | 0.000651 | | 0.019614 | | -0.016281 | | 0.020457 |
| Chronic ischaemic heart disease: Yes | | **-0.01834** | | **-0.00813** | | **0.002465** | | 0.03677 | | -0.000651 | | -0.019614 | | 0.016281 | | -0.020457 |
| Coronary reperfusion surgery and procedures: No | | **-0.00377** | | **-0.00451** | | **-0.005433** | | -0.00161 | | 0.023148 | | -0.013779 | | -0.041722 | | 0.006799 |
| Coronary reperfusion surgery and procedures: Yes | | **0.00377** | | **0.00451** | | **0.005433** | | 0.00161 | | -0.023148 | | 0.013779 | | 0.041722 | | -0.006799 |
| Conduction disorders: No | | **0.00791** | | **0.00651** | | **0.000494** | | 0.02472 | | -0.019222 | | -0.003128 | | 0.010231 | | -0.010125 |
| Conduction disorders: Yes | | **-0.00791** | | **-0.00651** | | **-0.000494** | | -0.02472 | | 0.019222 | | 0.003128 | | -0.010231 | | 0.010125 |
| Cardiac arrest: No | | **0.03035** | | **0.01909** | | **0.008398** | | 0.05996 | | 0.018961 | | -0.013435 | | -0.005329 | | -0.018165 |
| Cardiac arrest: Yes | | **-0.03035** | | **-0.01909** | | **-0.008398** | | -0.05996 | | -0.018961 | | 0.013435 | | 0.005329 | | 0.018165 |
| Arrhythmias: No | | **0.03554** | | **0.02401** | | **-0.001414** | | 0.00142 | | 0.019021 | | 0.001883 | | 0.000583 | | -0.029977 |
| Arrhythmias: Yes | | **-0.03554** | | **-0.02401** | | **0.001414** | | -0.00142 | | -0.019021 | | -0.001883 | | -0.000583 | | 0.029977 |
| Paroxysmal tachycardia: No | | **0.02081** | | **0.01724** | | **0.002203** | | 0.00929 | | 0.033821 | | -0.007261 | | 0.024221 | | -0.049050 |
| Paroxysmal tachycardia: Yes | | **-0.02081** | | **-0.01724** | | **-0.002203** | | -0.00929 | | -0.033821 | | 0.007261 | | -0.024221 | | 0.049050 |
| Ventricular tachycardia: No | | **0.03330** | | **0.02287** | | **0.000549** | | 0.02751 | | 0.013114 | | 0.007401 | | -0.003093 | | -0.042186 |
| Ventricular tachycardia: Yes | | **-0.03330** | | **-0.02287** | | **-0.000549** | | -0.02751 | | -0.013114 | | -0.007401 | | 0.003093 | | 0.042186 |
| Supraventricular tachycardia and unspecified: No | | **0.01232** | | **0.00886** | | **0.004167** | | -0.01115 | | 0.037105 | | -0.010537 | | 0.031099 | | -0.025681 |
| Supraventricular tachycardia and unspecified: Yes | | **-0.01232** | | **-0.00886** | | **-0.004167** | | 0.01115 | | -0.037105 | | 0.010537 | | -0.031099 | | 0.025681 |
| Atrial fibrillation and flutter: No | | **0.03685** | | **0.02390** | | **-0.000362** | | -0.00954 | | 0.018934 | | 0.015975 | | -0.009946 | | -0.017227 |
| Atrial fibrillation and flutter: Yes | | **-0.03685** | | **-0.02390** | | **0.000362** | | 0.00954 | | -0.018934 | | -0.015975 | | 0.009946 | | 0.017227 |
| Other cardiac arrhythmias: No | | **0.00666** | | **0.00635** | | **-0.002776** | | 0.06083 | | -0.013555 | | 0.005403 | | 0.002831 | | -0.069384 |
| Other cardiac arrhythmias: Yes | | **-0.00666** | | **-0.00635** | | **0.002776** | | -0.06083 | | 0.013555 | | -0.005403 | | -0.002831 | | 0.069384 |
| Ventricular fibrillation and flutter: No | | **0.00001** | | **0.00295** | | **0.000867** | | 0.03471 | | 0.009521 | | -0.005662 | | -0.000362 | | -0.033871 |
| Ventricular fibrillation and flutter: Yes | | **-0.00001** | | **-0.00295** | | **-0.000867** | | -0.03471 | | -0.009521 | | 0.005662 | | 0.000362 | | 0.033871 |
| Other cardiac arrhythmias (subgroup): No | | **0.00562** | | **0.00524** | | **-0.002848** | | 0.04693 | | -0.012614 | | 0.006997 | | 0.001917 | | -0.057469 |
| Other cardiac arrhythmias (subgroup): Yes | | **-0.00562** | | **-0.00524** | | **0.002848** | | -0.04693 | | 0.012614 | | -0.006997 | | -0.001917 | | 0.057469 |
| Heart failure: No | | **0.04803** | | **0.03133** | | **0.002325** | | 0.00083 | | 0.000685 | | 0.065179 | | -0.005470 | | -0.049604 |
| Heart failure: Yes | | **-0.04803** | | **-0.03133** | | **-0.002325** | | -0.00083 | | -0.000685 | | -0.065179 | | 0.005470 | | 0.049604 |
| Cerebrovascular disease: No | | **0.03158** | | **0.01523** | | **-0.005954** | | -0.01294 | | -0.002009 | | -0.000395 | | 0.008945 | | -0.023372 |
| Cerebrovascular disease: Yes | | **-0.03158** | | **-0.01523** | | **0.005954** | | 0.01294 | | 0.002009 | | 0.000395 | | -0.008945 | | 0.023372 |
| Cerebral haemorrhage (subarachnoid, intracerebral, other non-traumatic): No | | **0.02713** | | **0.01934** | | **0.000246** | | 0.03595 | | -0.012572 | | 0.006671 | | 0.021319 | | -0.050136 |
| Cerebral haemorrhage (subarachnoid, intracerebral, other non-traumatic): Yes | | **-0.02713** | | **-0.01934** | | **-0.000246** | | -0.03595 | | 0.012572 | | -0.006671 | | -0.021319 | | 0.050136 |
| Cerebral infarction and stroke: No | | **0.02993** | | **0.01720** | | **-0.001879** | | -0.02860 | | -0.010689 | | -0.009275 | | 0.031994 | | 0.007178 |
| Cerebral infarction and stroke: Yes | | **-0.02993** | | **-0.01720** | | **0.001879** | | 0.02860 | | 0.010689 | | 0.009275 | | -0.031994 | | -0.007178 |
| Transient ischaemic attack: No | | **0.00395** | | **-0.00104** | | **-0.005658** | | -0.00746 | | 0.014338 | | 0.019381 | | -0.023895 | | -0.030658 |
| Transient ischaemic attack: Yes | | **-0.00395** | | **0.00104** | | **0.005658** | | 0.00746 | | -0.014338 | | -0.019381 | | 0.023895 | | 0.030658 |
| Other cerebrovascular disease and sequelae of cerebrovascular disease: No | | **0.03397** | | **0.02158** | | **-0.000417** | | 0.00207 | | 0.025131 | | -0.004756 | | -0.022409 | | -0.002121 |
| Other cerebrovascular disease and sequelae of cerebrovascular disease: Yes | | **-0.03397** | | **-0.02158** | | **0.000417** | | -0.00207 | | -0.025131 | | 0.004756 | | 0.022409 | | 0.002121 |
| Hypertension and hypertensive heart disease: No | | **-0.03812** | | **-0.02667** | | **-0.004350** | | -0.00232 | | 0.002743 | | 0.025558 | | -0.045366 | | -0.002369 |
| Hypertension and hypertensive heart disease: Yes | | **0.03812** | | **0.02667** | | **0.004350** | | 0.00232 | | -0.002743 | | -0.025558 | | 0.045366 | | 0.002369 |
| Diseases of arteries, arterioles, and capillaries: No | | **0.02030** | | **0.01090** | | **-0.000075** | | 0.00189 | | 0.018326 | | -0.005882 | | -0.018629 | | 0.003919 |
| Diseases of arteries, arterioles, and capillaries: Yes | | **-0.02030** | | **-0.01090** | | **0.000075** | | -0.00189 | | -0.018326 | | 0.005882 | | 0.018629 | | -0.003919 |
| Peripheral arterial revascularisation procedures: No | | **0.01790** | | **0.01471** | | **0.006933** | | -0.01885 | | 0.028763 | | 0.018763 | | -0.014118 | | 0.020109 |
| Peripheral arterial revascularisation procedures: Yes | | **-0.01790** | | **-0.01471** | | **-0.006933** | | 0.01885 | | -0.028763 | | -0.018763 | | 0.014118 | | -0.020109 |
| Other form of heart diseases: No | | **0.03108** | | **0.01388** | | **-0.003274** | | -0.00838 | | -0.013797 | | 0.003739 | | 0.011097 | | -0.009026 |
| Other form of heart diseases: Yes | | **-0.03108** | | **-0.01388** | | **0.003274** | | 0.00838 | | 0.013797 | | -0.003739 | | -0.011097 | | 0.009026 |
| Hyperlipidaemia: No | | **-0.00571** | | **-0.00802** | | **0.002829** | | -0.06667 | | 0.025331 | | 0.011169 | | -0.015860 | | 0.060175 |
| Hyperlipidaemia: Yes | | **0.00571** | | **0.00802** | | **-0.002829** | | 0.06667 | | -0.025331 | | -0.011169 | | 0.015860 | | -0.060175 |
| Diabetes mellitus: No | | **0.00705** | | **-0.00491** | | **-0.008317** | | -0.03032 | | 0.020372 | | -0.002695 | | 0.009217 | | -0.038157 |
| Diabetes mellitus: Yes | | **-0.00705** | | **0.00491** | | **0.008317** | | 0.03032 | | -0.020372 | | 0.002695 | | -0.009217 | | 0.038157 |
| Renal disease: No | | **0.05742** | | **0.03467** | | **0.003705** | | -0.02408 | | 0.014471 | | 0.025144 | | 0.018123 | | -0.015133 |
| Renal disease: Yes | | **-0.05742** | | **-0.03467** | | **-0.003705** | | 0.02408 | | -0.014471 | | -0.025144 | | -0.018123 | | 0.015133 |
| Chronic kidney disease: No | | **0.02711** | | **0.01113** | | **-0.002379** | | 0.00522 | | -0.008705 | | -0.032122 | | 0.003705 | | 0.020010 |
| Chronic kidney disease: Yes | | **-0.02711** | | **-0.01113** | | **0.002379** | | -0.00522 | | 0.008705 | | 0.032122 | | -0.003705 | | -0.020010 |
| Other renal disorders: No | | **0.06017** | | **0.03718** | | **0.005138** | | -0.02718 | | 0.015280 | | 0.030558 | | 0.032913 | | -0.025882 |
| Other renal disorders: Yes | | **-0.06017** | | **-0.03718** | | **-0.005138** | | 0.02718 | | -0.015280 | | -0.030558 | | -0.032913 | | 0.025882 |
| Anaemias: No | | **0.05174** | | **0.02585** | | **-0.001688** | | -0.02646 | | -0.027386 | | 0.010700 | | 0.008897 | | 0.025813 |
| Anaemias: Yes | | **-0.05174** | | **-0.02585** | | **0.001688** | | 0.02646 | | 0.027386 | | -0.010700 | | -0.008897 | | -0.025813 |
| Nutritional anaemias: No | | **0.04609** | | **0.03515** | | **0.005715** | | 0.02609 | | -0.014906 | | -0.021572 | | 0.020398 | | 0.018571 |
| Nutritional anaemias: Yes | | **-0.04609** | | **-0.03515** | | **-0.005715** | | -0.02609 | | 0.014906 | | 0.021572 | | -0.020398 | | -0.018571 |
| Iron deficiency anaemias: No | | **0.04977** | | **0.03775** | | **0.006610** | | 0.01629 | | -0.019986 | | -0.013576 | | 0.017186 | | 0.033139 |
| Iron deficiency anaemias: Yes | | **-0.04977** | | **-0.03775** | | **-0.006610** | | -0.01629 | | 0.019986 | | 0.013576 | | -0.017186 | | -0.033139 |
| Other anaemias: No | | **0.04325** | | **0.01708** | | **-0.003865** | | -0.04713 | | -0.024361 | | 0.010920 | | 0.002780 | | 0.038467 |
| Other anaemias: Yes | | **-0.04325** | | **-0.01708** | | **0.003865** | | 0.04713 | | 0.024361 | | -0.010920 | | -0.002780 | | -0.038467 |
| Peptic ulcer disease: No | | **0.03463** | | **0.01739** | | **-0.000326** | | -0.03899 | | -0.025172 | | 0.018419 | | -0.005853 | | 0.049967 |
| Peptic ulcer disease: Yes | | **-0.03463** | | **-0.01739** | | **0.000326** | | 0.03899 | | 0.025172 | | -0.018419 | | 0.005853 | | -0.049967 |
| Liver disease: No | | **0.01191** | | **0.00585** | | **0.001727** | | -0.01828 | | -0.002832 | | -0.007467 | | 0.010663 | | 0.026549 |
| Liver disease: Yes | | **-0.01191** | | **-0.00585** | | **-0.001727** | | 0.01828 | | 0.002832 | | 0.007467 | | -0.010663 | | -0.026549 |
| Osteoporosis: No | | **0.13466** | | **0.07942** | | **0.003619** | | -0.00971 | | -0.045900 | | 0.019525 | | 0.019901 | | 0.034280 |
| Osteoporosis: Yes | | **-0.13466** | | **-0.07942** | | **-0.003619** | | 0.00971 | | 0.045900 | | -0.019525 | | -0.019901 | | -0.034280 |
| Rheumatoid arthritis and other inflammatory arthropathies: No | | **0.02232** | | **0.01443** | | **0.000415** | | 0.03221 | | 0.016090 | | 0.024405 | | -0.057152 | | -0.013482 |
| Rheumatoid arthritis and other inflammatory arthropathies: Yes | | **-0.02232** | | **-0.01443** | | **-0.000415** | | -0.03221 | | -0.016090 | | -0.024405 | | 0.057152 | | 0.013482 |
| Systemic connective tissue diseases: No | | **-0.00547** | | **-0.00887** | | **-0.002216** | | 0.01356 | | -0.028852 | | -0.019609 | | 0.022827 | | 0.000994 |
| Systemic connective tissue diseases: Yes | | **0.00547** | | **0.00887** | | **0.002216** | | -0.01356 | | 0.028852 | | 0.019609 | | -0.022827 | | -0.000994 |
| Malignancy: No | | **0.03087** | | **0.01613** | | **0.000202** | | 0.03071 | | 0.017229 | | 0.025294 | | -0.052528 | | -0.019699 |
| Malignancy: Yes | | **-0.03087** | | **-0.01613** | | **-0.000202** | | -0.03071 | | -0.017229 | | -0.025294 | | 0.052528 | | 0.019699 |
| Depressive disorders: No | | **0.01970** | | **0.00493** | | **-0.005269** | | -0.01351 | | -0.008268 | | -0.010913 | | -0.000390 | | 0.006739 |
| Depressive disorders: Yes | | **-0.01970** | | **-0.00493** | | **0.005269** | | 0.01351 | | 0.008268 | | 0.010913 | | 0.000390 | | -0.006739 |
| Asthma: No | | **0.02166** | | **0.00186** | | **-0.038146** | | -0.09160 | | -0.014326 | | -0.038055 | | -0.027545 | | -0.019202 |
| Asthma: Yes | | **-0.02166** | | **-0.00186** | | **0.038146** | | 0.09160 | | 0.014326 | | 0.038055 | | 0.027545 | | 0.019202 |
| Respiratory medications: No | | **0.26187** | | **0.19371** | | **0.026802** | | 0.04903 | | 0.066996 | | 0.015240 | | -0.024057 | | N.E. |
| Respiratory medications: Yes | | **-0.26187** | | **-0.19371** | | **-0.026802** | | -0.04903 | | -0.066996 | | -0.015240 | | 0.024057 | | N.E. |
| Inhaled short-acting muscarinic antagonists (SAMAs): No | | **-0.00807** | | **-0.00344** | | **-0.002616** | | 0.00058 | | 0.014395 | | -0.010135 | | -0.003527 | | -0.014393 |
| Inhaled short-acting muscarinic antagonists (SAMAs): Yes | | **0.00807** | | **0.00344** | | **0.002616** | | -0.00058 | | -0.014395 | | 0.010135 | | 0.003527 | | 0.014393 |
| Inhaled long-acting muscarinic antagonists (LAMAs): No | | **0.32689** | | **0.23311** | | **0.014718** | | 0.11374 | | -0.008538 | | 0.009027 | | -0.082511 | | 0.041871 |
| Inhaled long-acting muscarinic antagonists (LAMAs): Yes | | **-0.32689** | | **-0.23311** | | **-0.014718** | | -0.11374 | | 0.008538 | | -0.009027 | | 0.082511 | | -0.041871 |
| Inhaled short-acting beta2-agonists (SABAs): No | | **0.24696** | | **0.17622** | | **0.014587** | | -0.00598 | | 0.048067 | | -0.000700 | | 0.027325 | | 0.004220 |
| Inhaled short-acting beta2-agonists (SABAs): Yes | | **-0.24696** | | **-0.17622** | | **-0.014587** | | 0.00598 | | -0.048067 | | 0.000700 | | -0.027325 | | -0.004220 |
| Inhaled long-acting beta2-agonists (LABAs): No | | **0.29223** | | **0.17804** | | **0.025540** | | 0.02422 | | 0.025160 | | 0.032279 | | 0.022580 | | 0.023461 |
| Inhaled long-acting beta2-agonists (LABAs): Yes | | **-0.29223** | | **-0.17804** | | **-0.025540** | | -0.02422 | | -0.025160 | | -0.032279 | | -0.022580 | | -0.023461 |
| Inhaled glucocorticosteroids (ICS): No | | **0.17203** | | **0.08154** | | **-0.009145** | | -0.08960 | | -0.064255 | | -0.015124 | | 0.074355 | | 0.048897 |
| Inhaled glucocorticosteroids (ICS): Yes | | **-0.17203** | | **-0.08154** | | **0.009145** | | 0.08960 | | 0.064255 | | 0.015124 | | -0.074355 | | -0.048897 |
| Fixed combinations of SABA and SAMA: No | | **0.22035** | | **0.11627** | | **0.008253** | | -0.01472 | | -0.007696 | | 0.018165 | | 0.040298 | | 0.005219 |
| Fixed combinations of SABA and SAMA: Yes | | **-0.22035** | | **-0.11627** | | **-0.008253** | | 0.01472 | | 0.007696 | | -0.018165 | | -0.040298 | | -0.005219 |
| Fixed combinations of LABA and ICS: No | | **0.26876** | | **0.20856** | | **0.013686** | | 0.01615 | | 0.026080 | | -0.010858 | | 0.043119 | | -0.006063 |
| Fixed combinations of LABA and ICS: Yes | | **-0.26876** | | **-0.20856** | | **-0.013686** | | -0.01615 | | -0.026080 | | 0.010858 | | -0.043119 | | 0.006063 |
| Systemic glucocorticosteroids: No | | **0.24925** | | **0.17380** | | **0.015202** | | 0.05905 | | 0.014946 | | 0.034106 | | -0.010225 | | -0.021859 |
| Systemic glucocorticosteroids: Yes | | **-0.24925** | | **-0.17380** | | **-0.015202** | | -0.05905 | | -0.014946 | | -0.034106 | | 0.010225 | | 0.021859 |
| Systemic beta2-agonists: No | | **-0.00517** | | **-0.00138** | | **0.002827** | | -0.00180 | | -0.018636 | | 0.000072 | | 0.034579 | | -0.000077 |
| Systemic beta2-agonists: Yes | | **0.00517** | | **0.00138** | | **-0.002827** | | 0.00180 | | 0.018636 | | -0.000072 | | -0.034579 | | 0.000077 |
| Xanthines and adrenergics: No | | **0.06647** | | **0.03758** | | **0.004197** | | -0.01792 | | 0.008487 | | 0.005219 | | -0.034513 | | 0.059706 |
| Xanthines and adrenergics: Yes | | **-0.06647** | | **-0.03758** | | **-0.004197** | | 0.01792 | | -0.008487 | | -0.005219 | | 0.034513 | | -0.059706 |
| Roflumilast: No | | **0.08038** | | **0.02492** | | **0.005458** | | -0.01619 | | 0.029467 | | -0.025032 | | -0.041875 | | 0.080926 |
| Roflumilast: Yes | | **-0.08038** | | **-0.02492** | | **-0.010871** | | . | | -0.029467 | | 0.025032 | | 0.041875 | | -0.080926 |
| Nasal glucocorticosteroids: No | | **0.00666** | | **0.00124** | | **-0.003322** | | 0.00705 | | 0.016315 | | -0.008692 | | 0.011490 | | -0.042769 |
| Nasal glucocorticosteroids: Yes | | **-0.00666** | | **-0.00124** | | **0.003322** | | -0.00705 | | -0.016315 | | 0.008692 | | -0.011490 | | 0.042769 |
| Leukotriene receptor antagonists: No | | **0.02280** | | **0.01883** | | **-0.000923** | | 0.02577 | | 0.010766 | | -0.008167 | | -0.021794 | | -0.011184 |
| Leukotriene receptor antagonists: Yes | | **-0.02280** | | **-0.01883** | | **0.000923** | | -0.02577 | | -0.010766 | | 0.008167 | | 0.021794 | | 0.011184 |
| Oxygen therapy: No | | **0.19603** | | **0.11283** | | **-0.004200** | | -0.01044 | | 0.039591 | | 0.001058 | | -0.013821 | | -0.037386 |
| Oxygen therapy: Yes | | **-0.19603** | | **-0.11283** | | **0.004200** | | 0.01044 | | -0.039591 | | -0.001058 | | 0.013821 | | 0.037386 |
| Nebuliser therapy: No | | **0.06609** | | **0.02592** | | **-0.000113** | | N.E. | | N.E. | | -0.014942 | | N.E. | | 0.014715 |
| Nebuliser therapy: Yes | | **-0.06609** | | **-0.02592** | | **-0.014715** | | N.E. | | N.E. | | N.E. | | N.E. | | -0.014715 |
| Cardiovascular medications: No | | **0.03119** | | **0.02439** | | **0.007213** | | -0.01868 | | 0.019033 | | 0.034948 | | -0.012217 | | 0.012979 |
| Cardiovascular medications: Yes | | **-0.03119** | | **-0.02439** | | **-0.007213** | | 0.01868 | | -0.019033 | | -0.034948 | | 0.012217 | | -0.012979 |
| Cardiac glycosides and antiarrhythmics, Class I and III: No | | **0.06904** | | **0.03712** | | **-0.001323** | | -0.01277 | | -0.017168 | | 0.007216 | | -0.007973 | | 0.024082 |
| Cardiac glycosides and antiarrhythmics, Class I and III: Yes | | **-0.06904** | | **-0.03712** | | **0.001323** | | 0.01277 | | 0.017168 | | -0.007216 | | 0.007973 | | -0.024082 |
| Vasodilators used in cardiac diseases: No | | **0.02240** | | **0.01723** | | **0.000491** | | -0.01393 | | 0.005081 | | -0.020482 | | 0.040066 | | -0.008281 |
| Vasodilators used in cardiac diseases: Yes | | **-0.02240** | | **-0.01723** | | **-0.000491** | | 0.01393 | | -0.005081 | | 0.020482 | | -0.040066 | | 0.008281 |
| Cardiac stimulants and other cardiac preparations: No | | **0.00995** | | **0.01414** | | **0.018804** | | 0.03308 | | -0.003114 | | -0.023716 | | 0.005900 | | 0.081875 |
| Cardiac stimulants and other cardiac preparations: Yes | | **-0.00995** | | **-0.01414** | | **-0.018804** | | -0.03308 | | 0.003114 | | 0.023716 | | -0.005900 | | -0.081875 |
| Diuretics: No | | **0.10846** | | **0.07139** | | **0.003848** | | -0.02360 | | 0.027075 | | 0.020501 | | 0.021818 | | -0.026551 |
| Diuretics: Yes | | **-0.10846** | | **-0.07139** | | **-0.003848** | | 0.02360 | | -0.027075 | | -0.020501 | | -0.021818 | | 0.026551 |
| Peripheral vasodilators: No | | **-0.00981** | | **-0.00884** | | **-0.014484** | | -0.01448 | | N.E. | | N.E. | | N.E. | | N.E. |
| Peripheral vasodilators: Yes | | **0.00981** | | N.E**.** | | N.E**.** | | N.E. | | N.E. | | N.E. | | N.E. | | N.E. |
| Vasoprotective agents: No | | **-0.01053** | | **-0.00627** | | **0.001870** | | -0.00613 | | -0.018954 | | -0.002598 | | 0.010174 | | 0.026857 |
| Vasoprotective agents: Yes | | **0.01053** | | **0.00627** | | **-0.001870** | | 0.00613 | | 0.018954 | | 0.002598 | | -0.010174 | | -0.026857 |
| Beta blocking agents: No | | **-0.00306** | | **-0.00803** | | **-0.006062** | | 0.02249 | | -0.011631 | | 0.006945 | | -0.043754 | | -0.004356 |
| Beta blocking agents: Yes | | **0.00306** | | **0.00803** | | **0.006062** | | -0.02249 | | 0.011631 | | -0.006945 | | 0.043754 | | 0.004356 |
| Calcium channel blockers: No | | **-0.01039** | | **-0.00569** | | **-0.000556** | | -0.00808 | | 0.021348 | | 0.013559 | | -0.035234 | | 0.005630 |
| Calcium channel blockers: Yes | | **0.01039** | | **0.00569** | | **0.000556** | | 0.00808 | | -0.021348 | | -0.013559 | | 0.035234 | | -0.005630 |
| Antihypertensives: No | | **0.01691** | | **0.01298** | | **0.006707** | | 0.01612 | | -0.017818 | | 0.015770 | | -0.018966 | | 0.038431 |
| Antihypertensives: Yes | | **-0.01691** | | **-0.01298** | | **-0.006707** | | -0.01612 | | 0.017818 | | -0.015770 | | 0.018966 | | -0.038431 |
| Agents acting on the renin-angiotensin system: No | | **-0.04855** | | **-0.03312** | | **0.001588** | | -0.02641 | | 0.001327 | | 0.023435 | | -0.005868 | | 0.015454 |
| Agents acting on the renin-angiotensin system: Yes | | **0.04855** | | **0.03312** | | **-0.001588** | | 0.02641 | | -0.001327 | | -0.023435 | | 0.005868 | | -0.015454 |
| Angiotensin-converting-enzyme inhibitors: No | | **-0.01693** | | **-0.01443** | | **-0.001044** | | -0.01709 | | 0.006414 | | 0.021489 | | -0.009723 | | -0.006308 |
| Angiotensin-converting-enzyme inhibitors: Yes | | **0.01693** | | **0.01443** | | **0.001044** | | 0.01709 | | -0.006414 | | -0.021489 | | 0.009723 | | 0.006308 |
| Angiotensin II receptor antagonists: No | | **-0.04665** | | **-0.02907** | | **0.004418** | | -0.02028 | | -0.011300 | | 0.017375 | | 0.008295 | | 0.028002 |
| Angiotensin II receptor antagonists: Yes | | **0.04665** | | **0.02907** | | **-0.004418** | | 0.02028 | | 0.011300 | | -0.017375 | | -0.008295 | | -0.028002 |
| Renin-inhibitors: No | | **-0.01024** | | **-0.01073** | | **-0.010246** | | -0.00333 | | -0.009104 | | -0.018301 | | N.E. | | N.E. |
| Renin-inhibitors: Yes | | **0.01024** | | **0.01073** | | **0.003332** | | 0.00333 | | N.E. | | N.E. | | N.E. | | N.E. |
| Lipid-modifying agents: No | | **-0.02779** | | **-0.01547** | | **-0.000928** | | -0.03922 | | 0.008203 | | 0.014661 | | -0.018213 | | 0.029927 |
| Lipid-modifying agents: Yes | | **0.02779** | | **0.01547** | | **0.000928** | | 0.03922 | | -0.008203 | | -0.014661 | | 0.018213 | | -0.029927 |
| HMG-CoA reductase inhibitors (statins): No | | **-0.02786** | | **-0.01495** | | **-0.000670** | | -0.03349 | | 0.009308 | | 0.011256 | | -0.017467 | | 0.027037 |
| HMG-CoA reductase inhibitors (statins): Yes | | **0.02786** | | **0.01495** | | **0.000670** | | 0.03349 | | -0.009308 | | -0.011256 | | 0.017467 | | -0.027037 |
| Other lipid-modifying agents: No | | **0.00664** | | **-0.00422** | | **-0.004333** | | -0.05112 | | -0.011987 | | 0.006906 | | 0.004670 | | 0.029867 |
| Other lipid-modifying agents: Yes | | **-0.00664** | | **0.00422** | | **0.004333** | | 0.05112 | | 0.011987 | | -0.006906 | | -0.004670 | | -0.029867 |
| Antithrombotic agents: No | | **0.05432** | | **0.03102** | | **-0.001615** | | -0.01254 | | -0.016680 | | 0.025963 | | 0.000037 | | -0.004858 |
| Antithrombotic agents: Yes | | **-0.05432** | | **-0.03102** | | **0.001615** | | 0.01254 | | 0.016680 | | -0.025963 | | -0.000037 | | 0.004858 |
| Platelet aggregation inhibitors: No | | **0.03917** | | **0.02358** | | **0.001461** | | -0.00099 | | -0.012637 | | 0.037195 | | -0.022172 | | 0.005913 |
| Platelet aggregation inhibitors: Yes | | **-0.03917** | | **-0.02358** | | **-0.001461** | | 0.00099 | | 0.012637 | | -0.037195 | | 0.022172 | | -0.005913 |
| Systemic antibacterials: No | | **0.19348** | | **0.13338** | | **0.005627** | | 0.02266 | | -0.020275 | | 0.039875 | | 0.002291 | | -0.016412 |
| Systemic antibacterials: Yes | | **-0.19348** | | **-0.13338** | | **-0.005627** | | -0.02266 | | 0.020275 | | -0.039875 | | -0.002291 | | 0.016412 |
| Iron preparations: No | | **0.03692** | | **0.02113** | | **0.002552** | | 0.01856 | | -0.013522 | | 0.035441 | | 0.010069 | | -0.037790 |
| Iron preparations: Yes | | **-0.03692** | | **-0.02113** | | **-0.002552** | | -0.01856 | | 0.013522 | | -0.035441 | | -0.010069 | | 0.037790 |
| Proton pump inhibitors: No | | **0.05645** | | **0.03296** | | **0.000827** | | -0.02683 | | -0.020858 | | 0.013725 | | 0.020610 | | 0.017490 |
| Proton pump inhibitors: Yes | | **-0.05645** | | **-0.03296** | | **-0.000827** | | 0.02683 | | 0.020858 | | -0.013725 | | -0.020610 | | -0.017490 |
| Drugs used in diabetes: No | | **0.00275** | | **-0.00814** | | **-0.007429** | | -0.00163 | | 0.000651 | | -0.014176 | | -0.001673 | | -0.020317 |
| Drugs used in diabetes: Yes | | **-0.00275** | | **0.00814** | | **0.007429** | | 0.00163 | | -0.000651 | | 0.014176 | | 0.001673 | | 0.020317 |
| Insulins: No | | **0.02010** | | **0.01098** | | **0.001845** | | 0.01230 | | -0.028290 | | 0.029665 | | 0.008733 | | -0.013184 |
| Insulins: Yes | | **-0.02010** | | **-0.01098** | | **-0.001845** | | -0.01230 | | 0.028290 | | -0.029665 | | -0.008733 | | 0.013184 |
| Blood glucose–lowering drugs: No | | **-0.00058** | | **-0.01015** | | **-0.009060** | | 0.00288 | | 0.009460 | | -0.011393 | | -0.013688 | | -0.032565 |
| Blood glucose–lowering drugs: Yes | | **0.00058** | | **0.01015** | | **0.009060** | | -0.00288 | | -0.009460 | | 0.011393 | | 0.013688 | | 0.032565 |
| Drugs for musculoskeletal system: No | | **-0.01452** | | **-0.00672** | | **-0.005230** | | 0.05662 | | -0.008164 | | -0.000278 | | -0.041891 | | -0.032435 |
| Drugs for musculoskeletal system: Yes | | **0.01452** | | **0.00672** | | **0.005230** | | -0.05662 | | 0.008164 | | 0.000278 | | 0.041891 | | 0.032435 |
| Anti-inflammatory and antirheumatic products, non-steroids (non-steroidal anti-inflammatory drugs): | | **-0.01742** | | **-0.00885** | | **-0.004731** | | 0.05129 | | -0.007991 | | -0.003348 | | -0.042213 | | -0.021395 |
| Anti-inflammatory and antirheumatic products, non-steroids (non-steroidal anti-inflammatory drugs): | | **0.01742** | | **0.00885** | | **0.004731** | | -0.05129 | | 0.007991 | | 0.003348 | | 0.042213 | | 0.021395 |
| Acetylsalicylic acid (other analgesics and antipyretics): No | | **0.01456** | | **0.01256** | | **0.002252** | | 0.02902 | | -0.001334 | | 0.003092 | | -0.001130 | | -0.018393 |
| Acetylsalicylic acid (other analgesics and antipyretics): Yes | | **-0.01456** | | **-0.01256** | | **-0.002252** | | -0.02902 | | 0.001334 | | -0.003092 | | 0.001130 | | 0.018393 |
| Antidepressants: No | | **0.06752** | | **0.04061** | | **0.003646** | | 0.02614 | | -0.024461 | | 0.019823 | | -0.012971 | | 0.009695 |
| Antidepressants: Yes | | **-0.06752** | | **-0.04061** | | **-0.003646** | | -0.02614 | | 0.024461 | | -0.019823 | | 0.012971 | | -0.009695 |
| Selective serotonin reuptake inhibitors: No | | **0.04426** | | **0.02754** | | **0.000350** | | 0.02576 | | -0.015482 | | 0.024159 | | -0.018407 | | -0.014279 |
| Selective serotonin reuptake inhibitors: Yes | | **-0.04426** | | **-0.02754** | | **-0.000350** | | -0.02576 | | 0.015482 | | -0.024159 | | 0.018407 | | 0.014279 |
| Antineoplastic agents: No | | **0.01829** | | **0.01066** | | **-0.002995** | | N.E. | | -0.028800 | | -0.004512 | | -0.001641 | | 0.022971 |
| Antineoplastic agents: Yes | | **-0.01829** | | **-0.01066** | | **-0.005606** | | N.E. | | N.E. | | 0.004512 | | 0.001641 | | -0.022971 |
| Immunosuppressants: No | | **-0.00747** | | **-0.00552** | | **0.000603** | | -0.00564 | | 0.024211 | | 0.005087 | | 0.031537 | | -0.052176 |
| Immunosuppressants: Yes | | **0.00747** | | **0.00552** | | **-0.000603** | | 0.00564 | | -0.024211 | | -0.005087 | | -0.031537 | | 0.052176 |
| Antivirals for systemic use: No | | **0.02735** | | **0.01135** | | **-0.001978** | | -0.00539 | | -0.003948 | | -0.027699 | | 0.004784 | | 0.022361 |
| Antivirals for systemic use: Yes | | **-0.02735** | | **-0.01135** | | **0.001978** | | 0.00539 | | 0.003948 | | 0.027699 | | -0.004784 | | -0.022361 |
| Hormone-replacement therapy: Estrogens, progestogens, progestogens and estrogens in combination: No | | **-0.03301** | | **-0.01812** | | **-0.002925** | | -0.02372 | | -0.013057 | | -0.013807 | | 0.043924 | | -0.007964 |
| Hormone-replacement therapy: Estrogens, progestogens, progestogens and estrogens in combination: Yes | | **0.03301** | | **0.01812** | | **0.002925** | | 0.02372 | | 0.013057 | | 0.013807 | | -0.043924 | | 0.007964 |
| Drugs used in nicotine dependence: No | | **0.06915** | | **0.03572** | | **-0.000458** | | -0.00798 | | -0.023921 | | 0.026563 | | -0.013879 | | 0.016927 |
| Drugs used in nicotine dependence: Yes | | **-0.06915** | | **-0.03572** | | **0.000458** | | 0.00798 | | 0.023921 | | -0.026563 | | 0.013879 | | -0.016927 |
| COPD severity: mild | | **0.24293** | | **0.17878** | | **0.023651** | | 0.07614 | | 0.008355 | | 0.018116 | | -0.008010 | | N.E. |
| COPD severity: moderate | | **0.16868** | | **0.13938** | | **-0.006937** | | 0.01830 | | 0.023876 | | -0.026158 | | -0.026001 | | -0.024702 |
| COPD severity: severe | | **-0.16156** | | **-0.13790** | | **-0.018471** | | -0.09344 | | -0.006729 | | -0.003162 | | 0.026631 | | -0.015650 |
| COPD severity: very severe | | **-0.22846** | | **-0.13834** | | **0.001154** | | -0.01543 | | -0.024255 | | 0.026611 | | -0.009019 | | 0.027863 |
| CCI score: 1 | **0.03800** | | **0.01065** | | **-0.01121** | | -0.01871 | | 0.01721 | | -0.01187 | | -0.02948 | | -0.01320 | |
| CCI score: 2 | **0.02487** | | **0.02445** | | **0.01527** | | 0.05218 | | 0.01768 | | 0.01969 | | 0.00349 | | -0.01667 | |
| CCI score: 3+ | **-0.05632** | | **-0.02990** | | **-0.00218** | | -0.02506 | | -0.03064 | | -0.00494 | | 0.02451 | | 0.02523 | |
| Hip fracture: No | **0.02407** | | **0.01732** | | **0.00942** | | -0.01398 | | 0.02268 | | -0.00059 | | -0.03251 | | 0.07150 | |
| Hip fracture: Yes | **-0.02407** | | **-0.01732** | | **-0.00942** | | 0.01398 | | -0.02268 | | 0.00059 | | 0.03251 | | -0.07150 | |
| Lung cancer: No | **0.07443** | | **0.07799** | | **0.06649** | | 0.10466 | | 0.10614 | | 0.08896 | | -0.01474 | | 0.04744 | |
| Lung cancer: Yes | **-0.07443** | | **-0.07799** | | **-0.06649** | | -0.10466 | | -0.10614 | | -0.08896 | | 0.01474 | | -0.04744 | |
| Other markers of bad fall: No | **-0.00202** | | **-0.00383** | | **-0.00216** | | -0.02515 | | -0.01936 | | 0.01800 | | 0.04327 | | -0.02757 | |
| Other markers of bad fall: Yes | **0.00202** | | **0.00383** | | **0.00216** | | 0.02515 | | 0.01936 | | -0.01800 | | -0.04327 | | 0.02757 | |
| Metastatic cancer: No | **0.03014** | | **0.02834** | | **0.01779** | | 0.05089 | | 0.01783 | | 0.04192 | | -0.03789 | | 0.01617 | |
| Metastatic cancer: Yes | **-0.03014** | | **-0.02834** | | **-0.01779** | | -0.05089 | | -0.01783 | | -0.04192 | | 0.03789 | | -0.01617 | |
| Pulmonary cachexia: No | **0.02901** | | **0.02731** | | **0.01561** | | 0.01179 | | 0.03747 | | 0.00131 | | 0.02246 | | 0.00502 | |
| Pulmonary cachexia: Yes | **-0.02901** | | **-0.02731** | | **-0.01561** | | -0.01179 | | -0.03747 | | -0.00131 | | -0.02246 | | -0.00502 | |
| Right-sided heart failure: No | **0.06036** | | **0.05653** | | **0.05429** | | 0.04625 | | 0.03707 | | 0.02963 | | 0.09057 | | 0.06795 | |
| Right-sided heart failure: Yes | **-0.06036** | | **-0.05653** | | **-0.05429** | | -0.04625 | | -0.03707 | | -0.02963 | | -0.09057 | | -0.06795 | |
| Number of hospitalisations within 180 days: 0 | **0.31486** | | **0.26255** | | **0.16847** | | 0.17832 | | 0.16566 | | 0.17291 | | 0.19150 | | 0.13394 | |
| Number of hospitalisations within 180 days: 1 | **-0.12594** | | **-0.10726** | | **-0.05424** | | -0.14731 | | -0.07508 | | -0.02926 | | -0.00630 | | -0.01325 | |
| Number of hospitalisations within 180 days: 2 | **-0.17994** | | **-0.15055** | | **-0.10389** | | -0.03982 | | -0.09805 | | -0.13168 | | -0.19893 | | -0.05097 | |
| Number of hospitalisations within 180 days: 3-4 | **-0.20227** | | **-0.16507** | | **-0.11246** | | -0.09302 | | -0.09948 | | -0.11985 | | -0.13406 | | -0.11587 | |
| Number of hospitalisations within 180 days: 5+ | **-0.14172** | | **-0.11276** | | **-0.06669** | | -0.00179 | | -0.03755 | | -0.11061 | | -0.07208 | | -0.11142 | |
| Number of hospitalisations within 365 days: 0 | **0.26324** | | **0.20897** | | **0.10705** | | 0.11943 | | 0.07633 | | 0.10516 | | 0.11368 | | 0.12065 | |
| Number of hospitalisations within 365 days: 1 | **-0.04084** | | **-0.03049** | | **0.00658** | | -0.05595 | | 0.01888 | | 0.00621 | | 0.05070 | | 0.01304 | |
| Number of hospitalisations within 365 days: 2 | **-0.11221** | | **-0.08942** | | **-0.04395** | | -0.03836 | | -0.04690 | | -0.03181 | | -0.07509 | | -0.02760 | |
| Number of hospitalisations within 365 days: 3-4 | **-0.18024** | | **-0.14732** | | **-0.08784** | | -0.09498 | | -0.06431 | | -0.08365 | | -0.09934 | | -0.09693 | |
| Number of hospitalisations within 365 days: 5+ | **-0.19544** | | **-0.14812** | | **-0.08912** | | -0.04211 | | -0.08991 | | -0.10795 | | -0.11424 | | -0.09140 | |
| Number of hospitalisations with COPD within 90 days: 0 | **0.39434** | | **0.33742** | | **0.24138** | | 0.27290 | | 0.23364 | | 0.24227 | | 0.22676 | | 0.23133 | |
| Number of hospitalisations with COPD within 90 days: 1 | **-0.28352** | | **-0.24865** | | **-0.17891** | | -0.24789 | | -0.20056 | | -0.16751 | | -0.12744 | | -0.15114 | |
| Number of hospitalisations with COPD within 90 days: 2+ | **-0.29976** | | **-0.24928** | | **-0.16489** | | -0.11385 | | -0.10687 | | -0.20475 | | -0.23634 | | -0.16261 | |
| Number of hospitalisations with COPD within 180 days: 0 | **0.37618** | | **0.31544** | | **0.19933** | | 0.22414 | | 0.21284 | | 0.20820 | | 0.16573 | | 0.18575 | |
| Number of hospitalisations with COPD within 180 days: 1 | **-0.21919** | | **-0.19139** | | **-0.11450** | | -0.19562 | | -0.17077 | | -0.10450 | | -0.03002 | | -0.07161 | |
| Number of hospitalisations with COPD within 180 days: 2+ | **-0.34851** | | **-0.28227** | | **-0.17410** | | -0.10718 | | -0.11200 | | -0.22450 | | -0.24797 | | -0.17883 | |
| Number of COPD exacerbations within 90 days: 0 | **0.31003** | | **0.23958** | | **0.10119** | | 0.15367 | | 0.08830 | | 0.11637 | | 0.05846 | | 0.08915 | |
| Number of COPD exacerbations within 90 days: 1 | **-0.07791** | | **-0.05338** | | **-0.00516** | | -0.09597 | | 0.00595 | | -0.02404 | | 0.07561 | | 0.01266 | |
| Number of COPD exacerbations within 90 days: 2 | **-0.19892** | | **-0.16488** | | **-0.07097** | | -0.10421 | | -0.09726 | | -0.05355 | | -0.06950 | | -0.03030 | |
| Number of COPD exacerbations within 90 days: 3+ | **-0.27892** | | **-0.21322** | | **-0.10594** | | -0.06741 | | -0.09112 | | -0.12505 | | -0.14200 | | -0.10412 | |
| Number of COPD exacerbations within 180 days: 0 | **0.28956** | | **0.21992** | | **0.06023** | | 0.13172 | | 0.03613 | | 0.09440 | | 0.00939 | | 0.02950 | |
| Number of COPD exacerbations within 180 days: 1 | **-0.00191** | | **0.00308** | | **0.01069** | | -0.08696 | | 0.02158 | | -0.01185 | | 0.07792 | | 0.05277 | |
| Number of COPD exacerbations within 180 days: 2 | **-0.09194** | | **-0.06935** | | **0.01564** | | -0.03614 | | 0.00996 | | -0.00032 | | 0.05300 | | 0.05172 | |
| Number of COPD exacerbations within 180 days: 3+ | **-0.35295** | | **-0.27119** | | **-0.11871** | | -0.08970 | | -0.11219 | | -0.11205 | | -0.15470 | | -0.12488 | |

CCI = Charlson Comorbidity Index; COPD = chronic obstructive pulmonary disease; HMG-CoA = hydroxymethylglutaryl-coenzyme A; ICS = inhaled glucocorticosteroid; LABA = long-acting beta2-agonist; LAMA = long-acting muscarinic antagonist; NE = not estimable; PSQ = propensity score quintile; SABA = short-acting beta2-agonist; SAMA = short-acting muscarinic antagonist.

Note on colour codes:

ORANGE: Cells in orange indicate standardised bias ≥ 0.1 and < 0.2.

YELLOW: Cells in yellow indicate standardised bias ≥ 0.2.

Table S11. Patient Demographics, Clinical Characteristics, and Medications at the Index Date in Each Study Cohort After Trimming and Matching Stratified by PS Quintile Groups, Overall Cohort

|  | **Cohort** | | | | | | | | | | | | | | |
| --- | --- | --- | --- | --- | --- | --- | --- | --- | --- | --- | --- | --- | --- | --- | --- |
|  | **Olodaterol cohort** | | | | | | | **Other LABA cohort** | | | | | | | |
|  | **Overall** | | **PS quintile** | | | | | **Overall** | | | **PS quintile** | | | | |
|  |  |  | **1** | **2** | **3** | **4** | **5** |  |  |  | **1** | **2** | **3** | **4** | **5** |
|  | **N** | **%** | **%** | **%** | **%** | **%** | **%** | **N** | | **%** | **%** | **%** | **%** | **%** | **%** |
| Total | **14,239** | **100** | 100 | 100 | 100 | 100 | 100 | **51,167** | | **100** | 100 | 100 | 100 | 100 | 100 |
| **Age (years), N** | **14,239** |  | 2,847 | 2,848 | 2,848 | 2,849 | 2,847 | **51,167** | |  | 19,071 | 12,066 | 8,960 | 6,695 | 4,375 |
| Mean (SD) | **72.7  (10.0)** |  | 72.4 (9.6) | 72.5 (9.9) | 73.1 (10.3) | 72.7 (10.0) | 73.0 (9.9) | **72.7  (10.0)** | |  | 72.4 (10.0) | 72.5 (10.0) | 73.1 (10.2) | 72.8 (9.5) | 73.4 (9.8) |
| Median (Q1, Q3) | **73  (66, 80)** |  | 73  (66, 39) | 73  (66, 80) | 74  (66, 81) | 73  (66, 80) | 73  (67, 80) | **73  (66, 80)** | |  | 73  (66, 88) | 73  (66, 80) | 74  (66, 81) | 73  (67, 80) | 74  (67, 81) |
| Min, Max (rounded to 10 years) | **40, 100** |  | 40, 100 | 40, 100 | 40, 100 | 40, 100 | 40, 100 | **40, 100** | |  | 40, 100 | 40, 100 | 40, 100 | 40, 100 | 40, 100 |
| **Age group (years)** | **1,700** | **11.9** | 11.8 | 12.5 | 11.4 | 12.2 | 11.7 | **6,273** | | **12.3** | 12.4 | 12.9 | 12.2 | 11.5 | 11.2 |
| 40-60 |  |  |  |  |  |  |  |  |  |  |  |  |  |  |  |
| 61-74 | **6,089** | **42.8** | 43.9 | 42.9 | 40.7 | 43.2 | 43.1 | **21,817** | | **42.6** | 43.5 | 42.9 | 41 | 43 | 41.1 |
| 75-84 | **4,819** | **33.8** | 34.5 | 33.8 | 35.2 | 32.4 | 33.2 | **17,166** | | **33.5** | 33.2 | 33.3 | 32.5 | 35.1 | 35.3 |
| 85+ | **1,631** | **11.5** | 9.7 | 10.8 | 12.6 | 12.1 | 12 | **5,911** | | **11.6** | 10.8 | 10.9 | 14.4 | 10.4 | 12.4 |
| **Gender** | **7,649** | **53.7** | 53.1 | 53.6 | 52.6 | 55.3 | 54 | **27,253** | | **53.3** | 52.7 | 53.8 | 53 | 54.8 | 52.6 |
| Female |  |  |  |  |  |  |  |  |  |  |  |  |  |  |  |
| Male | **6,590** | **46.3** | 46.9 | 46.4 | 47.4 | 44.7 | 46 | **23,914** | | **46.7** | 47.3 | 46.2 | 47 | 45.2 | 47.4 |
| **Calendar year at index date** |  |  |  |  |  |  |  |  | |  |  |  |  |  |  |
| 2014 | **390** | **2.7** | 2.1 | 2.3 | 2.8 | 3.3 | 3.2 | **1,320** | | **2.6** | 2.6 | 2.2 | 2.5 | 3.1 | 2.8 |
| 2015 | **1,407** | **9.9** | 9.8 | 8.7 | 9.1 | 10.7 | 11.1 | **4,829** | | **9.4** | 9.8 | 8.8 | 9.1 | 10.1 | 9.3 |
| 2016 | **3,344** | **23.5** | 24.6 | 21.6 | 22 | 24.2 | 25 | **11,847** | | **23.2** | 22.9 | 23 | 22.3 | 24.8 | 24 |
| 2017 | **4,561** | **32** | 32.9 | 32.7 | 32.6 | 31.2 | 30.8 | **16,424** | | **32.1** | 32.5 | 31.3 | 32.7 | 31.9 | 31.7 |
| 2018 | **4,197** | **29.5** | 28.8 | 32.4 | 30.9 | 27.8 | 27.5 | **15,573** | | **30.4** | 30.1 | 31.9 | 31.2 | 27.8 | 30.2 |
| 2019 | **340** | **2.4** | 1.9 | 2.2 | 2.7 | 2.8 | 2.4 | **1,174** | | **2.3** | 2.1 | 2.8 | 2.3 | 2.3 | 2 |
| Cardiovascular diseases | **10,500** | **73.7** | 69.3 | 73.1 | 75.3 | 74.9 | 76.2 | **37,435** | | **73.2** | 71.9 | 72.6 | 75.2 | 74 | 75 |
| Ischaemic heart disease | **4,138** | **29.1** | 26.5 | 27.1 | 30.2 | 30.2 | 31.4 | **14,652** | | **28.6** | 28.6 | 27.4 | 28.6 | 29.8 | 30.4 |
| Angina pectoris | **3,072** | **21.6** | 19.7 | 20.9 | 22.7 | 22.3 | 22.3 | **10,968** | | **21.4** | 21.5 | 20.7 | 21.9 | 21.6 | 21.8 |
| Acute myocardial infarction | **1,479** | **10.4** | 9.5 | 9.8 | 10.6 | 10.4 | 11.6 | **5,278** | | **10.3** | 9.8 | 9.7 | 10.3 | 11.4 | 12.5 |
| Other acute or subacute ischaemic heart disease | **170** | **1.2** | 0.9 | 1.2 | 1.1 | 1.4 | 1.4 | **619** | | **1.2** | 1.2 | 1.3 | 0.8 | 1.7 | 1.3 |
| Chronic ischaemic heart disease | **2,661** | **18.7** | 16.6 | 17.2 | 19.7 | 19 | 20.9 | **9,401** | | **18.4** | 18 | 17.2 | 18.9 | 19.7 | 20.1 |
| Coronary reperfusion surgery and procedures | **1,558** | **10.9** | 11.3 | 10.9 | 10.8 | 10.4 | 11.3 | **5,671** | | **11.1** | 11.3 | 10.2 | 11.3 | 11.7 | 11.1 |
| Conduction disorders | **473** | **3.3** | 3.3 | 2.7 | 3.8 | 3.5 | 3.3 | **1,641** | | **3.2** | 2.9 | 3 | 3.8 | 3.4 | 3.5 |
| Cardiac arrest | **92** | **0.6** | 0.5 | 0.5 | 0.6 | 0.9 | 0.6 | **261** | | **0.5** | 0.2 | 0.4 | 0.7 | 1 | 0.8 |
| Arrhythmias | **3,285** | **23.1** | 20.4 | 21.5 | 23.8 | 23.9 | 25.6 | **11,295** | | **22.1** | 20.4 | 20.8 | 23.8 | 23.9 | 27 |
| Paroxysmal tachycardia | **673** | **4.7** | 4.2 | 4.4 | 4.4 | 5.3 | 5.3 | **2,238** | | **4.4** | 4 | 3.8 | 4.6 | 4.7 | 6.5 |
| Ventricular tachycardia | **182** | **1.3** | 0.8 | 1 | 1.2 | 1.8 | 1.6 | **535** | | **1** | 0.6 | 0.9 | 1.1 | 1.9 | 2.2 |
| Supraventricular tachycardia and unspecified | **437** | **3.1** | 2.9 | 2.9 | 2.8 | 3.2 | 3.6 | **1,494** | | **2.9** | 3.1 | 2.3 | 3 | 2.7 | 4.1 |
| Atrial fibrillation and flutter | **2,740** | **19.2** | 16.4 | 18 | 20.1 | 19.7 | 22 | **9,373** | | **18.3** | 16.8 | 17.3 | 19.5 | 20.1 | 22.7 |
| Other cardiac arrhythmias | **773** | **5.4** | 6.3 | 4.7 | 5.5 | 5.8 | 4.9 | **2,705** | | **5.3** | 5 | 5 | 5.4 | 5.7 | 6.6 |
| Ventricular fibrillation and flutter | **56** | **0.4** | 0.6 | 0.4 | 0.3 | 0.4 | 0.3 | **192** | | **0.4** | 0.4 | 0.3 | 0.3 | 0.4 | 0.6 |
| Other cardiac arrhythmias (subgroup) | **727** | **5.1** | 5.8 | 4.4 | 5.2 | 5.4 | 4.7 | **2,554** | | **5** | 4.8 | 4.7 | 5.1 | 5.4 | 6.1 |
| Heart failure | **2,236** | **15.7** | 12.3 | 14.7 | 17.8 | 17.2 | 16.5 | **7,469** | | **14.6** | 12.3 | 14.7 | 15.4 | 17.4 | 18.5 |
| Cerebrovascular disease | **2,456** | **17.2** | 14.4 | 16.5 | 17.9 | 18.8 | 18.6 | **8,535** | | **16.7** | 14.9 | 16.5 | 18 | 18.4 | 19.5 |
| Cerebral haemorrhage (subarachnoid, intracerebral, other non-traumatic) | **234** | **1.6** | 1.3 | 1.2 | 1.4 | 2.5 | 1.8 | **724** | | **1.4** | 1 | 1.3 | 1.3 | 2.2 | 2.6 |
| Cerebral infarction and stroke | **1,543** | **10.8** | 7.9 | 10 | 11.8 | 12.3 | 12.1 | **5,277** | | **10.3** | 8.7 | 10.4 | 12.1 | 11.3 | 11.9 |
| Transient ischaemic attack | **815** | **5.7** | 5.6 | 5.6 | 5.8 | 6 | 5.6 | **2,941** | | **5.7** | 5.8 | 5.3 | 5.4 | 6.6 | 6.3 |
| Other cerebrovascular disease and sequelae of cerebrovascular disease | **1,277** | **9** | 7 | 8.6 | 9.9 | 9.2 | 10.2 | **4,283** | | **8.4** | 6.9 | 7.9 | 10 | 9.8 | 10.3 |
| Hypertension and hypertensive heart disease | **5,204** | **36.5** | 40.3 | 36.9 | 38.4 | 34.5 | 32.7 | **19,362** | | **37.8** | 40.4 | 36.8 | 37.1 | 36.7 | 32.8 |
| Diseases of arteries, arterioles, and capillaries | **2,779** | **19.5** | 18.2 | 19.5 | 19.7 | 19.7 | 20.5 | **9,767** | | **19.1** | 18.1 | 18.8 | 19.9 | 20.5 | 20.3 |
| Peripheral arterial revascularisation procedures | **1,154** | **8.1** | 6.8 | 8.1 | 8.6 | 8.2 | 8.8 | **3,946** | | **7.7** | 7.3 | 7.3 | 8.1 | 8.6 | 8.2 |
| Other form of heart diseases | **3,988** | **28** | 25 | 27.7 | 27.6 | 30.1 | 29.6 | **14,014** | | **27.4** | 25.4 | 28.4 | 27.5 | 29.6 | 30 |
| Hyperlipidaemia | **2,591** | **18.2** | 16.8 | 19.1 | 18.4 | 18.1 | 18.6 | **9,470** | | **18.5** | 19.4 | 18.1 | 17.9 | 18.7 | 16.4 |
| Diabetes mellitus | **2,532** | **17.8** | 16.5 | 19 | 18.2 | 17.9 | 17.4 | **9,195** | | **18** | 17.6 | 18.2 | 18.3 | 17.6 | 18.9 |
| Renal disease | **4,139** | **29.1** | 23.3 | 28 | 31.4 | 30.6 | 32 | **14,081** | | **27.5** | 24.4 | 27.4 | 30.2 | 29.8 | 32.7 |
| Chronic kidney disease | **684** | **4.8** | 3.6 | 4.8 | 5 | 5.1 | 5.6 | **2,339** | | **4.6** | 3.5 | 5 | 5.8 | 5 | 5.1 |
| Other renal disorders | **3,640** | **25.6** | 19.8 | 24.6 | 27.8 | 27.4 | 28.2 | **12,268** | | **24** | 20.9 | 23.9 | 26.5 | 26 | 29.3 |
| Anaemias | **1,619** | **11.4** | 7.9 | 9.4 | 12.6 | 12.9 | 13.9 | **5,411** | | **10.6** | 8.7 | 10.3 | 12.3 | 12.6 | 13.1 |
| Nutritional anaemias | **659** | **4.6** | 2.9 | 3.7 | 4.8 | 5.5 | 6.3 | **2,018** | | **3.9** | 2.5 | 4 | 5.3 | 5.1 | 5.9 |
| Iron deficiency anaemias | **563** | **4** | 2.1 | 2.9 | 4.1 | 4.9 | 5.7 | **1,679** | | **3.3** | 1.9 | 3.3 | 4.4 | 4.6 | 4.9 |
| Other anaemias | **1,324** | **9.3** | 6.3 | 7.8 | 10.5 | 10.7 | 11.3 | **4,510** | | **8.8** | 7.5 | 8.4 | 10.1 | 10.6 | 10.1 |
| Peptic ulcer disease | **1,313** | **9.2** | 6.6 | 7.5 | 10.4 | 10.4 | 11.2 | **4,467** | | **8.7** | 7.7 | 8.2 | 9.8 | 10.6 | 9.7 |
| Liver disease | **562** | **3.9** | 3.3 | 3.5 | 4.2 | 4.2 | 4.5 | **1,962** | | **3.8** | 3.7 | 3.6 | 4.3 | 4 | 4 |
| Osteoporosis | **3,374** | **23.7** | 14.1 | 18.3 | 23.7 | 28.1 | 34.3 | **10,484** | | **20.5** | 14.5 | 20.1 | 22.8 | 27.2 | 32.7 |
| Rheumatoid arthritis and other inflammatory arthropathies | **1,369** | **9.6** | 9.3 | 9.4 | 9.9 | 8.8 | 10.6 | **4,706** | | **9.2** | 8.4 | 9 | 9.2 | 10.6 | 11 |
| Systemic connective tissue diseases | **627** | **4.4** | 5.1 | 4.1 | 4.3 | 4.4 | 4.2 | **2,348** | | **4.6** | 4.8 | 4.7 | 4.7 | 3.9 | 4.2 |
| Malignancy | **3,471** | **24.4** | 23 | 24.9 | 25.9 | 23.2 | 24.8 | **12,122** | | **23.7** | 21.7 | 24.2 | 24.9 | 25.5 | 25.6 |
| Depressive disorders | **805** | **5.7** | 4.6 | 5 | 6.6 | 5.8 | 6.3 | **2,835** | | **5.5** | 4.9 | 5.2 | 6.8 | 5.8 | 6.1 |
| Pregnancy (at the index date) | **0** | **0** | 0 | 0 | 0 | 0 | 0 | **0** | | **0** | 0 | 0 | 0 | 0 | 0 |
| Asthma | **2,426** | **17** | 11.5 | 14.3 | 17.1 | 19.4 | 22.8 | **8,682** | | **17** | 14.8 | 14.8 | 18.6 | 20.5 | 23.6 |
| Respiratory medications | **13,109** | **92.1** | 69.4 | 92 | 99 | 99.9 | 100 | **43,585** | | **85.2** | 67.1 | 90 | 98.8 | 99.9 | 100 |
| Inhaled short-acting muscarinic antagonists (SAMAs) | **48** | **0.3** | 0.4 | 0.4 | 0.2 | 0.5 | 0.1 | **183** | | **0.4** | 0.4 | 0.3 | 0.3 | 0.6 | 0.2 |
| Inhaled long-acting muscarinic antagonists (LAMAs) | **7,219** | **50.7** | 21.5 | 34.4 | 50.1 | 66.8 | 80.6 | **20,115** | | **39.3** | 17.2 | 34.8 | 49.7 | 70.6 | 78.9 |
| Inhaled short-acting beta2-agonists (SABAs) | **8,591** | **60.3** | 32.9 | 52.7 | 63.9 | 71.8 | 80.3 | **26,365** | | **51.5** | 33.2 | 50.3 | 64 | 70.6 | 80.1 |
| Inhaled long-acting beta2-agonists (LABAs) | **1,346** | **9.5** | 0.5 | 2.9 | 7.4 | 13.5 | 23 | **2,774** | | **5.4** | 0.3 | 2.6 | 6.6 | 12.7 | 22 |
| Inhaled glucocorticosteroids (ICS) | **2,366** | **16.6** | 6.6 | 9.9 | 15.3 | 21.2 | 30.1 | **7,063** | | **13.8** | 9.2 | 12 | 15.8 | 18.4 | 27.9 |
| Fixed combinations of SABA and SAMA | **666** | **4.7** | 0.2 | 1.3 | 3.6 | 7.1 | 11.1 | **1,417** | | **2.8** | 0.3 | 1.4 | 3.3 | 6.1 | 11 |
| Fixed combinations of SABA and ICS | **0** | **0** | 0 | 0 | 0 | 0 | 0 | **0** | | **0** | 0 | 0 | 0 | 0 | 0 |
| Fixed combinations of LABA and ICS | **5,404** | **38** | 12.6 | 25.1 | 35.6 | 51.4 | 64.9 | **14,600** | | **28.5** | 12.1 | 24 | 36.2 | 49.2 | 65.2 |
| Systemic glucocorticosteroids | **4,429** | **31.1** | 12 | 22.1 | 30.8 | 39.7 | 50.9 | **12,133** | | **23.7** | 10.3 | 21.5 | 29.2 | 40.2 | 52 |
| Systemic beta2-agonists | **66** | **0.5** | 0.5 | 0.3 | 0.5 | 0.5 | 0.5 | **242** | | **0.5** | 0.5 | 0.4 | 0.5 | 0.3 | 0.5 |
| Xanthines and adrenergics | **109** | **0.8** | 0.1 | 0.2 | 0.6 | 0.8 | 2 | **256** | | **0.5** | 0.2 | 0.2 | 0.6 | 1.2 | 1.3 |
| Roflumilast | **25** | **0.2** | 0 | 0 | 0 | 0.1 | 0.7 | **50** | | **0.1** | 0 | 0 | 0.1 | 0.3 | 0.3 |
| Nasal glucocorticosteroids | **733** | **5.1** | 5.4 | 4.9 | 5 | 5.3 | 5.2 | **2,620** | | **5.1** | 5.2 | 4.6 | 5.2 | 5 | 6.2 |
| Omalizumab | **0** | **0** | 0 | 0 | 0 | 0 | 0 | **0** | | **0** | 0 | 0 | 0 | 0 | 0 |
| Leukotriene receptor antagonists | **252** | **1.8** | 1.3 | 1.5 | 1.4 | 2.1 | 2.5 | **787** | | **1.5** | 1.1 | 1.4 | 1.5 | 2.4 | 2.7 |
| Cromoglicic acid | **0** | **0** | 0 | 0 | 0 | 0 | 0 | **0** | | **0** | 0 | 0 | 0 | 0 | 0 |
| Nedocromil | **0** | **0** | 0 | 0 | 0 | 0 | 0 | **0** | | **0** | 0 | 0 | 0 | 0 | 0 |
| Oxygen therapy | **2,092** | **14.7** | 2.4 | 12.2 | 17.2 | 18.4 | 23.3 | **5,701** | | **11.1** | 2.5 | 11 | 17.2 | 19 | 24.9 |
| Nebuliser therapy | **10** | **0.1** | 0 | 0 | 0 | 0 | 0.4 | **14** | | **0** | 0 | 0 | 0 | 0 | 0.3 |
| Cardiovascular medications | **10,658** | **74.9** | 72 | 73.2 | 76.2 | 75 | 77.8 | **37,750** | | **73.8** | 72.8 | 72.4 | 74.7 | 75.5 | 77.3 |
| Cardiac glycosides and antiarrhythmics, Class I and III | **937** | **6.6** | 3.6 | 5.2 | 7.1 | 7.4 | 9.6 | **2,926** | | **5.7** | 3.8 | 5.6 | 6.9 | 7.6 | 8.9 |
| Vasodilators used in cardiac diseases | **1,066** | **7.5** | 5.8 | 7.1 | 7.2 | 9 | 8.4 | **3,605** | | **7** | 6.1 | 6.9 | 7.7 | 7.9 | 8.7 |
| Cardiac stimulants and other cardiac preparations | **71** | **0.5** | 0.5 | 0.5 | 0.3 | 0.5 | 0.7 | **209** | | **0.4** | 0.3 | 0.5 | 0.5 | 0.4 | 0.3 |
| Diuretics | **5,759** | **40.4** | 28.9 | 37.9 | 42.9 | 43.2 | 49.2 | **18,931** | | **37** | 30 | 36.6 | 41.9 | 42.2 | 50.5 |
| Peripheral vasodilators | **0** | **0** | 0 | 0 | 0 | 0 | 0 | **<5** | | **0** | 0 | 0 | 0 | 0 | 0 |
| Vasoprotective agents | **348** | **2.4** | 2.5 | 2.6 | 2.3 | 2.4 | 2.4 | **1,301** | | **2.5** | 2.6 | 2.9 | 2.3 | 2.3 | 2 |
| Beta blocking agents | **3,952** | **27.8** | 29.2 | 26.5 | 30.2 | 27 | 26 | **14,386** | | **28.1** | 28.1 | 27 | 29.8 | 29 | 26.1 |
| Calcium channel blockers | **3,261** | **22.9** | 23.6 | 22.6 | 24.5 | 21.6 | 22.2 | **11,841** | | **23.1** | 24 | 21.7 | 23.9 | 23.1 | 22 |
| Antihypertensives | **173** | **1.2** | 0.8 | 1.1 | 1.6 | 1.3 | 1.3 | **553** | | **1.1** | 0.7 | 1.3 | 1.4 | 1.5 | 1 |
| Agents acting on the renin-angiotensin system | **5,282** | **37.1** | 41.1 | 38.7 | 38.1 | 34.4 | 33.2 | **19,806** | | **38.7** | 42.4 | 38.6 | 36.9 | 34.7 | 32.4 |
| Angiotensin-converting-enzyme inhibitors | **2,719** | **19.1** | 20.2 | 19.8 | 19.8 | 17.9 | 17.6 | **10,064** | | **19.7** | 20.9 | 19.6 | 19 | 18.3 | 17.9 |
| Angiotensin II receptor antagonists | **2,656** | **18.7** | 21.8 | 19.4 | 19.1 | 17.2 | 15.8 | **10,137** | | **19.8** | 22.6 | 19.8 | 18.4 | 16.9 | 14.8 |
| Renin-inhibitors | **<5** | **0** | 0 | 0 | 0 | 0 | 0 | **12** | | **0** | 0 | 0 | 0 | 0 | 0 |
| Lipid-modifying agents | **5,428** | **38.1** | 38.7 | 38.6 | 38.4 | 37.9 | 37 | **19,891** | | **38.9** | 40.7 | 38.2 | 37.7 | 38.8 | 35.6 |
| HMG-CoA reductase inhibitors (statins) | **5,334** | **37.5** | 38.3 | 37.9 | 37.7 | 37.1 | 36.3 | **19,539** | | **38.2** | 40 | 37.4 | 37.1 | 38 | 35 |
| Other lipid-modifying agents | **190** | **1.3** | 0.7 | 1.3 | 1.5 | 1.5 | 1.6 | **708** | | **1.4** | 1.3 | 1.4 | 1.5 | 1.4 | 1.3 |
| HMG-CoA reductase inhibitors (statins), other combinations with acetylsalicylic acid | **0** | **0** | 0 | 0 | 0 | 0 | 0 | **0** | | **0** | 0 | 0 | 0 | 0 | 0 |
| Antithrombotic agents | **6,887** | **48.4** | 43 | 45.8 | 49.3 | 50.3 | 53.5 | **23,956** | | **46.8** | 43.6 | 46.6 | 48 | 50.3 | 53.7 |
| Platelet aggregation inhibitors | **4,704** | **33** | 29.7 | 31.7 | 33.8 | 33.7 | 36.3 | **16,341** | | **31.9** | 29.7 | 32.3 | 32 | 34.8 | 36 |
| Systemic antibacterials | **8,214** | **57.7** | 38.4 | 49.5 | 59.3 | 67.3 | 73.9 | **26,105** | | **51** | 37.3 | 50.5 | 57.3 | 67.2 | 74.7 |
| Iron preparations | **290** | **2** | 1.3 | 1.7 | 2.7 | 2.3 | 2.2 | **900** | | **1.8** | 1.1 | 1.8 | 2.2 | 2.1 | 2.9 |
| Proton pump inhibitors | **4,973** | **34.9** | 28.6 | 32.7 | 36.3 | 37.7 | 39.5 | **17,075** | | **33.4** | 29.8 | 33.6 | 35.6 | 36.7 | 38.6 |
| Drugs used in diabetes | **1,837** | **12.9** | 12.8 | 13.9 | 13.1 | 12.6 | 12.2 | **6,742** | | **13.2** | 12.8 | 13.9 | 13.6 | 12.6 | 12.9 |
| Insulins | **602** | **4.2** | 3.5 | 4.2 | 4.6 | 4.4 | 4.5 | **2,053** | | **4** | 3.3 | 4.8 | 4 | 4.2 | 4.8 |
| Blood glucose–lowering drugs | **1,599** | **11.2** | 11.6 | 12.1 | 11.3 | 10.6 | 10.5 | **5,912** | | **11.6** | 11.5 | 11.8 | 11.7 | 11 | 11.5 |
| Drugs for musculoskeletal system | **1,982** | **13.9** | 16.2 | 13.8 | 14 | 13.4 | 12.2 | **7,242** | | **14.2** | 14.2 | 14 | 14 | 14.9 | 13.3 |
| Anti-inflammatory and antirheumatic products, non-steroids (non-steroidal anti-inflammatory drugs) | **1,916** | **13.5** | 15.7 | 13.3 | 13.6 | 12.9 | 11.7 | **7,041** | | **13.8** | 14 | 13.6 | 13.7 | 14.4 | 12.4 |
| Acetylsalicylic acid (other analgesics and antipyretics) | **89** | **0.6** | 0.6 | 0.6 | 0.4 | 0.7 | 0.8 | **273** | | **0.5** | 0.4 | 0.6 | 0.4 | 0.7 | 1 |
| Other antirheumatic agents: Anti-inflammatory/antirheumatic agents in combination, specific antirheumatic agents | **0** | **0** | 0 | 0 | 0 | 0 | 0 | **0** | | **0** | 0 | 0 | 0 | 0 | 0 |
| Antidepressants | **3,228** | **22.7** | 18.4 | 20.2 | 23.7 | 24.6 | 26.4 | **10,753** | | **21** | 17.4 | 21.2 | 22.9 | 25.2 | 26 |
| Selective serotonin reuptake inhibitors | **1,845** | **13** | 10.9 | 11.6 | 13.4 | 14.1 | 14.8 | **6,171** | | **12.1** | 10.1 | 12.1 | 12.6 | 14.7 | 15.3 |
| Antineoplastic agents | **11** | **0.1** | 0 | 0 | 0 | 0.1 | 0.3 | **27** | | **0.1** | 0 | 0.1 | 0 | 0.1 | 0.2 |
| Immunosuppressants | **196** | **1.4** | 1.6 | 1.5 | 1.6 | 1.2 | 1 | **738** | | **1.4** | 1.7 | 1.2 | 1.5 | 0.9 | 1.7 |
| Antivirals for systemic use | **175** | **1.2** | 0.6 | 1.2 | 1.2 | 1.4 | 1.8 | **568** | | **1.1** | 0.7 | 1.2 | 1.6 | 1.3 | 1.5 |
| Hormone-replacement therapy: Estrogens, progestogens, progestogens and estrogens in combination | **1,082** | **7.6** | 8.4 | 7.7 | 7.1 | 8.3 | 6.5 | **4,141** | | **8.1** | 9 | 8.1 | 7.5 | 7.2 | 6.7 |
| Drugs used in nicotine dependence | **414** | **2.9** | 1.1 | 1.9 | 3.3 | 3.9 | 4.4 | **1,210** | | **2.4** | 1.2 | 2.2 | 2.9 | 4.1 | 4 |
| **COPD severity category** |  |  |  |  |  |  |  |  | |  |  |  |  |  |  |
| Mild | **802** | **5.6** | 24.7 | 2.9 | 0.3 | 0.2 | 0 | **5,777** | | **11.3** | 28.1 | 3.1 | 0.4 | 0.2 | 0 |
| Moderate | **3,447** | **24.2** | 41.8 | 33.8 | 26.1 | 13.7 | 5.8 | **15,674** | | **30.6** | 42.7 | 34.9 | 24.9 | 12.8 | 5.2 |
| Severe | **6,651** | **46.7** | 27.7 | 43.5 | 48.6 | 57.7 | 56 | **20,444** | | **40** | 23.7 | 43.2 | 48.4 | 59 | 55.2 |
| Very severe | **3,339** | **23.4** | 5.8 | 19.8 | 25 | 28.4 | 38.2 | **9,272** | | **18.1** | 5.5 | 18.9 | 26.2 | 28 | 39.5 |
| **CCI score** |  |  |  |  |  |  |  |  | |  |  |  |  |  |  |
| 1 | **4,622** | **32.5** | 36.7 | 31.8 | 30.7 | 32.4 | 30.6 | | **16,865** | **33.0** | 35.8 | 32.7 | 30.2 | 31.1 | 30.0 |
| 2 | **2,806** | **19.7** | 19.2 | 20.3 | 19.5 | 19.7 | 19.8 | | **10,590** | **20.7** | 21.3 | 21.1 | 20.3 | 19.9 | 19.2 |
| 3+ | **6,811** | **47.8** | 44.1 | 47.8 | 49.8 | 47.8 | 49.6 | | **23,712** | **46.3** | 42.9 | 46.3 | 49.5 | 49.1 | 50.9 |
| Hip fracture | **144** | **1.0** | 0.5 | 0.9 | 1.3 | 1.0 | 1.4 | | **436** | **0.9** | 0.6 | 0.7 | 1.3 | 1.4 | 0.8 |
| Lung cancer | **432** | **3.0** | 2.7 | 3.3 | 3.5 | 2.8 | 2.8 | | **1,000** | **2.0** | 1.5 | 1.9 | 2.2 | 3.1 | 2.1 |
| Other markers of bad fall | **91** | **0.6** | 0.4 | 0.8 | 0.7 | 0.7 | 0.6 | | **343** | **0.7** | 0.6 | 1.0 | 0.5 | 0.4 | 0.9 |
| Metastatic cancer | **110** | **0.8** | 0.7 | 0.6 | 1.1 | 0.5 | 1.0 | | **287** | **0.6** | 0.4 | 0.5 | 0.7 | 0.8 | 0.9 |
| Pulmonary cachexia | **69** | **0.5** | 0.2 | 0.5 | 0.4 | 0.7 | 0.6 | | **168** | **0.3** | 0.2 | 0.3 | 0.4 | 0.5 | 0.5 |
| Right-sided heart failure | **135** | **0.9** | 0.6 | 0.9 | 1.2 | 1.1 | 1.0 | | **274** | **0.5** | 0.4 | 0.6 | 0.9 | 0.4 | 0.5 |
| **Number of hospitalisations within 180 days** |  |  |  |  |  |  |  | |  |  |  |  |  |  |  |
| 0 | **7,448** | **52.3** | 68.8 | 54.5 | 49.2 | 45.9 | 43.1 | | **33,178** | **64.8** | 76.4 | 62.5 | 57.8 | 55.4 | 49.8 |
| 1 | **4,029** | **28.3** | 22.0 | 29.2 | 29.9 | 30.2 | 30.1 | | **12,143** | **23.7** | 16.5 | 26.0 | 28.6 | 29.9 | 29.5 |
| 2 | **1,519** | **10.7** | 5.8 | 9.2 | 11.1 | 13.3 | 13.9 | | **3,511** | **6.9** | 5.0 | 6.8 | 7.6 | 7.9 | 12.2 |
| 3-4 | **963** | **6.8** | 3.0 | 5.8 | 7.6 | 8.0 | 9.5 | | **1,874** | **3.7** | 1.8 | 3.8 | 5.0 | 5.1 | 6.6 |
| 5+ | **280** | **2.0** | 0.4 | 1.2 | 2.1 | 2.6 | 3.5 | | **461** | **0.9** | 0.3 | 0.9 | 1.0 | 1.7 | 2.0 |
| **Number of hospitalisations within 365 days** |  |  |  |  |  |  |  | |  |  |  |  |  |  |  |
| 0 | **5,906** | **41.5** | 60.2 | 45.0 | 38.6 | 33.7 | 29.9 | | **26,565** | **51.9** | 65.8 | 48.9 | 43.8 | 39.3 | 35.6 |
| 1 | **4,050** | **28.4** | 23.3 | 29.0 | 30.4 | 30.4 | 29.1 | | **13,860** | **27.1** | 21.0 | 29.9 | 30.7 | 32.7 | 29.7 |
| 2 | **1,959** | **13.8** | 8.8 | 12.7 | 14.4 | 15.6 | 17.4 | | **5,610** | **11.0** | 7.8 | 11.2 | 13.3 | 13.1 | 16.4 |
| 3-4 | **1,539** | **10.8** | 6.1 | 8.5 | 10.5 | 13.5 | 15.5 | | **3,602** | **7.0** | 4.2 | 6.9 | 8.2 | 10.4 | 12.3 |
| 5+ | **785** | **5.5** | 1.6 | 4.7 | 6.2 | 6.8 | 8.2 | | **1,530** | **3.0** | 1.2 | 3.2 | 4.1 | 4.5 | 6.0 |
| **Number of hospitalisations with COPD within 90 days** |  |  |  |  |  |  |  | |  |  |  |  |  |  |  |
| 0 | **9,702** | **68.1** | 82.4 | 69.3 | 66.1 | 63.8 | 59.0 | | **41,596** | **81.3** | 90.4 | 78.9 | 76.4 | 73.8 | 69.6 |
| 1 | **3,577** | **25.1** | 15.8 | 25.9 | 27.4 | 27.4 | 29.2 | | **8,189** | **16.0** | 8.8 | 18.2 | 20.6 | 22.1 | 22.8 |
| 2+ | **960** | **6.7** | 1.8 | 4.7 | 6.6 | 8.8 | 11.8 | | **1,382** | **2.7** | 0.8 | 2.9 | 3.0 | 4.1 | 7.5 |
| **Number of hospitalisations with COPD within 180 days** |  |  |  |  |  |  |  | |  |  |  |  |  |  |  |
| 0 | **8,859** | **62.2** | 80.3 | 64.9 | 59.6 | 55.7 | 50.5 | | **38,753** | **75.7** | 87.7 | 74.2 | 69.2 | 63.7 | 59.7 |
| 1 | **3,882** | **27.3** | 17.0 | 28.3 | 30.0 | 30.0 | 31.1 | | **10,058** | **19.7** | 10.9 | 21.3 | 25.4 | 28.6 | 27.9 |
| 2+ | **1,498** | **10.5** | 2.7 | 6.8 | 10.4 | 14.3 | 18.3 | | **2,356** | **4.6** | 1.4 | 4.5 | 5.4 | 7.7 | 12.4 |
| **Number of COPD exacerbations within 90 days** |  |  |  |  |  |  |  | |  |  |  |  |  |  |  |
| 0 | **6,260** | **44.0** | 65.6 | 49.7 | 42.3 | 35.7 | 26.5 | | **28,582** | **55.9** | 72.5 | 54.1 | 48.1 | 38.5 | 30.6 |
| 1 | **4,425** | **31.1** | 25.7 | 31.3 | 32.7 | 32.0 | 33.7 | | **14,666** | **28.7** | 21.7 | 31.5 | 31.6 | 35.7 | 34.3 |
| 2 | **2,354** | **16.5** | 7.0 | 14.1 | 17.0 | 20.8 | 23.8 | | **5,787** | **11.3** | 4.7 | 11.1 | 15.0 | 18.1 | 22.6 |
| 3+ | **1,200** | **8.4** | 1.8 | 4.9 | 8.0 | 11.5 | 15.9 | | **2,132** | **4.2** | 1.1 | 3.3 | 5.2 | 7.7 | 12.5 |
| **Number of COPD exacerbations within 180 days** |  |  |  |  |  |  |  | |  |  |  |  |  |  |  |
| 0 | **4,680** | **32.9** | 56.8 | 39.8 | 30.0 | 22.8 | 15.0 | | **22,400** | **43.8** | 63.1 | 41.6 | 34.5 | 23.2 | 16.0 |
| 1 | **4,129** | **29.0** | 29.9 | 31.5 | 31.0 | 27.9 | 24.8 | | **14,909** | **29.1** | 26.0 | 32.5 | 30.4 | 31.6 | 27.1 |
| 2 | **2,643** | **18.6** | 8.6 | 16.9 | 20.7 | 22.0 | 24.6 | | **8,196** | **16.0** | 7.6 | 17.2 | 20.7 | 24.3 | 26.9 |
| 3+ | **2,787** | **19.6** | 4.8 | 11.9 | 18.3 | 27.2 | 35.7 | | **5,662** | **11.1** | 3.2 | 8.7 | 14.4 | 20.9 | 30.0 |

: Data were handled according to the Danish Act on Processing of Personal Data. The possibility of unintentional (deductive) disclosure arises when cells with small numbers of patients are quoted. When reporting the data, Danish policy is that no cell should contain fewer than 5 events. Cells with 0 counts are reported. Cells with “<5” indicate counts between 1 and 4, and exact number and percentage are not reported. To avoid back calculation, when needed, complementary cells are not reported.

Table S12. Patient Demographics, Clinical Characteristics, and Medications at the Index Date in Each Study Cohort After Trimming and Matching Stratified by PS Decile Groups, Restricting the Population to Users of FDCs of LABA/LAMA Who Were LABA Naive

|  | **Cohort** | | | | | | | | | | | | | | | | | | | | | | | | | | | | |
| --- | --- | --- | --- | --- | --- | --- | --- | --- | --- | --- | --- | --- | --- | --- | --- | --- | --- | --- | --- | --- | --- | --- | --- | --- | --- | --- | --- | --- | --- |
|  | **Olodaterol cohort** | | | | | | | | | | | | | **Other LABA cohort** | | | | | | | | | | | | | | | |
|  | **Overall** | | | **PS decile** | | | | | | | | | | **Overall** | | **PS decile** | | | | | | | | | | | | | |
|  |  |  |  | **1** | **2** | **3** | **4** | **5** | **6** | **7** | **8** | **9** | **10** |  |  | **1** | **2** | **3** | | **4** | | **5** | **6** | **7** | | **8** | | **9** | **10** |
|  | **N** | | **%** | **%** | **%** | **%** | **%** | **%** | **%** | **%** | **%** | **%** | **%** | **N** | **%** | **%** | **%** | **%** | **%** | | | **%** | **%** | **%** | | **%** | | **%** | **%** |
| Total | **5,677** | | **100** | 100 | 100 | 100 | 100 | 100 | 100 | 100 | 100 | 100 | 100 | **20,514** | 100 | 100 | 100 | 100 | 100 | | | 100 | 100 | 100 | | 100 | | 100 | 100 |
| **Age (years)** | **5,677** | |  | 567 | 568 | 568 | 567 | 568 | 569 | 566 | 569 | 567 | 568 | **20,514** |  | 4,098 | 3,330 | 2,643 | | 2,198 | | 1,900 | 1,569 | 1,496 | | 1,209 | | 1,125 | 946 |
| Mean (SD) | **72.4 (10.2)** | |  | 71.3 (9.9) | 71.1 (10.4) | 72.2 (9.9) | 72.0 (10.3) | 72.8 (9.8) | 72.0 (10.3) | 72.5 (10.3) | 72.9 (10.1) | 73.7 (10.2) | 73.4 (10.3) | **72.3 (10.0)** |  | 71.9 (9.8) | 71.8 (10.2) | 72.8 (10.3) | | 72.4 (9.9) | | 73.2 (10.1) | 72.4 (10.2) | 72.2(10.3) | | 73.0 (10.1) | | 72.5 (9.9) | 72.2 (9.7) |
| Median (Q1, Q3) | **73 (66, 80)** | |  | 72 (64, 79) | 72 (64, 79) | 73 (65, 80) | 72 (65, 80) | 74 (66, 80) | 73 (65, 79) | 73, (66, 81) | 73 (67, 81) | 74 (67, 81) | 74 (67, 81) | **73 (66, 80)** |  | 73 (65, 79) | 73 (65, 79) | 74 (66, 80) | | 73 (66, 80) | | 74 (67, 80) | 73 (65, 80) | 73 (66, 80) | | 74 (66, 80) | | 73 (67, 80) | 73 (66, 79) |
| Min, Max (rounded to 10 years) | **40, 100** | |  | 40, 90 | 40, 90 | 40, 90 | 40, 90 | 40, 90 | 40, 100 | 40, 100 | 40, 100 | 40, 100 | 40, 90 | **40, 100** |  | 40, 90 | 40, 100 | 40, 100 | | 40, 90 | | 40, 100 | 40, 90 | 40, 100 | | 40, 90 | | 40, 90 | 50, 90 |
| **Age group (years)** |  | |  |  |  |  |  |  |  |  |  |  |  |  |  |  |  |  | |  | |  |  |  | |  | |  |  |
| 40-60 | **762** | | **13** | 14 | 17 | 14 | 14 | 12 | 13 | 15 | 13 | 11 | 13 | **2,773** | **14** | 13 | 15 | 13 | 13 | | | 12 | 15 | 15 | | 13 | | 13 | 13 |
| 61-74 | **2,408** | | **42** | 47 | 43 | 41 | 44 | 42 | 45 | 40 | 44 | 40 | 39 | **8,784** | **43** | 45 | 43 | 41 | 44 | | | 41 | 43 | 39 | | 40 | | 46 | 44 |
| 75-84 | **1,865** | | **33** | 31 | 31 | 36 | 31 | 36 | 32 | 34 | 31 | 34 | 33 | **6,695** | **33** | 32 | 32 | 34 | 34 | | | 34 | 29 | 35 | | 33 | | 30 | 31 |
| 85+ | **642** | | **11** | 9 | 9 | 9 | 10 | 10 | 11 | 12 | 12 | 15 | 15 | **2,262** | **11** | 10 | 10 | 12 | 9 | | | 12 | 12 | 11 | | 13 | | 12 | 12 |
| **Gender** |  | |  |  |  |  |  |  |  |  |  |  |  |  |  |  |  |  |  | | |  |  |  | |  | |  |  |
| Female | **2,809** | | **49** | 46 | 49 | 51 | 55 | 48 | 49 | 47 | 50 | 54 | 45 | **10,154** | **49** | 48 | 49 | 51 | 49 | | | 51 | 50 | 52 | | 49 | | 49 | 47 |
| Male | **2,868** | | **51** | 54 | 51 | 49 | 45 | 52 | 51 | 53 | 50 | 46 | 55 | **10,360** | **51** | 52 | 51 | 49 | 51 | | | 49 | 50 | 48 | | 51 | | 51 | 53 |
| **Calendar year at index date** |  | |  |  |  |  |  |  |  |  |  |  |  |  |  |  |  |  |  | | |  |  |  | |  | |  |  |
| 2015 | **303** | | **5** | 4 | 6 | 7 | 8 | 8 | 4 | 5 | 3 | 4 | 5 | **1,085** | **5** | 4 | 5 | 5 | 6 | | | 6 | 6 | 5 | | 5 | | 6 | 5 |
| 2016 | **1,194** | | **21** | 23 | 21 | 21 | 24 | 23 | 23 | 20 | 18 | 21 | 18 | **4,302** | **21** | 21 | 20 | 22 | 19 | | | 24 | 23 | 21 | | 18 | | 21 | 22 |
| 2017 | **1,941** | | **34** | 36 | 33 | 34 | 33 | 30 | 33 | 35 | 37 | 35 | 36 | **7,044** | **34** | 35 | 34 | 33 | 33 | | | 35 | 37 | 38 | | 36 | | 29 | 32 |
| 2018 | **2,123** | | **37** | 37 | 40 | 38 | 35 | 38 | 39 | 38 | 38 | 37 | 36 | **7,817** | **38** | 39 | 41 | 38 | 40 | | | 34 | 33 | 34 | | 38 | | 41 | 37 |
| 2019 | **116** | | **2** | 0 | 1 | 1 | 1 | 1 | 2 | 2 | 3 | 3 | 6 | **266** | **1** | 0 | 0 | 1 | 1 | | | 1 | 2 | 2 | | 3 | | 3 | 3 |
| Cardiovascular diseases | **4,312** | | **76** | 67 | 74 | 73 | 71 | 73 | 71 | 76 | 85 | 84 | 87 | **15,127** | **74** | 68 | 72 | 77 | 74 | | | 75 | 75 | 75 | | 79 | | 76 | 82 |
| Ischaemic heart disease | **1,697** | | **30** | 27 | 30 | 29 | 30 | 29 | 26 | 25 | 32 | 35 | 34 | **6,133** | **30** | 29 | 31 | 32 | 30 | | | 29 | 29 | 29 | | 28 | | 27 | 32 |
| Angina pectoris | 1,255 | | 22 | 21 | 23 | 23 | 21 | 23 | 20 | 18 | 22 | 25 | 26 | 4,559 | 22 | 22 | 24 | 22 | 21 | | | 22 | 23 | 22 | | 23 | | 18 | 24 |
| Acute myocardial infarction | **626** | | **11** | 10 | 9 | 11 | 11 | 9 | 10 | 9 | 14 | 14 | 14 | **2,209** | **11** | 10 | 11 | 11 | 10 | | | 11 | 10 | 11 | | 12 | | 10 | 12 |
| Other acute or subacute ischaemic heart disease | **72** | | **1** | 1 | 1 | 1 | 2 | 1 | 1 | 1 | 2 | 2 | 1 | **247** | **1** | 1 | 1 | 2 | 1 | | | 2 | 1 | 2 | | 1 | | 1 | 2 |
| Chronic ischaemic heart disease | **1,117** | | **20** | 18 | 17 | 20 | 20 | 20 | 18 | 17 | 22 | 23 | 23 | **4,030** | **20** | 19 | 20 | 20 | 19 | | | 18 | 19 | 22 | | 18 | | 18 | 23 |
| Coronary reperfusion surgery and procedures | **682** | | **12** | 12 | 11 | 13 | 12 | 10 | 11 | 10 | 16 | 13 | 13 | **2,504** | **12** | 12 | 13 | 12 | 13 | | | 11 | 11 | 12 | | 12 | | 10 | 13 |
| Conduction disorders | **198** | | **3** | 2 | 4 | 4 | 3 | 3 | 2 | 3 | 5 | 5 | 5 | **669** | **3** | 2 | 3 | 3 | 2 | | | 4 | 3 | 3 | | 4 | | 6 | 5 |
| Cardiac arrest | **45** | | **1** | 0 | 0 | 1 | 1 | 0 | 1 | 1 | 1 | 1 | 1 | **116** | **1** | 0 | 0 | 1 | 0 | | | 1 | 0 | 1 | | 1 | | 1 | 1 |
| Arrhythmias | **1,426** | | **25** | 20 | 20 | 22 | 22 | 21 | 24 | 27 | 29 | 33 | 32 | **4,756** | **23** | 18 | 21 | 21 | 22 | | | 26 | 27 | 27 | | 28 | | 31 | 30 |
| Paroxysmal tachycardia | **270** | | **5** | 3 | 3 | 5 | 5 | 3 | 4 | 4 | 6 | 7 | 8 | **914** | **4** | 3 | 4 | 4 | 5 | | | 5 | 4 | 6 | | 7 | | 6 | 6 |
| Ventricular tachycardia | **73** | | **1** | 1 | 0 | 1 | 1 | 0 | 1 | 1 | 2 | 2 | 4 | **190** | **1** | 0 | 0 | 1 | 1 | | | 1 | 1 | 2 | | 1 | | 2 | 2 |
| Supraventricular tachycardia and unspecified | **171** | | **3** | 2 | 2 | 4 | 2 | 3 | 3 | 3 | 4 | 4 | 4 | **616** | **3** | 3 | 3 | 2 | 3 | | | 3 | 3 | 3 | | 4 | | 4 | 3 |
| Atrial fibrillation and flutter | **1,204** | | **21** | 16 | 16 | 17 | 19 | 19 | 21 | 23 | 25 | 28 | 27 | **3,966** | **19** | 14 | 17 | 18 | 17 | | | 20 | 23 | 23 | | 25 | | 27 | 26 |
| Other cardiac arrhythmias | **338** | | **6** | 6 | 5 | 7 | 6 | 4 | 5 | 5 | 7 | 9 | 7 | **1,151** | **6** | 5 | 6 | 4 | 7 | | | 7 | 5 | 7 | | 6 | | 8 | 7 |
| Ventricular fibrillation and flutter | **22** | | **0** | 1 | 0 | 1 | 0 | 0 | 0 | 1 | 0 | 0 | 0 | **71** | **0** | 0 | 0 | 0 | 0 | | | 1 | 0 | 1 | | 1 | | 0 | 0 |
| Other cardiac arrhythmias (subgroup) | **316** | | **6** | 5 | 4 | 6 | 6 | 4 | 5 | 4 | 7 | 9 | 7 | **1,094** | **5** | 4 | 5 | 3 | 6 | | | 6 | 5 | 6 | | 5 | | 7 | 6 |
| Heart failure | **1,021** | | **18** | 13 | 14 | 15 | 14 | 17 | 18 | 16 | 23 | 24 | 26 | **3,310** | **16** | 13 | 16 | 16 | 13 | | | 19 | 14 | 20 | | 18 | | 24 | 21 |
| Cerebrovascular disease | **1,081** | | **19** | 12 | 13 | 17 | 14 | 17 | 16 | 21 | 25 | 27 | 30 | **3,480** | **17** | 13 | 14 | 18 | 16 | | | 18 | 16 | 19 | | 20 | | 24 | 27 |
| Cerebral haemorrhage (subarachnoid, intracerebral, other non-traumatic) | **92** | | **2** | 0 | 1 | 1 | 1 | 1 | 2 | 2 | 3 | 4 | 3 | **270** | **1** | 0 | 1 | 1 | 1 | | | 1 | 1 | 2 | | 2 | | 2 | 5 |
| Cerebral infarction and stroke | **703** | | **12** | 6 | 7 | 9 | 8 | 10 | 10 | 16 | 16 | 20 | 22 | **2,177** | **11** | 7 | 8 | 10 | 11 | | | 12 | 11 | 13 | | 13 | | 18 | 16 |
| Transient ischaemic attack | **355** | | **6** | 5 | 4 | 7 | 4 | 6 | 5 | 5 | 6 | 10 | 11 | **1,171** | **6** | 5 | 5 | 6 | 6 | | | 4 | 5 | 6 | | 7 | | 9 | 10 |
| Other cerebrovascular disease and sequelae of cerebrovascular disease | **573** | | **10** | 5 | 7 | 9 | 8 | 9 | 8 | 11 | 13 | 14 | 17 | **1,762** | **9** | 6 | 7 | 10 | 9 | | | 9 | 7 | 10 | | 10 | | 14 | 15 |
| Hypertension and hypertensive heart disease | **2,178** | | **38** | 36 | 36 | 39 | 38 | 42 | 36 | 34 | 41 | 41 | 40 | **7,838** | **38** | 37 | 38 | 42 | 40 | | | 39 | 39 | 36 | | 36 | | 36 | 35 |
| Diseases of arteries, arterioles, and capillaries | **1,151** | | **20** | 20 | 24 | 23 | 18 | 19 | 15 | 21 | 21 | 19 | 23 | **4,186** | **20** | 19 | 20 | 23 | 23 | | | 20 | 18 | 20 | | 20 | | 21 | 23 |
| Peripheral arterial revascularisation procedures | **502** | | **9** | 7 | 9 | 9 | 7 | 10 | 8 | 10 | 12 | 8 | 10 | **1,711** | **8** | 7 | 8 | 10 | 10 | | | 7 | 8 | 9 | | 5 | | 10 | 13 |
| Other form of heart diseases | **1,703** | | **30** | 23 | 27 | 28 | 23 | 30 | 26 | 33 | 35 | 36 | 39 | **5,612** | **27** | 21 | 25 | 29 | 26 | | | 28 | 35 | 29 | | 31 | | 35 | 33 |
| Hyperlipidaemia | **1,161** | | **20** | 16 | 16 | 21 | 19 | 18 | 17 | 21 | 26 | 22 | 29 | **4,079** | **20** | 18 | 19 | 22 | 18 | | | 19 | 21 | 22 | | 20 | | 23 | 25 |
| Diabetes mellitus | **1,152** | | **20** | 17 | 16 | 18 | 17 | 20 | 16 | 20 | 26 | 26 | 28 | **3,789** | **18** | 17 | 15 | 17 | 17 | | | 20 | 18 | 21 | | 24 | | 23 | 24 |
| Renal disease | **1,735** | | **31** | 23 | 27 | 25 | 27 | 25 | 27 | 34 | 39 | 40 | 40 | **5,645** | **28** | 21 | 24 | 27 | 24 | | | 29 | 30 | 33 | | 38 | | 35 | 39 |
| Chronic kidney disease | **326** | | **6** | 5 | 3 | 5 | 5 | 5 | 4 | 8 | 8 | 7 | 8 | **1,039** | **5** | 4 | 4 | 5 | 6 | | | 6 | 5 | 5 | | 5 | | 7 | 8 |
| Other renal disorders | **1,525** | | **27** | 20 | 23 | 20 | 23 | 19 | 25 | 30 | 34 | 37 | 38 | **4,939** | **24** | 18 | 21 | 23 | 21 | | | 26 | 27 | 29 | | 34 | | 30 | 35 |
| Anaemias | **700** | | **12** | 7 | 9 | 11 | 9 | 10 | 10 | 15 | 15 | 17 | 20 | **2,124** | **10** | 5 | 10 | 10 | 11 | | | 10 | 11 | 15 | | 15 | | 13 | 16 |
| Nutritional anaemias | **286** | | **5** | 3 | 4 | 4 | 4 | 4 | 4 | 6 | 7 | 8 | 8 | **821** | **4** | 2 | 3 | 4 | 3 | | | 4 | 6 | 6 | | 8 | | 6 | 7 |
| Iron deficiency anaemias | **248** | | **4** | 2 | 3 | 3 | 3 | 3 | 4 | 6 | 5 | 6 | 7 | **728** | **4** | 2 | 3 | 4 | 3 | | | 4 | 5 | 5 | | 7 | | 6 | 5 |
| Other anaemias | **584** | | **10** | 5 | 8 | 10 | 7 | 8 | 8 | 13 | 11 | 15 | 18 | **1,785** | **9** | 4 | 9 | 8 | 9 | | | 9 | 9 | 12 | | 12 | | 11 | 12 |
| Peptic ulcer disease | **550** | | **10** | 7 | 8 | 8 | 10 | 8 | 9 | 10 | 12 | 11 | 13 | **1,847** | **9** | 7 | 8 | 8 | 9 | | | 10 | 10 | 8 | | 13 | | 12 | 12 |
| Liver disease | **246** | | **4** | 2 | 3 | 3 | 5 | 3 | 4 | 5 | 5 | 5 | 8 | **837** | **4** | 3 | 5 | 3 | 4 | | | 3 | 4 | 4 | | 4 | | 7 | 5 |
| Osteoporosis | **1,125** | | **20** | 11 | 15 | 17 | 17 | 19 | 19 | 21 | 25 | 27 | 27 | **3,656** | **18** | 13 | 15 | 17 | 19 | | | 18 | 20 | 23 | | 21 | | 21 | 27 |
| Rheumatoid arthritis and other inflammatory arthropathies | **574** | | **10** | 11 | 11 | 11 | 9 | 11 | 8 | 10 | 9 | 9 | 11 | **2,021** | **10** | 9 | 12 | 9 | 7 | | | 9 | 9 | 11 | | 11 | | 12 | 9 |
| Systemic connective tissue diseases | **256** | | **5** | 6 | 5 | 4 | 4 | 6 | 4 | 3 | 4 | 6 | 3 | **1,002** | **5** | 5 | 5 | 6 | 5 | | | 6 | 4 | 4 | | 3 | | 4 | 3 |
| Malignancy | **1,468** | | **26** | 25 | 22 | 23 | 26 | 26 | 22 | 27 | 29 | 31 | 29 | **4,975** | **24** | 23 | 22 | 24 | 25 | | | 19 | 26 | 29 | | 33 | | 24 | 29 |
| Depressive disorders | **356** | | **6** | 5 | 6 | 4 | 5 | 5 | 5 | 9 | 8 | 7 | 9 | **1,189** | **6** | 5 | 5 | 5 | 5 | | | 6 | 6 | 9 | | 7 | | 6 | 10 |
| Pregnancy (at the index date) | **0** | | **0** | 0 | 0 | 0 | 0 | 0 | 0 | 0 | 0 | 0 | 0 | **0** | **0** | 0 | 0 | 0 | 0 | | | 0 | 0 | 0 | | 0 | | 0 | 0 |
| Asthma | **665** | | **12** | 9 | 13 | 11 | 13 | 10 | 11 | 8 | 14 | 15 | 11 | **2,317** | **11** | 9 | 13 | 11 | 10 | | | 13 | 13 | 10 | | 10 | | 14 | 15 |
| Respiratory medications | **4,559** | | **80** | 47 | 68 | 77 | 83 | 83 | 85 | 84 | 89 | 92 | 95 | **14,897** | **73** | 45 | 66 | 76 | 82 | | | 81 | 83 | 82 | | 89 | | 89 | 94 |
| Inhaled short-acting muscarinic antagonists (SAMAs) | **19** | | **0** | 0 | 0 | 0 | 0 | 0 | 1 | 1 | 1 | 0 | 0 | **64** | **0** | 0 | 0 | 0 | 0 | | | 1 | 0 | 0 | | 0 | | 0 | 0 |
| Inhaled long-acting muscarinic antagonists (LAMAs) | **1,932** | | **34** | 2 | 11 | 32 | 43 | 40 | 36 | 32 | 37 | 49 | 58 | **5,528** | **27** | 1 | 11 | 27 | 40 | | | 39 | 42 | 35 | | 42 | | 46 | 63 |
| Inhaled short-acting beta2-agonists (SABAs) | **2,808** | | **49** | 32 | 41 | 39 | 45 | 51 | 52 | 50 | 59 | 61 | 64 | **9,191** | **45** | 30 | 41 | 43 | 47 | | | 49 | 50 | 50 | | 57 | | 59 | 63 |
| Inhaled long-acting beta2-agonists (LABAs) | **0** | | **0** | 0 | 0 | 0 | 0 | 0 | 0 | 0 | 0 | 0 | 0 | **0** | **0** | 0 | 0 | 0 | 0 | | | 0 | 0 | 0 | | 0 | | 0 | 0 |
| Inhaled glucocorticosteroids (ICS) | **711** | | **13** | 6 | 13 | 10 | 9 | 12 | 15 | 12 | 18 | 16 | 15 | **2,334** | **11** | 6 | 13 | 13 | 9 | | | 12 | 16 | 13 | | 13 | | 17 | 15 |
| Fixed combinations of SABA and SAMA | **200** | | **4** | 0 | 0 | 0 | 1 | 2 | 2 | 4 | 4 | 6 | 16 | **406** | **2** | 0 | 1 | 1 | 1 | | | 2 | 2 | 2 | | 3 | | 7 | 11 |
| Fixed combinations of SABA and ICS | **0** | | **0** | 0 | 0 | 0 | 0 | 0 | 0 | 0 | 0 | 0 | 0 | **0** | **0** | 0 | 0 | 0 | 0 | | | 0 | 0 | 0 | | 0 | | 0 | 0 |
| Fixed combinations of LABA and ICS | **0** | | **0** | 0 | 0 | 0 | 0 | 0 | 0 | 0 | 0 | 0 | 0 | **0** | **0** | 0 | 0 | 0 | 0 | | | 0 | 0 | 0 | | 0 | | 0 | 0 |
| Systemic glucocorticosteroids | **1,402** | | **25** | 9 | 18 | 20 | 22 | 24 | 27 | 27 | 31 | 32 | 38 | **4,367** | **21** | 11 | 16 | 21 | 21 | | | 25 | 26 | 27 | | 34 | | 31 | 36 |
| Systemic beta2-agonists | **18** | | **0** | 0 | 0 | 0 | 0 | 0 | 0 | 0 | 0 | 0 | 2 | **53** | **0** | 0 | 0 | 0 | 0 | | | 0 | 0 | 1 | | 0 | | 0 | 1 |
| Xanthines and adrenergics | **21** | | **0** | 0 | 0 | 0 | 0 | 0 | 0 | 0 | 1 | 1 | 1 | **68** | **0** | 0 | 0 | 0 | 1 | | | 1 | 1 | 0 | | 1 | | 1 | 0 |
| Roflumilast | **<5** | | **0** | 0 | 0 | 0 | 0 | 0 | 0 | 0 | 0 | 0 | 0 | **0** | **0** | 0 | 0 | 0 | 0 | | | 0 | 0 | 0 | | 0 | | 0 | 0 |
| Nasal glucocorticosteroids | **237** | | **4** | 6 | 6 | 5 | 5 | 4 | 3 | 3 | 4 | 3 | 3 | **938** | **5** | 5 | 6 | 5 | 5 | | | 5 | 4 | 3 | | 4 | | 3 | 5 |
| Omalizumab | **0** | | **0** | 0 | 0 | 0 | 0 | 0 | 0 | 0 | 0 | 0 | 0 | **0** | **0** | 0 | 0 | 0 | 0 | | | 0 | 0 | 0 | | 0 | | 0 | 0 |
| Leukotriene receptor antagonists | **40** | | **1** | 1 | 1 | 0 | 1 | 1 | 1 | 1 | 1 | 0 | 1 | **132** | **1** | 0 | 1 | 1 | 0 | | | 1 | 0 | 1 | | 0 | | 1 | 2 |
| Cromoglicic acid | **0** | | **0** | 0 | 0 | 0 | 0 | 0 | 0 | 0 | 0 | 0 | 0 | **0** | **0** | 0 | 0 | 0 | 0 | | | 0 | 0 | 0 | | 0 | | 0 | 0 |
| Nedocromil | **0** | | **0** | 0 | 0 | 0 | 0 | 0 | 0 | 0 | 0 | 0 | 0 | **0** | **0** | 0 | 0 | 0 | 0 | | | 0 | 0 | 0 | | 0 | | 0 | 0 |
| Oxygen therapy | **994** | | **18** | 2 | 8 | 10 | 14 | 17 | 20 | 24 | 28 | 25 | 26 | **2,804** | **14** | 3 | 7 | 11 | 13 | | | 20 | 20 | 23 | | 25 | | 31 | 21 |
| Nebuliser therapy | **<5** | | **0** | 0 | 0 | 0 | 0 | 0 | 0 | 0 | 0 | 0 | 0 | **<5** | **0** | 0 | 0 | 0 | 0 | | | 0 | 0 | 0 | | 0 | | 0 | 0 |
| Cardiovascular medications | **4,252** | | **75** | 69 | 71 | 72 | 72 | 76 | 70 | 73 | 81 | 82 | 82 | **15,058** | **73** | 70 | 73 | 71 | 72 | | | 75 | 75 | 76 | | 78 | | 81 | 79 |
| Cardiac glycosides and antiarrhythmics, Class I and III | **424** | | **7** | 4 | 3 | 5 | 6 | 5 | 9 | 9 | 8 | 12 | 13 | **1,256** | **6** | 4 | 4 | 5 | 3 | | | 7 | 11 | 8 | | 9 | | 13 | 11 |
| Vasodilators used in cardiac diseases | **434** | | **8** | 6 | 6 | 8 | 9 | 8 | 5 | 5 | 9 | 10 | 10 | **1,514** | **7** | 6 | 7 | 8 | 9 | | | 7 | 6 | 8 | | 9 | | 6 | 12 |
| Cardiac stimulants and other cardiac preparations | **21** | | **0** | 0 | 0 | 0 | 0 | 1 | 0 | 0 | 0 | 1 | 1 | **73** | **0** | 0 | 0 | 0 | 0 | | | 1 | 0 | 1 | | 0 | | 1 | 1 |
| Diuretics | **2,313** | | **41** | 25 | 29 | 31 | 35 | 40 | 39 | 42 | 57 | 51 | 58 | **7,558** | **37** | 28 | 33 | 36 | 37 | | | 36 | 40 | 44 | | 47 | | 51 | 48 |
| Peripheral vasodilators | **0** | | **0** | 0 | 0 | 0 | 0 | 0 | 0 | 0 | 0 | 0 | 0 | **0** | **0** | 0 | 0 | 0 | 0 | | | 0 | 0 | 0 | | 0 | | 0 | 0 |
| Vasoprotective agents | **136** | | **2** | 3 | 1 | 2 | 3 | 2 | 1 | 3 | 3 | 3 | 2 | **507** | **2** | 2 | 2 | 2 | 2 | | | 3 | 3 | 2 | | 3 | | 3 | 3 |
| Beta blocking agents | **1,785** | | **31** | 28 | 28 | 32 | 31 | 33 | 31 | 31 | 36 | 33 | 33 | **6,288** | **31** | 28 | 30 | 32 | 31 | | | 34 | 30 | 30 | | 33 | | 33 | 32 |
| Calcium channel blockers | **1,326** | | **23** | 20 | 20 | 21 | 23 | 25 | 23 | 27 | 23 | 26 | 27 | **4,741** | **23** | 20 | 22 | 25 | 25 | | | 22 | 25 | 23 | | 24 | | 21 | 27 |
| Antihypertensives | **75** | | **1** | 0 | 1 | 0 | 1 | 0 | 0 | 1 | 2 | 2 | 4 | **195** | **1** | 0 | 0 | 1 | 1 | | | 1 | 1 | 1 | | 3 | | 2 | 2 |
| Agents acting on the renin-angiotensin system | **2,173** | | **38** | 40 | 38 | 40 | 38 | 41 | 36 | 34 | 40 | 39 | 37 | **7,989** | **39** | 39 | 41 | 42 | 41 | | | 39 | 37 | 36 | | 34 | | 37 | 35 |
| Angiotensin-converting-enzyme inhibitors | **1,156** | | **20** | 19 | 20 | 22 | 19 | 24 | 20 | 16 | 22 | 21 | 20 | **4,186** | **20** | 19 | 21 | 23 | 23 | | | 20 | 20 | 18 | | 17 | | 20 | 19 |
| Angiotensin II receptor antagonists | **1,052** | | **19** | 22 | 19 | 20 | 20 | 18 | 16 | 18 | 18 | 18 | 17 | **3,950** | **19** | 21 | 22 | 20 | 18 | | | 20 | 18 | 18 | | 17 | | 18 | 16 |
| Renin-inhibitors | **0** | | **0** | 0 | 0 | 0 | 0 | 0 | 0 | 0 | 0 | 0 | 0 | **5** | **0** | 0 | 0 | 0 | 0 | | | 0 | 0 | 0 | | 0 | | 0 | 0 |
| Lipid-modifying agents | **2,199** | | **39** | 40 | 40 | 45 | 39 | 38 | 34 | 35 | 38 | 39 | 38 | **8,194** | **40** | 43 | 43 | 42 | 41 | | | 37 | 42 | 36 | | 33 | | 34 | 34 |
| HMG-CoA reductase inhibitors (statins) | **2,161** | | **38** | 39 | 40 | 44 | 39 | 38 | 34 | 35 | 37 | 39 | 38 | **8,046** | **39** | 42 | 42 | 41 | 40 | | | 37 | 41 | 36 | | 33 | | 33 | 34 |
| Other lipid-modifying agents | **68** | | **1** | 2 | 1 | 2 | 1 | 1 | 1 | 1 | 1 | 1 | 1 | **263** | **1** | 1 | 2 | 1 | 1 | | | 1 | 2 | 1 | | 1 | | 1 | 1 |
| HMG-CoA reductase inhibitors (statins), other combinations with acetylsalicylic acid | **0** | | **0** | 0 | 0 | 0 | 0 | 0 | 0 | 0 | 0 | 0 | 0 | **0** | **0** | 0 | 0 | 0 | 0 | | | 0 | 0 | 0 | | 0 | | 0 | 0 |
| Antithrombotic agents | **2,867** | | **51** | 41 | 45 | 46 | 44 | 45 | 47 | 55 | 59 | 60 | 62 | **9,795** | **48** | 41 | 45 | 46 | 47 | | | 51 | 53 | 53 | | 52 | | 54 | 59 |
| Platelet aggregation inhibitors | **1,886** | | **33** | 28 | 32 | 32 | 30 | 30 | 30 | 35 | 38 | 39 | 38 | **6,575** | **32** | 29 | 31 | 30 | 33 | | | 33 | 32 | 35 | | 33 | | 33 | 40 |
| Systemic antibacterials | **3,005** | | **53** | 29 | 42 | 44 | 47 | 56 | 61 | 57 | 61 | 66 | 67 | **9,620** | **47** | 27 | 39 | 46 | 48 | | | 53 | 58 | 57 | | 65 | | 68 | 66 |
| Iron preparations | **131** | | **2** | 1 | 1 | 1 | 1 | 2 | 2 | 2 | 4 | 5 | 4 | **385** | **2** | 1 | 1 | 1 | 2 | | | 2 | 2 | 3 | | 3 | | 6 | 5 |
| Proton pump inhibitors | **1,990** | | **35** | 22 | 27 | 30 | 34 | 30 | 31 | 39 | 41 | 44 | 53 | **6,584** | **32** | 24 | 29 | 29 | 34 | | | 32 | 33 | 39 | | 41 | | 40 | 50 |
| Drugs used in diabetes | **860** | | **15** | 14 | 12 | 13 | 13 | 15 | 13 | 15 | 18 | 19 | 19 | **2,879** | **14** | 13 | 12 | 13 | 11 | | | 14 | 13 | 16 | | 19 | | 20 | 18 |
| Insulins | **296** | | **5** | 3 | 4 | 3 | 4 | 4 | 4 | 6 | 7 | 9 | 9 | **879** | **4** | 3 | 3 | 3 | 4 | | | 4 | 5 | 5 | | 6 | | 9 | 10 |
| Blood glucose–lowering drugs | **744** | | **13** | 13 | 11 | 12 | 11 | 13 | 12 | 12 | 15 | 16 | 15 | **2,561** | **12** | 13 | 11 | 12 | 10 | | | 13 | 11 | 14 | | 17 | | 17 | 13 |
| Drugs for musculoskeletal system | **738** | | **13** | 16 | 13 | 14 | 15 | 14 | 11 | 10 | 13 | 11 | 13 | **2,744** | **13** | 14 | 14 | 16 | 12 | | | 15 | 11 | 11 | | 8 | | 14 | 12 |
| Anti-inflammatory and antirheumatic products, non-steroids (non-steroidal anti-inflammatory drugs) | **711** | | **13** | 16 | 13 | 14 | 15 | 14 | 10 | 10 | 12 | 10 | 12 | **2,675** | **13** | 14 | 14 | 16 | 12 | | | 15 | 11 | 11 | | 7 | | 14 | 10 |
| Acetylsalicylic acid (other analgesics and antipyretics) | **32** | | **1** | 0 | 0 | 0 | 0 | 1 | 1 | 1 | 1 | 1 | 1 | **88** | **0** | 0 | 0 | 0 | 0 | | | 1 | 0 | 0 | | 1 | | 0 | 2 |
| Other antirheumatic agents: Anti-inflammatory/antirheumatic agents in combination, specific antirheumatic agents | **0** | | **0** | 0 | 0 | 0 | 0 | 0 | 0 | 0 | 0 | 0 | 0 | **0** | **0** | 0 | 0 | 0 | 0 | | | 0 | 0 | 0 | | 0 | | 0 | 0 |
| Antidepressants | **1,287** | | **23** | 16 | 19 | 19 | 19 | 20 | 21 | 23 | 30 | 30 | 29 | **4,252** | **21** | 15 | 17 | 20 | 21 | | | 24 | 19 | 28 | | 25 | | 25 | 33 |
| Selective serotonin reuptake inhibitors | **722** | | **13** | 9 | 11 | 10 | 10 | 11 | 12 | 13 | 18 | 18 | 16 | **2,349** | **11** | 8 | 10 | 12 | 12 | | | 14 | 10 | 13 | | 13 | | 14 | 21 |
| Antineoplastic agents | **<5** | | **0** | 0 | 0 | 0 | 0 | 0 | 0 | 0 | 0 | 0 | 0 | **12** | **0** | 0 | 0 | 0 | 0 | | | 0 | 0 | 0 | | 0 | | 0 | 0 |
| Immunosuppressants | **91** | | **2** | 1 | 1 | 1 | 3 | 3 | 1 | 1 | 2 | 2 | 1 | **300** | **1** | 1 | 2 | 2 | 1 | | | 3 | 1 | 2 | | 1 | | 1 | 1 |
| Antivirals for systemic use | **50** | | **1** | 2 | 2 | 1 | 1 | 0 | 0 | 0 | 1 | 1 | 1 | **214** | **1** | 1 | 1 | 2 | 1 | | | 1 | 1 | 1 | | 1 | | 0 | 0 |
| Hormone-replacement therapy: Estrogens, progestogens, progestogens and estrogens in combination | **319** | | **6** | 7 | 8 | 7 | 7 | 6 | 4 | 4 | 6 | 3 | 4 | **1,340** | **7** | 9 | 9 | 7 | 7 | | | 6 | 5 | 3 | | 5 | | 4 | 3 |
| Drugs used in nicotine dependence | **169** | | **3** | 1 | 3 | 2 | 2 | 2 | 3 | 4 | 3 | 4 | 5 | **550** | **3** | 2 | 2 | 3 | 3 | | | 3 | 3 | 4 | | 2 | | 5 | 4 |
| **COPD severity category** |  | |  |  |  |  |  |  |  |  |  |  |  |  |  |  |  |  |  | | |  |  |  | |  | |  |  |
| Mild | **718** | | **13** | 65 | 29 | 16 | 7 | 3 | 2 | 2 | 2 | 1 | 1 | **4,382** | **21** | 65 | 27 | 16 | 9 | | | 5 | 2 | 2 | | 1 | | 1 | 0 |
| Moderate | **1,101** | | **19** | 22 | 25 | 33 | 35 | 27 | 18 | 12 | 11 | 8 | 4 | **4,553** | **22** | 19 | 29 | 30 | 36 | | | 25 | 23 | 12 | | 9 | | 6 | 5 |
| Severe | **2,400** | | **42** | 9 | 28 | 32 | 36 | 46 | 51 | 53 | 49 | 58 | 60 | **7,258** | **35** | 12 | 27 | 32 | 35 | | | 43 | 49 | 52 | | 54 | | 55 | 64 |
| Very severe | **1,458** | | **26** | 4 | 19 | 20 | 22 | 24 | 29 | 34 | 38 | 33 | 35 | **4,321** | **21** | 4 | 17 | 22 | 20 | | | 27 | 26 | 33 | | 36 | | 38 | 31 |
| **CCI score** |  | |  |  |  |  |  |  |  |  |  |  |  |  |  |  |  |  |  | | |  |  |  | |  | |  |  |
| 1 | | **1,649** | **29** | 36 | 35 | 32 | 34 | 33 | 34 | 28 | 22 | 20 | 16 | **6,513** | **32** | 37 | 35 | 30 | | | 34 | 33 | 33 | 29 | 22 | | 26 | | 16 |
| 2 | | **11,04** | **19** | 20 | 21 | 20 | 19 | 19 | 20 | 17 | 19 | 20 | 19 | **4,239** | **21** | 21 | 23 | 21 | | | 18 | 21 | 19 | 18 | 21 | | 19 | | 23 |
| 3+ | | **2,924** | **52** | 44 | 44 | 48 | 47 | 48 | 46 | 55 | 59 | 60 | 64 | **9,762** | **48** | 42 | 43 | 49 | | | 48 | 45 | 48 | 53 | 57 | | 54 | | 61 |
| Hip fracture | | **62** | **1** | 0 | 1 | 1 | 1 | 1 | 0 | 2 | 2 | 2 | 2 | **183** | **1** | 0 | 1 | 0 | | | 0 | 2 | 2 | 1 | 2 | | 2 | | 2 |
| Lung cancer | | **197** | **3** | 1 | 2 | 3 | 2 | 3 | 3 | 5 | 5 | 5 | 5 | **501** | **2** | 1 | 1 | 1 | | | 1 | 3 | 2 | 3 | 6 | | 5 | | 9 |
| Other markers of bad fall | | **40** | **1** | 0 | 1 | 0 | 0 | 1 | 1 | 1 | 1 | 1 | 2 | **132** | **1** | 0 | 1 | 0 | | | 1 | 1 | 0 | 1 | 1 | | 1 | | 1 |
| Metastatic cancer | | **52** | **1** | 1 | 0 | 1 | 1 | 0 | 1 | 1 | 2 | 1 | 2 | **128** | **1** | 0 | 0 | 0 | | | 0 | 1 | 0 | 1 | 3 | | 2 | | 1 |
| Pulmonary cachexia | | **27** | **0** | 0 | 0 | 1 | 0 | 0 | 1 | 1 | 1 | 1 | 1 | **76** | **0** | 0 | 0 | 0 | | | 0 | 0 | 0 | 1 | 0 | | 2 | | 1 |
| Right-sided heart failure | | **60** | **1** | 0 | 0 | 1 | 0 | 0 | 1 | 2 | 3 | 2 | 2 | **111** | **1** | 0 | 0 | 0 | | | 1 | 1 | 1 | 1 | 1 | | 1 | | 1 |
| **Number of hospitalisations within 180 days** | |  |  |  |  |  |  |  |  |  |  |  |  |  |  |  |  |  | | |  |  |  |  |  | |  | |  |
| 0 | | **2,824** | **50** | 96 | 88 | 74 | 67 | 52 | 40 | 32 | 27 | 14 | 8 | **12,959** | **63** | 97 | 86 | 74 | | | 66 | 53 | 46 | 31 | 25 | | 12 | | 6 |
| 1 | | **1,871** | **33** | 4 | 10 | 23 | 26 | 38 | 48 | 45 | 42 | 48 | 47 | **5,236** | **26** | 2 | 13 | 21 | | | 28 | 37 | 42 | 46 | 45 | | 45 | | 47 |
| 2 | | **561** | **10** | 0 | 2 | 3 | 5 | 7 | 9 | 14 | 19 | 20 | 21 | **1,373** | **7** | 0 | 1 | 4 | | | 4 | 8 | 9 | 15 | 16 | | 21 | | 24 |
| 3-4 | | **321** | **6** | 0 | 0 | 1 | 1 | 3 | 3 | 7 | 9 | 14 | 18 | **732** | **4** | 0 | 0 | 1 | | | 1 | 1 | 3 | 7 | 10 | | 18 | | 20 |
| 5+ | | **100** | **2** | 0 | 0 | 0 | 1 | 1 | 1 | 3 | 3 | 4 | 5 | **214** | **1** | 0 | 0 | 0 | | | 1 | 1 | 1 | 2 | 3 | | 4 | | 3 |
| **Number of hospitalisations within 365 days** | |  |  |  |  |  |  |  |  |  |  |  |  |  |  |  |  |  | | |  |  |  |  |  | |  | |  |
| 0 | | **2,176** | **38** | 83 | 67 | 58 | 51 | 41 | 28 | 22 | 17 | 11 | 4 | **10,265** | **50** | 83 | 66 | 58 | | | 53 | 38 | 36 | 22 | 19 | | 8 | | 3 |
| 1 | | **1,871** | **33** | 12 | 22 | 26 | 31 | 38 | 47 | 42 | 39 | 37 | 35 | **5,751** | **28** | 13 | 24 | 28 | | | 30 | 36 | 38 | 39 | 39 | | 33 | | 35 |
| 2 | | **788** | **14** | 3 | 6 | 10 | 12 | 12 | 15 | 16 | 20 | 21 | 24 | **2,400** | **12** | 3 | 7 | 10 | | | 11 | 16 | 16 | 19 | 17 | | 23 | | 25 |
| 3-4 | | **552** | **10** | 1 | 3 | 5 | 4 | 7 | 7 | 12 | 17 | 18 | 22 | **1,542** | **8** | 1 | 2 | 4 | | | 4 | 8 | 7 | 14 | 18 | | 25 | | 27 |
| 5+ | | **290** | **5** | 0 | 1 | 1 | 2 | 2 | 3 | 8 | 7 | 13 | 15 | **556** | **3** | 0 | 1 | 1 | | | 2 | 2 | 2 | 5 | 7 | | 11 | | 10 |
| **Number of hospitalisations with COPD within 90 days** | |  |  |  |  |  |  |  |  |  |  |  |  |  |  |  |  |  | | |  |  |  |  |  | |  | |  |
| 0 | | **3,821** | **67** | 99 | 98 | 87 | 81 | 67 | 57 | 53 | 52 | 42 | 37 | **16,665** | **81** | 100 | 97 | 91 | | | 84 | 78 | 68 | 60 | 57 | | 46 | | 45 |
| 1 | | **1,614** | **28** | 1 | 2 | 13 | 17 | 32 | 40 | 41 | 41 | 47 | 49 | **3,345** | **16** | 0 | 3 | 8 | | | 15 | 22 | 29 | 35 | 36 | | 44 | | 41 |
| 2+ | | **242** | **4** | 0 | 0 | 0 | 1 | 1 | 3 | 6 | 6 | 11 | 14 | **504** | **2** | 0 | 0 | 1 | | | 1 | 1 | 3 | 5 | 7 | | 10 | | 14 |
| **Number of hospitalisations with COPD within 180 days** | |  |  |  |  |  |  |  |  |  |  |  |  |  |  |  |  |  | | |  |  |  |  |  | |  | |  |
| 0 | | **3,543** | **62** | 99 | 97 | 86 | 79 | 62 | 54 | 46 | 44 | 31 | 24 | **15,668** | **76** | 100 | 97 | 89 | | | 81 | 71 | 63 | 52 | 46 | | 31 | | 22 |
| 1 | | **1,746** | **31** | 1 | 3 | 13 | 19 | 36 | 43 | 44 | 45 | 52 | 52 | **4,037** | **20** | 0 | 3 | 10 | | | 18 | 27 | 33 | 40 | 44 | | 52 | | 54 |
| 2+ | | **388** | **7** | 0 | 0 | 0 | 2 | 2 | 4 | 10 | 10 | 17 | 23 | **809** | **4** | 0 | 0 | 1 | | | 1 | 2 | 4 | 8 | 11 | | 17 | | 23 |
| **Number of COPD exacerbations within 90 days** | |  |  |  |  |  |  |  |  |  |  |  |  |  |  |  |  |  | | |  |  |  |  |  | |  | |  |
| 0 | | **2,599** | **46** | 85 | 72 | 63 | 58 | 46 | 33 | 31 | 29 | 23 | 19 | **11,728** | **57** | 84 | 71 | 63 | | | 59 | 50 | 41 | 37 | 28 | | 23 | | 25 |
| 1 | | **1,929** | **34** | 13 | 22 | 25 | 27 | 37 | 44 | 45 | 45 | 43 | 39 | **5,932** | **29** | 13 | 25 | 28 | | | 28 | 36 | 40 | 40 | 41 | | 44 | | 35 |
| 2 | | **864** | **15** | 2 | 5 | 12 | 14 | 15 | 19 | 19 | 19 | 22 | 26 | **2,210** | **11** | 2 | 5 | 8 | | | 12 | 13 | 15 | 18 | 22 | | 24 | | 22 |
| 3+ | | **285** | **5** | 0 | 1 | 1 | 1 | 3 | 3 | 5 | 8 | 12 | 16 | **644** | **3** | 0 | 0 | 1 | | | 1 | 2 | 4 | 5 | 10 | | 9 | | 19 |
| **Number of COPD exacerbations within 180 days** | |  |  |  |  |  |  |  |  |  |  |  |  |  |  |  |  |  | | |  |  |  |  |  | |  | |  |
| 0 | | **2,048** | **36** | 80 | 63 | 54 | 50 | 31 | 24 | 22 | 16 | 13 | 8 | **9,561** | **47** | 78 | 62 | 52 | | | 48 | 38 | 28 | 25 | 13 | | 9 | | 6 |
| 1 | | **1,842** | **32** | 17 | 22 | 24 | 26 | 39 | 41 | 39 | 44 | 38 | 34 | **5,987** | **29** | 17 | 25 | 27 | | | 29 | 34 | 42 | 38 | 40 | | 39 | | 33 |
| 2 | | **1,055** | **19** | 3 | 13 | 18 | 18 | 23 | 25 | 23 | 20 | 22 | 21 | **3,187** | **16** | 4 | 12 | 17 | | | 16 | 21 | 20 | 23 | 23 | | 24 | | 24 |
| 3+ | | **732** | **13** | 0 | 3 | 3 | 6 | 7 | 10 | 15 | 20 | 27 | 37 | **1,779** | **9** | 1 | 1 | 4 | | | 7 | 7 | 10 | 14 | 24 | | 28 | | 37 |

Data were handled according to the Danish Act on Processing of Personal Data. The possibility of unintentional (deductive) disclosure arises when cells with small numbers of patients are quoted. When reporting the data, Danish policy is that no cell should contain fewer than 5 events. Cells with 0 counts are reported. Cells with “<5” indicate counts between 1 and 4, and exact number and percentage are not reported. To avoid back calculation, when needed, complementary cells are not reported.

Table S13. Standardised Bias for the Comparison of Key Covariates Between New Users of Olodaterol and of Other LABAs in Propensity Score–Trimmed Study Cohorts by Propensity Score Deciles, Restricting the Population to Users of FDCs of LABA/LAMA Who Were LABA Naive

| **Variables** | **Untrimmed (overall)** | **Trimmed (overall)** | **Trimmed (averaged)** | STdiff D1 | STdiff D2 | STdiff D3 | STdiff D4 | STdiff D5 | STdiff D6 | STdiff D7 | STdiff D8 | STdiff D9 | STdiff D10 |
| --- | --- | --- | --- | --- | --- | --- | --- | --- | --- | --- | --- | --- | --- |
| Age group (years): 40-60 | **-0.00816** | **0.00278** | **0.002895** | -0.02526 | -0.06088 | -0.01559 | -0.03440 | -0.00238 | 0.06512 | 0.01296 | 0.01472 | 0.05055 | 0.02411 |
| Age group (years): 61-74 | **0.00882** | **0.00814** | **0.003861** | -0.02258 | -0.01176 | -0.00758 | -0.01377 | -0.01904 | -0.02623 | -0.00829 | -0.06737 | 0.11526 | 0.09996 |
| Age group (years): 75-84 | **0.00442** | **-0.00460** | **-0.008279** | 0.03311 | 0.02322 | -0.05162 | 0.05943 | -0.02628 | -0.05089 | 0.02268 | 0.04695 | -0.08345 | -0.05594 |
| Age group (years): 85 or more | **-0.01169** | **-0.00901** | **0.001427** | 0.01423 | 0.05477 | 0.10232 | -0.03383 | 0.06847 | 0.03926 | -0.03696 | 0.01752 | -0.11298 | -0.09854 |
| Female | **0.00229** | **0.00035** | **0.000848** | 0.03768 | -0.00453 | -0.00334 | -0.10440 | 0.04854 | 0.01105 | 0.10196 | -0.01913 | -0.09808 | 0.03875 |
| Male | **-0.00229** | **-0.00035** | **-0.000848** | -0.03768 | 0.00453 | 0.00334 | 0.10440 | -0.04854 | -0.01105 | -0.10196 | 0.01913 | 0.09808 | -0.03875 |
| Calendar year at index: 2015 | **0.00024** | **-0.00216** | **0.004407** | 0.00895 | -0.06029 | -0.05062 | -0.07517 | -0.06848 | 0.10560 | 0.00508 | 0.07152 | 0.09159 | 0.01590 |
| Calendar year at index: 2016 | **0.00733** | **-0.00150** | **-0.000359** | -0.05324 | -0.02491 | 0.01770 | -0.10881 | 0.01752 | -0.00109 | 0.04022 | -0.00399 | 0.00625 | 0.10677 |
| Calendar year at index: 2017 | **0.00521** | **0.00309** | **-0.001188** | -0.02480 | 0.03444 | -0.00754 | 0.00695 | 0.10622 | 0.06497 | 0.04972 | -0.02124 | -0.13641 | -0.08419 |
| Calendar year at index: 2018-2019 | **-0.01131** | **-0.00077** | **-0.003248** | 0.06479 | 0.01325 | 0.01571 | 0.11635 | -0.08651 | -0.11852 | -0.08657 | -0.00748 | 0.07456 | -0.01807 |
| Cardiovascular diseases: No | **0.05487** | **0.05035** | **0.017054** | -0.01280 | 0.05020 | -0.09361 | -0.05755 | -0.05692 | -0.09957 | 0.00760 | 0.14172 | 0.17689 | 0.11458 |
| Cardiovascular diseases: Yes | **-0.05487** | **-0.05035** | **-0.017054** | 0.01280 | -0.05020 | 0.09361 | 0.05755 | 0.05692 | 0.09957 | -0.00760 | -0.14172 | -0.17689 | -0.11458 |
| Ischaemic heart disease: No | **-0.00523** | **-0.00009** | **0.004922** | -0.05878 | -0.01016 | -0.07212 | 0.00771 | -0.00199 | -0.05538 | -0.08537 | 0.09944 | 0.17885 | 0.04702 |
| Ischaemic heart disease: Yes | **0.00523** | **0.00009** | **-0.004922** | 0.05878 | 0.01016 | 0.07212 | -0.00771 | 0.00199 | 0.05538 | 0.08537 | -0.09944 | -0.17885 | -0.04702 |
| Angina pectoris: No | **-0.01319** | **-0.00282** | **0.002061** | -0.03126 | -0.01692 | 0.00418 | 0.00468 | 0.01164 | -0.06903 | -0.09160 | -0.00820 | 0.16692 | 0.05021 |
| Angina pectoris: Yes | **0.01319** | **0.00282** | **-0.002061** | 0.03126 | 0.01692 | -0.00418 | -0.00468 | -0.01164 | 0.06903 | 0.09160 | 0.00820 | -0.16692 | -0.05021 |
| Acute myocardial infarction: No | **0.00613** | **0.00835** | **0.002916** | 0.00570 | -0.07991 | -0.02598 | 0.01445 | -0.05269 | 0.00680 | -0.05910 | 0.05547 | 0.12831 | 0.03610 |
| Acute myocardial infarction: Yes | **-0.00613** | **-0.00835** | **-0.002916** | -0.00570 | 0.07991 | 0.02598 | -0.01445 | 0.05269 | -0.00680 | 0.05910 | -0.05547 | -0.12831 | -0.03610 |
| Other acute or subacute ischaemic heart disease: No | **-0.00280** | **0.00589** | **0.016016** | -0.01420 | 0.01778 | -0.04996 | 0.11304 | -0.06248 | 0.02492 | -0.08188 | 0.06256 | 0.19314 | -0.04277 |
| Other acute or subacute ischaemic heart disease: Yes | **0.00280** | **-0.00589** | **-0.016016** | 0.01420 | -0.01778 | 0.04996 | -0.11304 | 0.06248 | -0.02492 | 0.08188 | -0.06256 | -0.19314 | 0.04277 |
| Chronic ischaemic heart disease: No | **-0.00286** | **0.00077** | **0.001376** | -0.04637 | -0.08608 | -0.01928 | 0.01896 | 0.02892 | -0.00240 | -0.12442 | 0.10231 | 0.12901 | 0.01311 |
| Chronic ischaemic heart disease: Yes | **0.00286** | **-0.00077** | **-0.001376** | 0.04637 | 0.08608 | 0.01928 | -0.01896 | -0.02892 | 0.00240 | 0.12442 | -0.10231 | -0.12901 | -0.01311 |
| Coronary reperfusion surgery and procedures: No | **-0.01444** | **-0.00589** | **-0.000428** | -0.02510 | -0.05058 | 0.01389 | -0.01305 | -0.01864 | -0.01807 | -0.07677 | 0.11695 | 0.08904 | -0.02197 |
| Coronary reperfusion surgery and procedures: Yes | **0.01444** | **0.00589** | **0.000428** | 0.02510 | 0.05058 | -0.01389 | 0.01305 | 0.01864 | 0.01807 | 0.07677 | -0.11695 | -0.08904 | 0.02197 |
| Conduction disorders: No | **0.01822** | **0.01276** | **-0.005943** | -0.00868 | 0.01746 | 0.03670 | 0.06715 | -0.07381 | -0.06840 | -0.04508 | 0.03055 | -0.04298 | 0.02767 |
| Conduction disorders: Yes | **-0.01822** | **-0.01276** | **0.005943** | 0.00868 | -0.01746 | -0.03670 | -0.06715 | 0.07381 | 0.06840 | 0.04508 | -0.03055 | 0.04298 | -0.02767 |
| Cardiac arrest: No | **0.03470** | **0.03030** | **0.013832** | 0.03569 | 0.02285 | -0.00173 | 0.02742 | -0.08616 | 0.08041 | 0.04806 | -0.00001 | 0.01502 | -0.00323 |
| Cardiac arrest: Yes | **-0.03470** | **-0.03030** | **-0.024942** | -0.03569 | -0.02285 | 0.00173 | -0.02742 | N.E. | -0.08041 | -0.04806 | 0.00001 | -0.01502 | 0.00323 |
| Arrhythmias: No | **0.06100** | **0.04585** | **0.001182** | 0.05850 | -0.02622 | 0.03862 | 0.02027 | -0.10318 | -0.06197 | 0.00458 | 0.01392 | 0.03640 | 0.03091 |
| Arrhythmias: Yes | **-0.06100** | **-0.04585** | **-0.001182** | -0.05850 | 0.02622 | -0.03862 | -0.02027 | 0.10318 | 0.06197 | -0.00458 | -0.01392 | -0.03640 | -0.03091 |
| Paroxysmal tachycardia: No | **0.03968** | **0.01457** | **-0.005593** | -0.02774 | -0.02930 | 0.04161 | -0.00688 | -0.08196 | -0.01020 | -0.09648 | -0.03210 | 0.06327 | 0.12385 |
| Paroxysmal tachycardia: Yes | **-0.03968** | **-0.01457** | **0.005593** | 0.02774 | 0.02930 | -0.04161 | 0.00688 | 0.08196 | 0.01020 | 0.09648 | 0.03210 | -0.06327 | -0.12385 |
| Ventricular tachycardia: No | **0.05381** | **0.03755** | **0.011982** | 0.11662 | -0.00138 | 0.02423 | -0.02021 | -0.11311 | 0.06370 | -0.08596 | 0.03798 | -0.01853 | 0.11648 |
| Ventricular tachycardia: Yes | **-0.05381** | **-0.03755** | **-0.025881** | -0.11662 | 0.00138 | -0.02423 | 0.02021 | N.E. | -0.06370 | 0.08596 | -0.03798 | 0.01853 | -0.11648 |
| Supraventricular tachycardia and unspecified: No | **0.01568** | **0.00055** | **-0.004336** | -0.03639 | -0.04340 | 0.08724 | -0.02124 | -0.02081 | 0.00145 | -0.03169 | -0.03875 | 0.00316 | 0.05707 |
| Supraventricular tachycardia and unspecified: Yes | **-0.01568** | **-0.00055** | **0.004336** | 0.03639 | 0.04340 | -0.08724 | 0.02124 | 0.02081 | -0.00145 | 0.03169 | 0.03875 | -0.00316 | -0.05707 |
| Atrial fibrillation and flutter: No | **0.06193** | **0.04749** | **0.001312** | 0.06080 | -0.02131 | -0.02692 | 0.03253 | -0.03800 | -0.04605 | -0.00333 | 0.01550 | 0.01307 | 0.02684 |
| Atrial fibrillation and flutter: Yes | **-0.06193** | **-0.04749** | **-0.001312** | -0.06080 | 0.02131 | 0.02692 | -0.03253 | 0.03800 | 0.04605 | 0.00333 | -0.01550 | -0.01307 | -0.02684 |
| Other cardiac arrhythmias: No | **0.01541** | **0.01491** | **0.006304** | 0.06023 | -0.03986 | 0.17576 | -0.02066 | -0.13740 | -0.01923 | -0.08372 | 0.05736 | 0.05082 | 0.01973 |
| Other cardiac arrhythmias: Yes | **-0.01541** | **-0.01491** | **-0.006304** | -0.06023 | 0.03986 | -0.17576 | 0.02066 | 0.13740 | 0.01923 | 0.08372 | -0.05736 | -0.05082 | -0.01973 |
| Other cardiac arrhythmias (subgroup): No | **0.01427** | **0.01039** | **0.002041** | 0.03269 | -0.04801 | 0.15877 | -0.03322 | -0.10765 | -0.02680 | -0.08448 | 0.07090 | 0.04836 | 0.00985 |
| Other cardiac arrhythmias (subgroup): Yes | **-0.01427** | **-0.01039** | **-0.002041** | -0.03269 | 0.04801 | -0.15877 | 0.03322 | 0.10765 | 0.02680 | 0.08448 | -0.07090 | -0.04836 | -0.00985 |
| Heart failure: No | **0.04591** | **0.05028** | **0.017668** | 0.01236 | -0.03408 | -0.01672 | 0.02524 | -0.04122 | 0.10233 | -0.10371 | 0.12053 | -0.01272 | 0.12468 |
| Heart failure: Yes | **-0.04591** | **-0.05028** | **-0.017668** | -0.01236 | 0.03408 | 0.01672 | -0.02524 | 0.04122 | -0.10233 | 0.10371 | -0.12053 | 0.01272 | -0.12468 |
| Cerebrovascular disease: No | **0.07674** | **0.05536** | **0.007933** | -0.02725 | -0.04448 | -0.03914 | -0.06754 | -0.01780 | 0.00818 | 0.04323 | 0.10354 | 0.06339 | 0.05721 |
| Cerebrovascular disease: Yes | **-0.07674** | **-0.05536** | **-0.007933** | 0.02725 | 0.04448 | 0.03914 | 0.06754 | 0.01780 | -0.00818 | -0.04323 | -0.10354 | -0.06339 | -0.05721 |
| Cerebral haemorrhage (subarachnoid, intracerebral, other non-traumatic): No | **0.06592** | **0.02671** | **-0.005447** | -0.03710 | 0.03968 | -0.01083 | -0.03719 | -0.02152 | 0.03647 | -0.04580 | 0.03994 | 0.08518 | -0.10330 |
| Cerebral haemorrhage (subarachnoid, intracerebral, other non-traumatic): Yes | **-0.06592** | **-0.02671** | **0.005447** | 0.03710 | -0.03968 | 0.01083 | 0.03719 | 0.02152 | -0.03647 | 0.04580 | -0.03994 | -0.08518 | 0.10330 |
| Cerebral infarction and stroke: No | **0.08019** | **0.05750** | **0.007758** | -0.03283 | -0.04490 | -0.05131 | -0.10129 | -0.06166 | -0.00891 | 0.06193 | 0.09764 | 0.05899 | 0.15993 |
| Cerebral infarction and stroke: Yes | **-0.08019** | **-0.05750** | **-0.007758** | 0.03283 | 0.04490 | 0.05131 | 0.10129 | 0.06166 | 0.00891 | -0.06193 | -0.09764 | -0.05899 | -0.15993 |
| Transient ischaemic attack: No | **0.03258** | **0.02349** | **0.001408** | -0.00483 | -0.02605 | 0.01930 | -0.08671 | 0.07914 | 0.02623 | -0.03274 | -0.02511 | 0.03800 | 0.02684 |
| Transient ischaemic attack: Yes | **-0.03258** | **-0.02349** | **-0.001408** | 0.00483 | 0.02605 | -0.01930 | 0.08671 | -0.07914 | -0.02623 | 0.03274 | 0.02511 | -0.03800 | -0.02684 |
| Other cerebrovascular disease and sequelae of cerebrovascular disease: No | **0.05960** | **0.05368** | **0.016676** | -0.01455 | 0.00774 | -0.04542 | -0.02836 | 0.01620 | 0.03057 | 0.04976 | 0.12774 | -0.01083 | 0.03391 |
| Other cerebrovascular disease and sequelae of cerebrovascular disease: Yes | **-0.05960** | **-0.05368** | **-0.016676** | 0.01455 | -0.00774 | 0.04542 | 0.02836 | -0.01620 | -0.03057 | -0.04976 | -0.12774 | 0.01083 | -0.03391 |
| Hypertension and hypertensive heart disease: No | **-0.01547** | **0.00324** | **0.011339** | -0.01182 | -0.05545 | -0.07244 | -0.03141 | 0.05554 | -0.05718 | -0.03147 | 0.11452 | 0.09686 | 0.10624 |
| Hypertension and hypertensive heart disease: Yes | **0.01547** | **-0.00324** | **-0.011339** | 0.01182 | 0.05545 | 0.07244 | 0.03141 | -0.05554 | 0.05718 | 0.03147 | -0.11452 | -0.09686 | -0.10624 |
| Diseases of arteries, arterioles, and capillaries: No | **-0.01966** | **-0.00325** | **-0.007315** | 0.02850 | 0.09258 | 0.00379 | -0.11993 | -0.00404 | -0.07610 | 0.04517 | 0.02224 | -0.05831 | -0.00705 |
| Diseases of arteries, arterioles, and capillaries: Yes | **0.01966** | **0.00325** | **0.007315** | -0.02850 | -0.09258 | -0.00379 | 0.11993 | 0.00404 | 0.07610 | -0.04517 | -0.02224 | 0.05831 | 0.00705 |
| Peripheral arterial revascularisation procedures: No | **0.00458** | **0.01816** | **0.019401** | 0.01096 | 0.04066 | -0.04778 | -0.10435 | 0.08402 | 0.00079 | 0.03398 | 0.31368 | -0.04449 | -0.09345 |
| Peripheral arterial revascularisation procedures: Yes | **-0.00458** | **-0.01816** | **-0.019401** | -0.01096 | -0.04066 | 0.04778 | 0.10435 | -0.08402 | -0.00079 | -0.03398 | -0.31368 | 0.04449 | 0.09345 |
| Other form of heart diseases: No | **0.06013** | **0.05925** | **0.019293** | 0.05519 | 0.05536 | -0.03003 | -0.06352 | 0.04364 | -0.17587 | 0.08865 | 0.07241 | 0.02946 | 0.11765 |
| Other form of heart diseases: Yes | **-0.06013** | **-0.05925** | **-0.019293** | -0.05519 | -0.05536 | 0.03003 | 0.06352 | -0.04364 | 0.17587 | -0.08865 | -0.07241 | -0.02946 | -0.11765 |
| Hyperlipidaemia: No | **0.02178** | **0.01421** | **-0.004381** | -0.03005 | -0.08298 | -0.00121 | 0.02538 | -0.02994 | -0.11646 | -0.01909 | 0.14561 | -0.02538 | 0.09031 |
| Hyperlipidaemia: Yes | **-0.02178** | **-0.01421** | **0.004381** | 0.03005 | 0.08298 | 0.00121 | -0.02538 | 0.02994 | 0.11646 | 0.01909 | -0.14561 | 0.02538 | -0.09031 |
| Diabetes mellitus: No | **0.04802** | **0.04695** | **0.013226** | 0.00055 | 0.01556 | 0.02069 | -0.00465 | -0.00092 | -0.06544 | -0.01052 | 0.03340 | 0.06040 | 0.08320 |
| Diabetes mellitus: Yes | **-0.04802** | **-0.04695** | **-0.013226** | -0.00055 | -0.01556 | -0.02069 | 0.00465 | 0.00092 | 0.06544 | 0.01052 | -0.03340 | -0.06040 | -0.08320 |
| Renal disease: No | **0.08448** | **0.06816** | **0.012832** | 0.04213 | 0.05630 | -0.05017 | 0.06662 | -0.08813 | -0.06602 | 0.01397 | 0.01099 | 0.10701 | 0.03561 |
| Renal disease: Yes | **-0.08448** | **-0.06816** | **-0.012832** | -0.04213 | -0.05630 | 0.05017 | -0.06662 | 0.08813 | 0.06602 | -0.01397 | -0.01099 | -0.10701 | -0.03561 |
| Chronic kidney disease: No | **0.02647** | **0.03090** | **0.011108** | 0.05820 | -0.05290 | 0.00336 | -0.06814 | -0.04780 | -0.03475 | 0.11089 | 0.14199 | 0.00817 | -0.00794 |
| Chronic kidney disease: Yes | **-0.02647** | **-0.03090** | **-0.011108** | -0.05820 | 0.05290 | -0.00336 | 0.06814 | 0.04780 | 0.03475 | -0.11089 | -0.14199 | -0.00817 | 0.00794 |
| Other renal disorders: No | **0.08796** | **0.06518** | **0.011759** | 0.03494 | 0.04759 | -0.05947 | 0.05529 | -0.15525 | -0.05051 | 0.03036 | 0.00343 | 0.15489 | 0.05633 |
| Other renal disorders: Yes | **-0.08796** | **-0.06518** | **-0.011759** | -0.03494 | -0.04759 | 0.05947 | -0.05529 | 0.15525 | 0.05051 | -0.03036 | -0.00343 | -0.15489 | -0.05633 |
| Anaemias: No | **0.06928** | **0.06488** | **0.021182** | 0.07796 | -0.02999 | 0.04616 | -0.07398 | -0.00687 | -0.04928 | 0.00192 | -0.00322 | 0.11917 | 0.12994 |
| Anaemias: Yes | **-0.06928** | **-0.06488** | **-0.021182** | -0.07796 | 0.02999 | -0.04616 | 0.07398 | 0.00687 | 0.04928 | -0.00192 | 0.00322 | -0.11917 | -0.12994 |
| Nutritional anaemias: No | **0.05276** | **0.05284** | **0.016560** | 0.04603 | 0.03227 | -0.00256 | 0.02404 | 0.00876 | -0.06044 | 0.02005 | -0.03805 | 0.08942 | 0.04608 |
| Nutritional anaemias: Yes | **-0.05276** | **-0.05284** | **-0.016560** | -0.04603 | -0.03227 | 0.00256 | -0.02404 | -0.00876 | 0.06044 | -0.02005 | 0.03805 | -0.08942 | -0.04608 |
| Iron deficiency anaemias: No | **0.04522** | **0.04431** | **0.012170** | 0.04293 | 0.04379 | -0.02854 | 0.02612 | -0.01259 | -0.02351 | 0.01978 | -0.04395 | 0.00566 | 0.09199 |
| Iron deficiency anaemias: Yes | **-0.04522** | **-0.04431** | **-0.012170** | -0.04293 | -0.04379 | 0.02854 | -0.02612 | 0.01259 | 0.02351 | -0.01978 | 0.04395 | -0.00566 | -0.09199 |
| Other anaemias: No | **0.06122** | **0.05626** | **0.019868** | 0.03913 | -0.05412 | 0.06496 | -0.08557 | -0.01560 | -0.02108 | 0.01490 | -0.03032 | 0.12346 | 0.16291 |
| Other anaemias: Yes | **-0.06122** | **-0.05626** | **-0.019868** | -0.03913 | 0.05412 | -0.06496 | 0.08557 | 0.01560 | 0.02108 | -0.01490 | 0.03032 | -0.12346 | -0.16291 |
| Peptic ulcer disease: No | **0.02446** | **0.02392** | **-0.001529** | -0.02057 | -0.00035 | 0.00908 | 0.01731 | -0.06074 | -0.03120 | 0.08016 | -0.00815 | -0.04854 | 0.04770 |
| Peptic ulcer disease: Yes | **-0.02446** | **-0.02392** | **0.001529** | 0.02057 | 0.00035 | -0.00908 | -0.01731 | 0.06074 | 0.03120 | -0.08016 | 0.00815 | 0.04854 | -0.04770 |
| Liver disease: No | **0.02844** | **0.01280** | **0.002683** | -0.03887 | -0.10503 | -0.01674 | 0.02477 | -0.01662 | -0.00328 | 0.04874 | 0.04826 | -0.07465 | 0.16027 |
| Liver disease: Yes | **-0.02844** | **-0.01280** | **-0.002683** | 0.03887 | 0.10503 | 0.01674 | -0.02477 | 0.01662 | 0.00328 | -0.04874 | -0.04826 | 0.07465 | -0.16027 |
| Osteoporosis: No | **0.08451** | **0.05213** | **0.006503** | -0.03821 | -0.00567 | -0.00969 | -0.04990 | 0.01392 | -0.03947 | -0.05408 | 0.10983 | 0.13556 | 0.00274 |
| Osteoporosis: Yes | **-0.08451** | **-0.05213** | **-0.006503** | 0.03821 | 0.00567 | 0.00969 | 0.04990 | -0.01392 | 0.03947 | 0.05408 | -0.10983 | -0.13556 | -0.00274 |
| Rheumatoid arthritis and other inflammatory arthropathies: No | **-0.00241** | **0.00870** | **0.009043** | 0.05099 | -0.04192 | 0.06785 | 0.07960 | 0.06727 | -0.03179 | -0.02231 | -0.06195 | -0.08899 | 0.07167 |
| Rheumatoid arthritis and other inflammatory arthropathies: Yes | **0.00241** | **-0.00870** | **-0.009043** | -0.05099 | 0.04192 | -0.06785 | -0.07960 | -0.06727 | 0.03179 | 0.02231 | 0.06195 | 0.08899 | -0.07167 |
| Systemic connective tissue diseases: No | **-0.02776** | **-0.01740** | **-0.004023** | 0.04861 | 0.00469 | -0.08278 | -0.05400 | -0.00280 | -0.04305 | -0.04233 | 0.06842 | 0.08040 | -0.01741 |
| Systemic connective tissue diseases: Yes | **0.02776** | **0.01740** | **0.004023** | -0.04861 | -0.00469 | 0.08278 | 0.05400 | 0.00280 | 0.04305 | 0.04233 | -0.06842 | -0.08040 | 0.01741 |
| Malignancy: No | **0.03230** | **0.03749** | **0.016237** | 0.04469 | -0.00718 | -0.01612 | 0.02531 | 0.16187 | -0.07859 | -0.02563 | -0.10111 | 0.16346 | -0.00433 |
| Malignancy: Yes | **-0.03230** | **-0.03749** | **-0.016237** | -0.04469 | 0.00718 | 0.01612 | -0.02531 | -0.16187 | 0.07859 | 0.02563 | 0.10111 | -0.16346 | 0.00433 |
| Depressive disorders: No | **0.02801** | **0.02032** | **-0.000189** | 0.00042 | 0.05523 | -0.07786 | -0.03095 | -0.03119 | -0.01238 | 0.02010 | 0.07694 | 0.02804 | -0.03023 |
| Depressive disorders: Yes | **-0.02801** | **-0.02032** | **0.000189** | -0.00042 | -0.05523 | 0.07786 | 0.03095 | 0.03119 | 0.01238 | -0.02010 | -0.07694 | -0.02804 | 0.03023 |
| Asthma: No | **0.02876** | **0.01324** | **0.003614** | 0.01112 | 0.01667 | -0.00182 | 0.09285 | -0.07576 | -0.04660 | -0.05765 | 0.16699 | 0.03814 | -0.10781 |
| Asthma: Yes | **-0.02876** | **-0.01324** | **-0.003614** | -0.01112 | -0.01667 | 0.00182 | -0.09285 | 0.07576 | 0.04660 | 0.05765 | -0.16699 | -0.03814 | 0.10781 |
| Respiratory medications: No | **0.21441** | **0.17241** | **0.041202** | 0.04649 | 0.03678 | 0.02775 | 0.01538 | 0.04839 | 0.04248 | 0.04872 | -0.00259 | 0.09987 | 0.04874 |
| Respiratory medications: Yes | **-0.21441** | **-0.17241** | **-0.041202** | -0.04649 | -0.03678 | -0.02775 | -0.01538 | -0.04839 | -0.04248 | -0.04872 | 0.00259 | -0.09987 | -0.04874 |
| Inhaled short-acting muscarinic antagonists (SAMAs): No | **-0.00309** | **0.00407** | **-0.004381** | 0.01109 | -0.00138 | -0.04514 | 0.01529 | -0.06164 | N.E. | N.E. | 0.09142 | -0.04030 | N.E. |
| Inhaled short-acting muscarinic antagonists (SAMAs): Yes | **0.00309** | **-0.00407** | **0.004381** | -0.01109 | 0.00138 | 0.04514 | -0.01529 | 0.06164 | N.E. | N.E. | -0.09142 | 0.04030 | N.E. |
| Inhaled long-acting muscarinic antagonists (LAMAs): No | **0.22591** | **0.15968** | **0.004059** | 0.16086 | -0.00442 | 0.13300 | 0.07154 | 0.00860 | -0.11699 | -0.06156 | -0.11397 | 0.05107 | -0.08755 |
| Inhaled long-acting muscarinic antagonists (LAMAs): Yes | **-0.22591** | **-0.15968** | **-0.004059** | -0.16086 | 0.00442 | -0.13300 | -0.07154 | -0.00860 | 0.11699 | 0.06156 | 0.11397 | -0.05107 | 0.08755 |
| Inhaled short-acting beta2-agonists (SABAs): No | **0.11962** | **0.09369** | **0.009906** | 0.04719 | 0.00262 | -0.07592 | -0.04509 | 0.04186 | 0.03245 | -0.01108 | 0.04285 | 0.04247 | 0.02171 |
| Inhaled short-acting beta2-agonists (SABAs): Yes | **-0.11962** | **-0.09369** | **-0.009906** | -0.04719 | -0.00262 | 0.07592 | 0.04509 | -0.04186 | -0.03245 | 0.01108 | -0.04285 | -0.04247 | -0.02171 |
| Inhaled glucocorticosteroids (ICS): No | **0.07449** | **0.03611** | **0.002057** | 0.03314 | 0.00904 | -0.09420 | 0.00071 | 0.00618 | -0.03003 | -0.01469 | 0.14508 | -0.01770 | -0.01696 |
| Inhaled glucocorticosteroids (ICS): Yes | **-0.07449** | **-0.03611** | **-0.002057** | -0.03314 | -0.00904 | 0.09420 | -0.00071 | -0.00618 | 0.03003 | 0.01469 | -0.14508 | 0.01770 | 0.01696 |
| Fixed combinations of SABA and SAMA: No | **0.19511** | **0.11084** | **0.012610** | N.E. | -0.02223 | -0.06658 | -0.03835 | -0.02521 | -0.02813 | 0.13422 | 0.05700 | -0.03651 | 0.13926 |
| Fixed combinations of SABA and SAMA: Yes | **-0.19511** | **-0.11084** | **-0.012610** | N.E. | 0.02223 | 0.06658 | 0.03835 | 0.02521 | 0.02813 | -0.13422 | -0.05700 | 0.03651 | -0.13926 |
| Systemic glucocorticosteroids: No | **0.12234** | **0.08326** | **-0.003250** | -0.04388 | 0.05395 | -0.03122 | 0.01994 | -0.01941 | 0.01033 | -0.00489 | -0.06039 | 0.00991 | 0.03316 |
| Systemic glucocorticosteroids: Yes | **-0.12234** | **-0.08326** | **0.003250** | 0.04388 | -0.05395 | 0.03122 | -0.01994 | 0.01941 | -0.01033 | 0.00489 | 0.06039 | -0.00991 | -0.03316 |
| Systemic beta2-agonists: No | **0.02911** | **0.01157** | **-0.009211** | N.E. | 0.00380 | 0.00636 | -0.04270 | -0.05137 | 0.03669 | -0.07332 | -0.02060 | -0.04030 | 0.09854 |
| Systemic beta2-agonists: Yes | **-0.02911** | **-0.01157** | **-0.014081** | N.E. | -0.00380 | -0.00636 | N.E. | N.E. | -0.03669 | N.E. | 0.02060 | 0.04030 | -0.09854 |
| Xanthines and adrenergics: No | **0.02763** | **0.00669** | **-0.001046** | 0.09729 | N.E. | -0.02752 | -0.02622 | -0.07850 | -0.06762 | N.E. | 0.06267 | -0.01184 | 0.04337 |
| Xanthines and adrenergics: Yes | **-0.02763** | **-0.00669** | **-0.002736** | -0.09729 | N.E. | N.E. | 0.02622 | 0.07850 | 0.06762 | N.E. | -0.06267 | 0.01184 | -0.04337 |
| Roflumilast: No | **0.09878** | N.E**.** | N.E. | N.E. | N.E. | N.E. | N.E. | N.E. | N.E. | N.E. | N.E. | N.E. | N.E. |
| Roflumilast: Yes | **-0.09878** | N.E**.** | N.E. | N.E. | N.E. | N.E. | N.E. | N.E. | N.E. | N.E. | N.E. | N.E. | N.E. |
| Nasal glucocorticosteroids: No | **-0.02280** | **-0.01904** | **-0.007661** | 0.06868 | 0.00884 | 0.02334 | -0.01106 | -0.02534 | -0.03143 | -0.01061 | -0.00532 | -0.00661 | -0.08710 |
| Nasal glucocorticosteroids: Yes | **0.02280** | **0.01904** | **0.007661** | -0.06868 | -0.00884 | -0.02334 | 0.01106 | 0.02534 | 0.03143 | 0.01061 | 0.00532 | 0.00661 | 0.08710 |
| Leukotriene receptor antagonists: No | **0.01846** | **0.00765** | **0.017772** | 0.01359 | -0.01294 | -0.03641 | 0.21139 | 0.00243 | 0.08885 | -0.01197 | 0.09987 | -0.06950 | -0.10758 |
| Leukotriene receptor antagonists: Yes | **-0.01846** | **-0.00765** | **-0.017772** | -0.01359 | 0.01294 | 0.03641 | -0.21139 | -0.00243 | -0.08885 | 0.01197 | -0.09987 | 0.06950 | 0.10758 |
| Oxygen therapy: No | **0.14577** | **0.11180** | **0.003018** | -0.03102 | 0.01884 | -0.01725 | 0.03968 | -0.08187 | -0.00141 | 0.01912 | 0.08266 | -0.12194 | 0.12336 |
| Oxygen therapy: Yes | **-0.14577** | **-0.11180** | **-0.003018** | 0.03102 | -0.01884 | 0.01725 | -0.03968 | 0.08187 | 0.00141 | -0.01912 | -0.08266 | 0.12194 | -0.12336 |
| Nebuliser therapy: No | **0.03142** | **0.02388** | **0.003059** | N.E**.** | -0.02451 | N.E. | N.E. | N.E. | N.E. | N.E. | N.E. | N.E. | 0.03063 |
| Nebuliser therapy: Yes | **-0.03142** | **-0.02388** | **-0.030632** | N.E**.** | N.E. | N.E. | N.E. | N.E. | N.E. | N.E. | N.E. | N.E. | -0.03063 |
| Cardiovascular medications: No | **0.03211** | **0.03384** | **0.002238** | -0.01639 | -0.03400 | 0.03695 | 0.01061 | 0.02346 | -0.11616 | -0.05309 | 0.06061 | 0.03301 | 0.07740 |
| Cardiovascular medications: Yes | **-0.03211** | **-0.03384** | **-0.002238** | 0.01639 | 0.03400 | -0.03695 | -0.01061 | -0.02346 | 0.11616 | 0.05309 | -0.06061 | -0.03301 | -0.07740 |
| Cardiac glycosides and antiarrhythmics, Class I and III: No | **0.07909** | **0.05615** | **0.008535** | 0.04131 | -0.03314 | -0.00241 | 0.11965 | -0.06229 | -0.07510 | 0.06851 | -0.03100 | -0.01632 | 0.07615 |
| Cardiac glycosides and antiarrhythmics, Class I and III: Yes | **-0.07909** | **-0.05615** | **-0.008535** | -0.04131 | 0.03314 | 0.00241 | -0.11965 | 0.06229 | 0.07510 | -0.06851 | 0.03100 | 0.01632 | -0.07615 |
| Vasodilators used in cardiac diseases: No | **0.00439** | **0.01012** | **-0.000386** | 0.00935 | -0.03273 | -0.01420 | 0.01579 | 0.04730 | -0.05462 | -0.08744 | 0.02541 | 0.13937 | -0.05209 |
| Vasodilators used in cardiac diseases: Yes | **-0.00439** | **-0.01012** | **0.000386** | -0.00935 | 0.03273 | 0.01420 | -0.01579 | -0.04730 | 0.05462 | 0.08744 | -0.02541 | -0.13937 | 0.05209 |
| Cardiac stimulants and other cardiac preparations: No | **0.00393** | **0.00236** | **0.001835** | 0.04405 | -0.01856 | 0.05165 | -0.00888 | 0.02459 | 0.01915 | -0.04910 | . | -0.02166 | -0.02471 |
| Cardiac stimulants and other cardiac preparations: Yes | **-0.00393** | **-0.00236** | **-0.001835** | -0.04405 | 0.01856 | -0.05165 | 0.00888 | -0.02459 | -0.01915 | 0.04910 | . | 0.02166 | 0.02471 |
| Diuretics: No | **0.10655** | **0.08085** | **0.014803** | -0.06057 | -0.07908 | -0.08985 | -0.05206 | 0.09331 | -0.02937 | -0.03675 | 0.19288 | 0.00079 | 0.20871 |
| Diuretics: Yes | **-0.10655** | **-0.08085** | **-0.014803** | 0.06057 | 0.07908 | 0.08985 | 0.05206 | -0.09331 | 0.02937 | 0.03675 | -0.19288 | -0.00079 | -0.20871 |
| Peripheral vasodilators: No | **-0.01548** | N.E**.** | N.E. | N.E. | N.E. | N.E. | N.E. | N.E. | N.E. | N.E. | N.E. | N.E. | N.E. |
| Peripheral vasodilators: Yes | N.E**.** | N.E. | N.E. | N.E. | N.E. | N.E. | N.E. | N.E. | N.E. | N.E. | N.E. | N.E. | N.E**.** |
| Vasoprotective agents: No | **0.00178** | **-0.00489** | **-0.010621** | 0.05870 | -0.09940 | 0.02914 | 0.08373 | -0.07433 | -0.11128 | 0.04228 | 0.02124 | -0.01597 | -0.04032 |
| Vasoprotective agents: Yes | **-0.00178** | **0.00489** | **0.010621** | -0.05870 | 0.09940 | -0.02914 | -0.08373 | 0.07433 | 0.11128 | -0.04228 | -0.02124 | 0.01597 | 0.04032 |
| Beta blocking agents: No | **0.00625** | **0.01714** | **0.005276** | -0.00137 | -0.03418 | -0.00038 | 0.00322 | -0.02136 | 0.00388 | 0.00474 | 0.07169 | 0.00578 | 0.02073 |
| Beta blocking agents: Yes | **-0.00625** | **-0.01714** | **-0.005276** | 0.00137 | 0.03418 | 0.00038 | -0.00322 | 0.02136 | -0.00388 | -0.00474 | -0.07169 | -0.00578 | -0.02073 |
| Calcium channel blockers: No | **0.01356** | **0.00584** | **-0.002810** | -0.01984 | -0.06308 | -0.09872 | -0.05447 | 0.05893 | -0.03430 | 0.08253 | -0.01076 | 0.11523 | -0.00361 |
| Calcium channel blockers: Yes | **-0.01356** | **-0.00584** | **0.002810** | 0.01984 | 0.06308 | 0.09872 | 0.05447 | -0.05893 | 0.03430 | -0.08253 | 0.01076 | -0.11523 | 0.00361 |
| Antihypertensives: No | **0.04115** | **0.03819** | **0.011794** | 0.02522 | 0.12653 | -0.07153 | -0.06641 | -0.06145 | -0.10431 | 0.02614 | -0.01768 | 0.07853 | 0.18289 |
| Antihypertensives: Yes | **-0.04115** | **-0.03819** | **-0.011794** | -0.02522 | -0.12653 | 0.07153 | 0.06641 | 0.06145 | 0.10431 | -0.02614 | 0.01768 | -0.07853 | -0.18289 |
| Agents acting on the renin-angiotensin system: No | **-0.03838** | **-0.01368** | **0.005217** | 0.02253 | -0.05485 | -0.03152 | -0.05974 | 0.03383 | -0.02176 | -0.04069 | 0.12623 | 0.03577 | 0.04237 |
| Agents acting on the renin-angiotensin system: Yes | **0.03838** | **0.01368** | **-0.005217** | -0.02253 | 0.05485 | 0.03152 | 0.05974 | -0.03383 | 0.02176 | 0.04069 | -0.12623 | -0.03577 | -0.04237 |
| Angiotensin-converting-enzyme inhibitors: No | **-0.02278** | **-0.00106** | **0.009235** | -0.00768 | -0.01970 | -0.03907 | -0.10046 | 0.08979 | 0.01095 | -0.04664 | 0.14542 | 0.04038 | 0.01935 |
| Angiotensin-converting-enzyme inhibitors: Yes | **0.02278** | **0.00106** | **-0.009235** | 0.00768 | 0.01970 | 0.03907 | 0.10046 | -0.08979 | -0.01095 | 0.04664 | -0.14542 | -0.04038 | -0.01935 |
| Angiotensin II receptor antagonists: No | **-0.03091** | **-0.01837** | **0.000127** | 0.02597 | -0.06553 | 0.00492 | 0.04675 | -0.02884 | -0.03273 | -0.00121 | 0.00322 | 0.01712 | 0.03159 |
| Angiotensin II receptor antagonists: Yes | **0.03091** | **0.01837** | **-0.000127** | -0.02597 | 0.06553 | -0.00492 | -0.04675 | 0.02884 | 0.03273 | 0.00121 | -0.00322 | -0.01712 | -0.03159 |
| Renin-inhibitors: No | **-0.00304** | **-0.01561** | **-0.047749** | N.E**.** | N.E. | N.E. | -0.04775 | N.E**.** | N.E. | N.E. | N.E. | N.E. | N.E. |
| Renin-inhibitors: Yes | **0.00304** | N.E**.** | N.E. | N.E. | N.E. | N.E. | N.E. | N.E. | N.E. | N.E. | N.E. | N.E. | N.E. |
| Lipid-modifying agents: No | **-0.05625** | **-0.02467** | **0.006651** | -0.05283 | -0.05546 | 0.05930 | -0.03195 | 0.02859 | -0.14803 | -0.01951 | 0.09455 | 0.11366 | 0.07820 |
| Lipid-modifying agents: Yes | **0.05625** | **0.02467** | **-0.006651** | 0.05283 | 0.05546 | -0.05930 | 0.03195 | -0.02859 | 0.14803 | 0.01951 | -0.09455 | -0.11366 | -0.07820 |
| HMG-CoA reductase inhibitors (statins): No | **-0.05316** | **-0.02368** | **0.006783** | -0.06327 | -0.05042 | 0.06411 | -0.03527 | 0.01693 | -0.14718 | -0.02087 | 0.09971 | 0.11813 | 0.08596 |
| HMG-CoA reductase inhibitors (statins): Yes | **0.05316** | **0.02368** | **-0.006783** | 0.06327 | 0.05042 | -0.06411 | 0.03527 | -0.01693 | 0.14718 | 0.02087 | -0.09971 | -0.11813 | -0.08596 |
| Other lipid-modifying agents: No | **-0.02402** | **-0.00749** | **0.008992** | 0.04689 | -0.07447 | 0.07349 | -0.02410 | 0.12192 | -0.01967 | -0.04054 | -0.02179 | -0.00075 | 0.02895 |
| Other lipid-modifying agents: Yes | **0.02402** | **0.00749** | **-0.008992** | -0.04689 | 0.07447 | -0.07349 | 0.02410 | -0.12192 | 0.01967 | 0.04054 | 0.02179 | 0.00075 | -0.02895 |
| Antithrombotic agents: No | **0.05946** | **0.05514** | **0.009469** | 0.00845 | 0.00232 | 0.00139 | -0.07164 | -0.10735 | -0.11815 | 0.05615 | 0.13731 | 0.12763 | 0.05860 |
| Antithrombotic agents: Yes | **-0.05946** | **-0.05514** | **-0.009469** | -0.00845 | -0.00232 | -0.00139 | 0.07164 | 0.10735 | 0.11815 | -0.05615 | -0.13731 | -0.12763 | -0.05860 |
| Platelet aggregation inhibitors: No | **0.02229** | **0.02508** | **0.004353** | -0.01950 | 0.00925 | 0.03212 | -0.06952 | -0.06437 | -0.04324 | 0.00047 | 0.12028 | 0.12386 | -0.04581 |
| Platelet aggregation inhibitors: Yes | **-0.02229** | **-0.02508** | **-0.004353** | 0.01950 | -0.00925 | -0.03212 | 0.06952 | 0.06437 | 0.04324 | -0.00047 | -0.12028 | -0.12386 | 0.04581 |
| Systemic antibacterials: No | **0.15199** | **0.12100** | **0.004911** | 0.04472 | 0.06353 | -0.04329 | -0.01021 | 0.06223 | 0.05337 | -0.00529 | -0.09719 | -0.04002 | 0.02126 |
| Systemic antibacterials: Yes | **-0.15199** | **-0.12100** | **-0.004911** | -0.04472 | -0.06353 | 0.04329 | 0.01021 | -0.06223 | -0.05337 | 0.00529 | 0.09719 | 0.04002 | -0.02126 |
| Iron preparations: No | **0.05004** | **0.03174** | **-0.004730** | 0.01147 | -0.04293 | -0.01048 | -0.07516 | 0.00475 | 0.07584 | -0.03814 | 0.10213 | -0.02878 | -0.04600 |
| Iron preparations: Yes | **-0.05004** | **-0.03174** | **0.004730** | -0.01147 | 0.04293 | 0.01048 | 0.07516 | -0.00475 | -0.07584 | 0.03814 | -0.10213 | 0.02878 | 0.04600 |
| Proton pump inhibitors: No | **0.09792** | **0.06337** | **-0.002329** | -0.04955 | -0.02881 | 0.00227 | 0.01063 | -0.05102 | -0.05335 | -0.01479 | 0.00748 | 0.09257 | 0.06128 |
| Proton pump inhibitors: Yes | **-0.09792** | **-0.06337** | **0.002329** | 0.04955 | 0.02881 | -0.00227 | -0.01063 | 0.05102 | 0.05335 | 0.01479 | -0.00748 | -0.09257 | -0.06128 |
| Drugs used in diabetes: No | **0.03588** | **0.03209** | **0.005812** | 0.01800 | 0.00527 | 0.00038 | 0.03616 | 0.02356 | 0.01247 | -0.03020 | -0.01096 | -0.03686 | 0.04031 |
| Drugs used in diabetes: Yes | **-0.03588** | **-0.03209** | **-0.005812** | -0.01800 | -0.00527 | -0.00038 | -0.03616 | -0.02356 | -0.01247 | 0.03020 | 0.01096 | 0.03686 | -0.04031 |
| Insulins: No | **0.05618** | **0.04588** | **0.003047** | 0.00371 | 0.01046 | -0.00267 | 0.03009 | -0.01426 | -0.04009 | 0.03717 | 0.04557 | -0.00245 | -0.03705 |
| Insulins: Yes | **-0.05618** | **-0.04588** | **-0.003047** | -0.00371 | -0.01046 | 0.00267 | -0.03009 | 0.01426 | 0.04009 | -0.03717 | -0.04557 | 0.00245 | 0.03705 |
| Blood glucose–lowering drugs: No | **0.01954** | **0.01880** | **0.005012** | 0.00718 | 0.00904 | 0.00357 | 0.04366 | 0.01724 | 0.05599 | -0.06333 | -0.04272 | -0.03412 | 0.05361 |
| Blood glucose–lowering drugs: Yes | **-0.01954** | **-0.01880** | **-0.005012** | -0.00718 | -0.00904 | -0.00357 | -0.04366 | -0.01724 | -0.05599 | 0.06333 | 0.04272 | 0.03412 | -0.05361 |
| Drugs for musculoskeletal system: No | **-0.02657** | **-0.01106** | **0.010151** | 0.05081 | -0.02276 | -0.05815 | 0.08705 | -0.02502 | -0.01376 | -0.03919 | 0.19532 | -0.09870 | 0.02590 |
| Drugs for musculoskeletal system: Yes | **0.02657** | **0.01106** | **-0.010151** | -0.05081 | 0.02276 | 0.05815 | -0.08705 | 0.02502 | 0.01376 | 0.03919 | -0.19532 | 0.09870 | -0.02590 |
| Anti-inflammatory and antirheumatic products, non-steroids (non-steroidal anti-inflammatory drugs): | **-0.03149** | **-0.01531** | **0.009559** | 0.05750 | -0.02613 | -0.05807 | 0.09246 | -0.03042 | -0.01663 | -0.04390 | 0.17169 | -0.11713 | 0.06623 |
| Anti-inflammatory and antirheumatic products, non-steroids (non-steroidal anti-inflammatory drugs): | **0.03149** | **0.01531** | **-0.009559** | -0.05750 | 0.02613 | 0.05807 | -0.09246 | 0.03042 | 0.01663 | 0.04390 | -0.17169 | 0.11713 | -0.06623 |
| Acetylsalicylic acid (other analgesics and antipyretics): No | **0.02489** | **0.02061** | **0.014263** | -0.05855 | 0.11921 | -0.03730 | -0.04140 | 0.04892 | 0.01217 | 0.02041 | 0.06267 | 0.06575 | -0.04924 |
| Acetylsalicylic acid (other analgesics and antipyretics): Yes | **-0.02489** | **-0.02061** | **-0.022354** | N.E.. | -0.11921 | 0.03730 | 0.04140 | -0.04892 | -0.01217 | -0.02041 | -0.06267 | -0.06575 | 0.04924 |
| Antidepressants: No | **0.07399** | **0.04794** | **0.000664** | 0.02922 | 0.06771 | -0.03078 | -0.04625 | -0.09307 | 0.04393 | -0.10518 | 0.12126 | 0.10500 | -0.08519 |
| Antidepressants: Yes | **-0.07399** | **-0.04794** | **-0.000664** | -0.02922 | -0.06771 | 0.03078 | 0.04625 | 0.09307 | -0.04393 | 0.10518 | -0.12126 | -0.10500 | 0.08519 |
| Selective serotonin reuptake inhibitors: No | **0.05486** | **0.03980** | **0.007774** | 0.03180 | 0.03343 | -0.07105 | -0.04969 | -0.06519 | 0.08360 | -0.00339 | 0.12809 | 0.10563 | -0.11547 |
| Selective serotonin reuptake inhibitors: Yes | **-0.05486** | **-0.03980** | **-0.007774** | -0.03180 | -0.03343 | 0.07105 | 0.04969 | 0.06519 | -0.08360 | 0.00339 | -0.12809 | -0.10563 | 0.11547 |
| Antineoplastic agents: No | **0.02278** | **-0.00234** | **-0.033519** | N.E**.** | N.E. | N.E. | -0.04775 | N.E**.** | N.E. | N.E. | 0.00359 | N.E.. | -0.05640 |
| Antineoplastic agents: Yes | **-0.02278** | **0.00234** | **-0.003595** | N.E**.** | N.E. | N.E. | N.E. | N.E. | N.E. | N.E. | -0.00359 | N.E.. | N.E.. |
| Immunosuppressants: No | **0.00940** | **0.01171** | **0.032948** | 0.06693 | -0.03290 | -0.05785 | 0.28782 | 0.01236 | -0.07441 | -0.09634 | 0.03081 | 0.19314 | -0.00007 |
| Immunosuppressants: Yes | **-0.00940** | **-0.01171** | **-0.032948** | -0.06693 | 0.03290 | 0.05785 | -0.28782 | -0.01236 | 0.07441 | 0.09634 | -0.03081 | -0.19314 | 0.00007 |
| Antivirals for systemic use: No | **-0.02332** | **-0.01599** | **0.018111** | 0.15630 | 0.09098 | -0.07178 | -0.03835 | -0.06145 | -0.06296 | -0.09363 | -0.06967 | 0.14772 | 0.18395 |
| Antivirals for systemic use: Yes | **0.02332** | **0.01599** | **-0.030527** | -0.15630 | -0.09098 | 0.07178 | 0.03835 | 0.06145 | 0.06296 | . | 0.06967 | -0.14772 | -0.18395 |
| Hormone-replacement therapy: Estrogens, progestogens, progestogens and estrogens in combination: No | **-0.05315** | **-0.03695** | **0.004826** | -0.06893 | -0.00909 | -0.00896 | 0.02174 | 0.02011 | -0.04778 | 0.04857 | 0.04290 | -0.03799 | 0.08769 |
| Hormone-replacement therapy: Estrogens, progestogens, progestogens and estrogens in combination: Yes | **0.05315** | **0.03695** | **-0.004826** | 0.06893 | 0.00909 | 0.00896 | -0.02174 | -0.02011 | 0.04778 | -0.04857 | -0.04290 | 0.03799 | -0.08769 |
| Drugs used in nicotine dependence: No | **0.03210** | **0.01831** | **0.003062** | -0.03912 | 0.11197 | -0.02888 | -0.05522 | -0.06228 | -0.00252 | 0.01862 | 0.06464 | -0.04375 | 0.06715 |
| Drugs used in nicotine dependence: Yes | **-0.03210** | **-0.01831** | **-0.003062** | 0.03912 | -0.11197 | 0.02888 | 0.05522 | 0.06228 | 0.00252 | -0.01862 | -0.06464 | 0.04375 | -0.06715 |
| COPD severity: mild | **0.26233** | **0.21260** | **0.011207** | 0.00989 | -0.03778 | -0.00149 | 0.07952 | 0.08784 | -0.00630 | 0.00774 | -0.01491 | -0.02363 | . |
| COPD severity: moderate | **0.07769** | **0.06739** | **-0.008961** | -0.07433 | 0.09665 | -0.05770 | 0.01666 | -0.04032 | 0.11377 | 0.01606 | -0.07793 | -0.10629 | 0.02382 |
| COPD severity: severe | **-0.17459** | **-0.14420** | **0.004427** | 0.07838 | -0.00963 | 0.00711 | -0.03222 | -0.06457 | -0.04293 | -0.00708 | 0.08281 | -0.05147 | 0.08386 |
| COPD severity: very severe | **-0.15313** | **-0.11328** | **-0.005809** | -0.00530 | -0.06167 | 0.05717 | -0.03952 | 0.06709 | -0.05778 | -0.00594 | -0.03588 | 0.10596 | -0.08222 |
| CCI score: 1 | **0.05986** | **0.05805** | **0.01288** | 0.02065 | -0.00367 | -0.04580 | -0.004012 | 0.00572 | -0.00904 | 0.01099 | 0.01296 | 0.14065 | 0.00031 |
| CCI score: 2 | **0.02684** | **0.03006** | **0.02535** | 0.02807 | 0.03341 | 0.01203 | -0.027788 | 0.06603 | -0.01997 | 0.04081 | 0.04548 | -0.01614 | 0.09152 |
| CCI score: 3+ | **-0.07701** | **-0.07847** | **-0.03268** | -0.04346 | -0.02468 | 0.03227 | 0.025240 | -0.05979 | 0.02428 | -0.04152 | -0.04828 | -0.11172 | -0.07910 |
| Hip fracture: No | **0.03273** | **0.02128** | **0.02811** | -0.04423 | -0.05127 | 0.34266 | 0.011017 | -0.07616 | -0.11671 | 0.10814 | 0.06321 | 0.02070 | 0.02376 |
| Hip fracture: Yes | **-0.03273** | **-0.02128** | **-0.03615** | N.E. | 0.05127 | -0.34266 | -0.011017 | 0.07616 | 0.11671 | -0.10814 | -0.06321 | -0.02070 | -0.02376 |
| Lung cancer: No | **0.06887** | **0.06659** | **0.02677** | 0.05706 | 0.04174 | 0.10562 | 0.096630 | 0.01311 | 0.01394 | 0.11877 | -0.04588 | 0.00219 | -0.13547 |
| Lung cancer: Yes | **-0.06887** | **-0.06659** | **-0.02677** | -0.05706 | -0.04174 | -0.10562 | -0.096630 | -0.01311 | -0.01394 | -0.11877 | 0.04588 | -0.00219 | 0.13547 |
| Other markers of bad fall: No | **0.02353** | **0.00765** | **0.00042** | -0.00966 | 0.01501 | -0.05510 | -0.085501 | 0.00026 | 0.01217 | -0.03050 | -0.02610 | 0.10030 | 0.08336 |
| Other markers of bad fall: Yes | **-0.02353** | **-0.00765** | **-0.00659** | 0.00966 | -0.01501 | N.E. | 0.085501 | -0.00026 | -0.01217 | 0.03050 | 0.02610 | -0.10030 | -0.08336 |
| Metastatic cancer: No | **0.04175** | **0.03708** | **0.06612** | 0.54896 | -0.05757 | 0.08549 | 0.046353 | -0.06145 | 0.19341 | -0.02403 | -0.06380 | -0.05445 | 0.04831 |
| Metastatic cancer: Yes | **-0.04175** | **-0.03708** | **-0.07987** | -0.54896 | . | -0.08549 | -0.046353 | 0.06145 | -0.19341 | 0.02403 | 0.06380 | 0.05445 | -0.04831 |
| Pulmonary cachexia: No | **0.02020** | **0.01730** | **0.00810** | 0.00783 | -0.03076 | 0.05123 | -0.036502 | N.E. | 0.07692 | -0.00926 | 0.07248 | -0.09001 | 0.03099 |
| Pulmonary cachexia: Yes | **-0.02020** | **-0.01730** | **-0.00810** | -0.00783 | 0.03076 | -0.05123 | 0.036502 | N.E. | -0.07692 | 0.00926 | -0.07248 | 0.09001 | -0.03099 |
| Right-sided heart failure: No | **0.07428** | **0.07031** | **0.05665** | -0.05855 | -0.02271 | 0.22858 | -0.031132 | -0.04499 | 0.04236 | 0.08410 | 0.10447 | 0.14471 | 0.11970 |
| Right-sided heart failure: Yes | **-0.07428** | **-0.07031** | **-0.06945** | N.E. | 0.02271 | -0.22858 | 0.031132 | 0.04499 | -0.04236 | -0.08410 | -0.10447 | -0.14471 | -0.11970 |
| Number of hospitalisations within 180 days: 0 | **0.34612** | **0.27837** | **-0.00331** | 0.11276 | -0.05930 | 0.01993 | -0.012811 | 0.02378 | 0.11170 | -0.02722 | -0.04412 | -0.06112 | -0.09669 |
| Number of hospitalisations within 180 days: 1 | **-0.17267** | **-0.17049** | **-0.01078** | -0.09332 | 0.10156 | -0.04300 | 0.038846 | -0.03264 | -0.11794 | 0.02013 | 0.06533 | -0.05305 | 0.00631 |
| Number of hospitalisations within 180 days: 2 | **-0.18267** | **-0.12761** | **-0.01573** | -0.05396 | -0.20474 | 0.04538 | -0.049043 | 0.05830 | 0.00200 | 0.03111 | -0.07275 | 0.02575 | 0.06067 |
| Number of hospitalisations within 180 days: 3-4 | **-0.18920** | **-0.11246** | **-0.01163** | -0.09729 | -0.04735 | -0.01274 | 0.011231 | -0.13281 | -0.00145 | -0.01097 | 0.03049 | 0.10729 | 0.03734 |
| Number of hospitalisations within 180 days: 5+ | **-0.08899** | **-0.07070** | **-0.01532** | N.E. | N.E. | 0.02305 | -0.034190 | 0.03893 | 0.01963 | -0.04057 | 0.02229 | -0.02705 | -0.12464 |
| Number of hospitalisations within 365 days: 0 | **0.29085** | **0.23418** | **-0.00121** | -0.00951 | -0.01998 | -0.01088 | 0.030802 | -0.06049 | 0.14690 | 0.01322 | 0.05238 | -0.09144 | -0.06316 |
| Number of hospitalisations within 365 days: 1 | **-0.09594** | **-0.10960** | **-0.03006** | 0.01920 | 0.03277 | 0.03210 | -0.024443 | -0.03961 | -0.18454 | -0.05173 | -0.00608 | -0.08620 | 0.00790 |
| Number of hospitalisations within 365 days: 2 | **-0.10109** | **-0.06786** | **0.02127** | -0.01784 | 0.03141 | 0.00478 | -0.025768 | 0.12106 | 0.05009 | 0.07632 | -0.07734 | 0.03168 | 0.01836 |
| Number of hospitalisations within 365 days: 3-4 | **-0.14782** | **-0.08369** | **0.02535** | 0.00090 | -0.09213 | -0.02481 | 0.002395 | 0.03732 | 0.00909 | 0.04912 | 0.01005 | 0.16480 | 0.09680 |
| Number of hospitalisations within 365 days: 5+ | **-0.18130** | **-0.14767** | **-0.03942** | -0.00134 | 0.00563 | -0.06704 | 0.022452 | -0.04631 | -0.00967 | -0.12310 | 0.02917 | -0.06271 | -0.14125 |
| Number of hospitalisations with COPD within 90 days: 0 | **0.41397** | **0.35681** | **0.12713** | 0.07564 | -0.01991 | 0.13756 | 0.081092 | 0.25602 | 0.24543 | 0.14417 | 0.09494 | 0.09226 | 0.16409 |
| Number of hospitalisations with COPD within 90 days: 1 | **-0.34914** | **-0.32821** | **-0.12758** | -0.07564 | 0.01991 | -0.17082 | -0.066270 | -0.24729 | -0.24086 | -0.13307 | -0.11274 | -0.07484 | -0.17416 |
| Number of hospitalisations with COPD within 90 days: 2+ | **-0.20722** | **-0.11666** | **-0.02468** | N.E. | N.E. | N.E. | -0.065689 | -0.05455 | -0.03015 | -0.03248 | 0.02824 | -0.02962 | 0.01146 |
| Number of hospitalisations with COPD within 180 days: 0 | **0.39596** | **0.32883** | **0.06686** | 0.09353 | -0.02407 | 0.09625 | 0.034995 | 0.20015 | 0.19121 | 0.10952 | 0.02915 | -0.01004 | -0.05206 |
| Number of hospitalisations with COPD within 180 days: 1 | **-0.29499** | **-0.27860** | **-0.06702** | -0.09353 | 0.02407 | -0.12791 | -0.016245 | -0.20206 | -0.20256 | -0.07153 | -0.03368 | 0.01362 | 0.03966 |
| Number of hospitalisations with COPD within 180 days: 2+ | **-0.25394** | **-0.14853** | **-0.00608** | N.E. | N.E. | 0.08184 | -0.070031 | -0.00900 | 0.01612 | -0.07341 | 0.00709 | -0.00578 | 0.00456 |
| Number of COPD exacerbations within 90 days: 0 | **0.28587** | **0.23017** | **0.04457** | -0.01351 | -0.03214 | 0.00585 | 0.025442 | 0.08277 | 0.15684 | 0.12724 | -0.02470 | -0.01466 | 0.13261 |
| Number of COPD exacerbations within 90 days: 1 | **-0.12199** | **-0.11166** | **-0.02244** | 0.00265 | 0.06551 | 0.06841 | 0.010188 | -0.01622 | -0.09723 | -0.10346 | -0.08730 | 0.02122 | -0.08814 |
| Number of COPD exacerbations within 90 days: 2 | **-0.17400** | **-0.14341** | **-0.03821** | 0.02212 | -0.04279 | -0.15277 | -0.049755 | -0.06871 | -0.10136 | -0.02613 | 0.07293 | 0.05803 | -0.09364 |
| Number of COPD exacerbations within 90 days: 3+ | **-0.17847** | **-0.10787** | **-0.01652** | N.E. | -0.19107 | 0.06488 | -0.008024 | -0.08518 | 0.03444 | -0.00277 | 0.07921 | -0.10007 | 0.05993 |
| Number of COPD exacerbations within 180 days: 0 | **0.27064** | **0.21112** | **-0.01418** | -0.03885 | -0.00016 | -0.04280 | -0.025774 | 0.13306 | 0.07344 | 0.06324 | -0.07351 | -0.12533 | -0.10515 |
| Number of COPD exacerbations within 180 days: 1 | **-0.08065** | **-0.07175** | **-0.00018** | 0.00932 | 0.07352 | 0.06453 | 0.052741 | -0.09504 | 0.02745 | -0.03366 | -0.09718 | 0.01175 | -0.01522 |
| Number of COPD exacerbations within 180 days: 2 | **-0.08235** | **-0.08414** | **-0.00499** | 0.03436 | -0.02548 | -0.04146 | -0.066564 | -0.05498 | -0.10447 | 0.00063 | 0.08471 | 0.05792 | 0.06544 |
| Number of COPD exacerbations within 180 days: 3+ | **-0.24158** | **-0.15002** | **-0.00319** | 0.06695 | -0.26296 | 0.04090 | 0.051361 | 0.01132 | -0.01425 | -0.03243 | 0.08659 | 0.01279 | 0.00783 |

CCI = Charlson Comorbidity Index; COPD = chronic obstructive pulmonary disease; HMG-CoA = hydroxymethylglutaryl-coenzyme A; ICS = inhaled glucocorticosteroid; LABA = long-acting beta2-agonist; LAMA = long-acting muscarinic antagonist; NE = not estimable; SABA = short-acting beta2-agonist; SAMA = short-acting muscarinic antagonist; STdiff Dx = standardised difference for each propensity score decile.

Note on colour codes:

ORANGE: Cells in orange indicate standardised bias ≥ 0.1 and < 0.2.

YELLOW: Cells in yellow indicate standardised bias ≥ 0.2.

Table S14. Patient Demographics, Clinical Characteristics, and Medications at the Index Date in Each Study Cohort After Trimming and Matching Stratified by PS Decile Groups, Restricting the Population to Users of LABA/LAMA Who Are LABA Naive and Have No Hospitalisations for COPD in the Last 90 Days

|  | **Cohort** | | | | | | | | | | | | | | | | | | | | | | | |
| --- | --- | --- | --- | --- | --- | --- | --- | --- | --- | --- | --- | --- | --- | --- | --- | --- | --- | --- | --- | --- | --- | --- | --- | --- |
|  | **Olodaterol cohort** | | | | | | | | | | | | **Other LABA cohort** | | | | | | | | | | | |
|  | **Overall** | | **PS decile** | | | | | | | | | | **Overall** | | **PS decile** | | | | | | | | | |
|  |  |  | **1** | **2** | **3** | **4** | **5** | **6** | **7** | **8** | **9** | **10** |  |  | **1** | **2** | **3** | **4** | **5** | **6** | **7** | **8** | **9** | **10** |
|  | **N** | **%** | **%** | **%** | **%** | **%** | **%** | **%** | **%** | **%** | **%** | **%** | **N** | **%** | **%** | **%** | **%** | **%** | **%** | **%** | **%** | **%** | **%** | **%** |
| Total | **3,843** | **100** | 100 | 100 | 100 | 100 | 100 | 100 | 100 | 100 | 100 | 100 | **14,029** | **100** | 100 | 100 | 100 | 100 | 100 | 100 | 100 | 100 | 100 | 100 |
| **Age (years)** | **3,843** |  | 385 | 384 | 383 | 386 | 383 | 385 | 385 | 383 | 384 | 385 | **14,029** |  | 2,369 | 2,134 | 1,791 | 1,591 | 1,452 | 1,215 | 1,052 | 962 | 808 | 655 |
| Mean (SD) | **71.5 (10.2)** |  | 72.9 (9.4) | 70.6 (10.3) | 70.6 (10.1) | 70.6 (10.7) | 71.2 (10.2) | 72.3 (9.8) | 71.8 (10.3) | 71.8 (10.2) | 72.1 (10.2) | 71.5 (10.3) | **71.5 (9.9)** |  | 72.3 (8.8) | 70.6 (10.4) | 70.4 (10.1) | 70.8 (10.5) | 71.6 (10.5) | 72.6 (9.7) | 72.4 (9.7) | 72..1 (9.6) | 72.0 (9.9) | 70.7 (10.5) |
| Median (Q1, Q3) | **72 (65, 59)** |  | 74 (66, 79) | 71 (64, 79) | 72 (63, 78) | 71 (64, 78) | 72 (64, 79) | 73 (66, 80) | 72 (66, 80) | 72 (66, 79) | 73 (65, 80) | 72 (65, 80) | **72 (65, 79)** |  | 73 (66, 79) | 72 (63, 78) | 71 (63, 78) | 71 (64, 79) | 72 (66, 79) | 73 (66, 80) | 73 (66, 80) | 73 (66, 79) | 72 (66, 79) | 72 (63, 79) |
| Min, Max (rounded to 10 years) | **40, 100** |  | 40, 90 | 40, 90 | 40, 90 | 40, 90 | 40, 90 | 40, 100 | 40, 90 | 40, 90 | 40, 100 | 40, 100 | **40, 100** |  | 50, 100 | 40, 100 | 40, 90 | 40, 90 | 40, 90 | 40, 90 | 40, 90 | 40, 90 | 50, 90 | 40, 90 |
| **Age group (years)** |  |  |  |  |  |  |  |  |  |  |  |  |  |  |  |  |  |  |  |  |  |  |  |  |
| 40-60 | **581** | **15** | 9 | 16 | 20 | 18 | 16 | 12 | 14 | 15 | 15 | 16 | **2,119** | **15** | 9 | 18 | 19 | 18 | 16 | 12 | 12 | 14 | 17 | 20 |
| 61-74 | **1,709** | **44** | 44 | 47 | 42 | 46 | 44 | 44 | 44 | 45 | 44 | 44 | **6,375** | **45** | 48 | 46 | 46 | 44 | 43 | 44 | 47 | 45 | 43 | 41 |
| 75-84 | **1,209** | **31** | 36 | 28 | 31 | 28 | 32 | 36 | 32 | 31 | 30 | 30 | **4,322** | **31** | 36 | 28 | 28 | 30 | 30 | 33 | 31 | 32 | 29 | 32 |
| 85+ | **344** | **9** | 10 | 8 | 7 | 8 | 8 | 8 | 10 | 9 | 11 | 9 | **1,213** | **9** | 7 | 8 | 7 | 8 | 11 | 11 | 10 | 9 | 11 | 7 |
| **Gender** |  |  |  |  |  |  |  |  |  |  |  |  |  |  |  |  |  |  |  |  |  |  |  |  |
| Female | **1,850** | **48** | 45 | 47 | 54 | 51 | 48 | 43 | 45 | 49 | 48 | 50 | **6,721** | **48** | 48 | 43 | 48 | 48 | 50 | 47 | 47 | 51 | 51 | 52 |
| Male | **1,993** | **52** | 55 | 53 | 46 | 49 | 52 | 57 | 55 | 51 | 52 | 50 | **7,308** | **52** | 52 | 57 | 52 | 52 | 50 | 53 | 53 | 49 | 49 | 48 |
| **Calendar year at index date** |  |  |  |  |  |  |  |  |  |  |  |  |  |  |  |  |  |  |  |  |  |  |  |  |
| 2015 | **233** | **6** | 4 | 6 | 5 | 6 | 8 | 9 | 9 | 5 | 5 | 4 | **833** | **6** | 6 | 5 | 6 | 5 | 6 | 7 | 5 | 5 | 8 | 7 |
| 2016 | **817** | **21** | 23 | 19 | 20 | 21 | 20 | 22 | 20 | 22 | 22 | 24 | **2,962** | **21** | 24 | 22 | 19 | 19 | 21 | 19 | 20 | 23 | 23 | 22 |
| 2017 | **1,254** | **33** | 35 | 30 | 36 | 33 | 33 | 31 | 34 | 31 | 34 | 30 | **4,626** | **33** | 32 | 32 | 37 | 33 | 31 | 33 | 32 | 37 | 33 | 27 |
| 2018 | **1,470** | **38** | 38 | 45 | 38 | 40 | 38 | 38 | 35 | 39 | 35 | 36 | **5,472** | **39** | 38 | 40 | 37 | 43 | 40 | 40 | 41 | 33 | 34 | 40 |
| 2019 | **69** | **2** | 0 | 0 | 1 | 0 | 1 | 1 | 3 | 3 | 3 | 6 | **136** | **1** | 0 | 0 | 0 | 0 | 1 | 1 | 2 | 2 | 2 | 4 |
| Cardiovascular diseases | **2,849** | **74** | 66 | 69 | 72 | 73 | 77 | 74 | 72 | 73 | 80 | 86 | **10,289** | **73** | 73 | 66 | 73 | 73 | 78 | 76 | 72 | 75 | 78 | 80 |
| Ischaemic heart disease | **1,145** | **30** | 29 | 24 | 27 | 30 | 30 | 32 | 28 | 28 | 34 | 36 | **4,196** | **30** | 31 | 27 | 31 | 28 | 33 | 37 | 26 | 27 | 31 | 27 |
| Angina pectoris | **867** | **23** | 23 | 20 | 21 | 22 | 19 | 25 | 20 | 22 | 28 | 26 | **3,165** | **23** | 23 | 21 | 22 | 20 | 25 | 26 | 20 | 23 | 25 | 20 |
| Acute myocardial infarction | **395** | **10** | 10 | 8 | 9 | 10 | 11 | 12 | 10 | 9 | 13 | 12 | **1,464** | **10** | 11 | 9 | 10 | 10 | 13 | 12 | 11 | 8 | 10 | 11 |
| Other acute or subacute ischaemic heart disease | **41** | **1** | 0 | 1 | 1 | 1 | 1 | 1 | 0 | 2 | 2 | 1 | **156** | **1** | 1 | 0 | 1 | 1 | 2 | 2 | 1 | 1 | 1 | 1 |
| Chronic ischaemic heart disease | **738** | **19** | 17 | 14 | 16 | 18 | 20 | 22 | 18 | 19 | 25 | 24 | **2,682** | **19** | 19 | 18 | 21 | 18 | 19 | 23 | 19 | 15 | 22 | 18 |
| Coronary reperfusion surgery and procedures | **457** | **12** | 12 | 8 | 8 | 13 | 12 | 14 | 9 | 12 | 16 | 15 | **1,676** | **12** | 11 | 11 | 13 | 10 | 12 | 15 | 12 | 11 | 14 | 13 |
| Conduction disorders | **124** | **3** | 3 | 2 | 2 | 5 | 3 | 2 | 3 | 4 | 4 | 4 | **416** | **3** | 2 | 2 | 2 | 4 | 2 | 4 | 3 | 4 | 5 | 4 |
| Cardiac arrest | **30** | **1** | 1 | 0 | 1 | 1 | 0 | 2 | 0 | 1 | 1 | 1 | **93** | **1** | 0 | 1 | 1 | 1 | 0 | 0 | 1 | 1 | 1 | 2 |
| Arrhythmias | **910** | **24** | 19 | 20 | 19 | 22 | 25 | 25 | 24 | 27 | 29 | 28 | **3,069** | **22** | 18 | 16 | 21 | 24 | 22 | 29 | 21 | 27 | 23 | 33 |
| Paroxysmal tachycardia | **174** | **5** | 3 | 4 | 4 | 5 | 5 | 4 | 5 | 4 | 4 | 6 | **609** | **4** | 4 | 4 | 3 | 3 | 5 | 7 | 4 | 6 | 5 | 7 |
| Ventricular tachycardia | **47** | **1** | 0 | 1 | 1 | 1 | 1 | 1 | 3 | 1 | 1 | 3 | **126** | **1** | 0 | 0 | 1 | 1 | 1 | 2 | 2 | 2 | 1 | 3 |
| Supraventricular tachycardia and unspecified | **112** | **3** | 3 | 3 | 2 | 4 | 4 | 3 | 2 | 3 | 2 | 4 | **415** | **3** | 4 | 3 | 3 | 1 | 4 | 4 | 2 | 3 | 3 | 4 |
| Atrial fibrillation and flutter | **760** | **20** | 15 | 16 | 15 | 18 | 19 | 22 | 21 | 23 | 26 | 23 | **2,546** | **18** | 14 | 13 | 18 | 20 | 20 | 21 | 17 | 24 | 20 | 30 |
| Other cardiac arrhythmias | **232** | **6** | 4 | 5 | 6 | 5 | 7 | 5 | 7 | 8 | 7 | 8 | **790** | **6** | 5 | 3 | 4 | 6 | 6 | 9 | 5 | 8 | 5 | 9 |
| Ventricular fibrillation and flutter | **13** | **0** | 0 | 0 | 1 | 1 | 0 | 0 | 1 | 0 | 0 | 0 | **50** | **0** | 0 | 0 | 0 | 0 | 0 | 0 | 1 | 0 | 0 | 0 |
| Other cardiac arrhythmias (subgroup) | **219** | **6** | 3 | 5 | 5 | 4 | 6 | 5 | 6 | 7 | 7 | 7 | **748** | **5** | 5 | 3 | 4 | 5 | 6 | 9 | 4 | 8 | 5 | 9 |
| Heart failure | **601** | **16** | 12 | 10 | 14 | 16 | 14 | 17 | 17 | 17 | 21 | 19 | **2,057** | **15** | 11 | 12 | 13 | 15 | 17 | 19 | 15 | 14 | 19 | 20 |
| Cerebrovascular disease | **682** | **18** | 11 | 15 | 11 | 16 | 19 | 16 | 17 | 20 | 23 | 31 | **2,301** | **16** | 13 | 14 | 16 | 17 | 18 | 18 | 18 | 19 | 19 | 27 |
| Cerebral haemorrhage (subarachnoid, intracerebral, other non-traumatic) | **64** | **2** | 1 | 1 | 1 | 1 | 2 | 1 | 1 | 2 | 3 | 5 | **169** | **1** | 1 | 1 | 1 | 1 | 1 | 0 | 1 | 1 | 2 | 5 |
| Cerebral infarction and stroke | **428** | **11** | 3 | 7 | 6 | 8 | 11 | 11 | 11 | 14 | 17 | 24 | **1,399** | **10** | 4 | 8 | 9 | 10 | 13 | 13 | 9 | 13 | 15 | 21 |
| Transient ischaemic attack | **233** | **6** | 5 | 4 | 5 | 6 | 7 | 6 | 6 | 6 | 6 | 10 | **750** | **5** | 4 | 4 | 5 | 6 | 6 | 6 | 7 | 6 | 5 | 7 |
| Other cerebrovascular disease and sequelae of cerebrovascular disease | **349** | **9** | 5 | 8 | 4 | 7 | 11 | 8 | 8 | 10 | 13 | 17 | **1,151** | **8** | 6 | 7 | 7 | 8 | 7 | 9 | 10 | 8 | 12 | 17 |
| Hypertension and hypertensive heart disease | **1,410** | **37** | 35 | 32 | 36 | 32 | 39 | 38 | 37 | 39 | 39 | 40 | **5,147** | **37** | 38 | 36 | 37 | 37 | 40 | 36 | 35 | 39 | 34 | 33 |
| Diseases of arteries, arterioles, and capillaries | **738** | **19** | 22 | 17 | 22 | 20 | 23 | 18 | 14 | 17 | 19 | 21 | **2,757** | **20** | 21 | 17 | 20 | 21 | 24 | 21 | 17 | 17 | 17 | 21 |
| Peripheral arterial revascularisation procedures | **313** | **8** | 5 | 9 | 8 | 10 | 9 | 7 | 5 | 9 | 9 | 11 | **1,094** | **8** | 9 | 6 | 6 | 8 | 10 | 10 | 6 | 7 | 8 | 8 |
| Other form of heart diseases | **1,106** | **29** | 17 | 23 | 28 | 27 | 32 | 28 | 31 | 27 | 39 | 36 | **3,753** | **27** | 20 | 22 | 26 | 30 | 30 | 30 | 28 | 25 | 37 | 36 |
| Hyperlipidaemia | **768** | **20** | 15 | 15 | 17 | 19 | 19 | 22 | 19 | 20 | 26 | 28 | **2,740** | **20** | 15 | 17 | 21 | 21 | 19 | 23 | 19 | 22 | 22 | 24 |
| Diabetes mellitus | **734** | **19** | 11 | 15 | 21 | 16 | 19 | 18 | 22 | 22 | 24 | 24 | **2,525** | **18** | 15 | 15 | 21 | 19 | 15 | 20 | 17 | 21 | 21 | 24 |
| Renal disease | **1,071** | **28** | 21 | 24 | 26 | 30 | 26 | 31 | 27 | 27 | 32 | 34 | **3,723** | **27** | 18 | 23 | 25 | 28 | 28 | 34 | 26 | 32 | 33 | 37 |
| Chronic kidney disease | **190** | **5** | 4 | 3 | 3 | 4 | 7 | 4 | 6 | 5 | 5 | 8 | **653** | **5** | 2 | 4 | 4 | 4 | 5 | 9 | 6 | 4 | 6 | 7 |
| Other renal disorders | **931** | **24** | 19 | 22 | 24 | 26 | 21 | 28 | 21 | 23 | 29 | 29 | **3,209** | **23** | 15 | 20 | 22 | 24 | 24 | 29 | 23 | 28 | 28 | 33 |
| Anaemias | **402** | **10** | 4 | 6 | 9 | 13 | 11 | 10 | 10 | 12 | 13 | 17 | **1,313** | **9** | 5 | 7 | 9 | 8 | 10 | 10 | 12 | 11 | 16 | 21 |
| Nutritional anaemias | **158** | **4** | 1 | 1 | 2 | 5 | 4 | 4 | 4 | 5 | 7 | 9 | **504** | **4** | 1 | 2 | 3 | 3 | 4 | 4 | 4 | 5 | 7 | 12 |
| Iron deficiency anaemias | **135** | **4** | 1 | 0 | 1 | 4 | 3 | 3 | 3 | 5 | 7 | 8 | **436** | **3** | 1 | 2 | 3 | 2 | 3 | 4 | 4 | 5 | 5 | 11 |
| Other anaemias | **336** | **9** | 4 | 5 | 8 | 11 | 10 | 8 | 9 | 9 | 10 | 13 | **1,113** | **8** | 4 | 6 | 8 | 8 | 8 | 9 | 9 | 8 | 14 | 15 |
| Peptic ulcer disease | **347** | **9** | 6 | 9 | 8 | 9 | 8 | 8 | 9 | 9 | 10 | 14 | **1,247** | **9** | 9 | 7 | 10 | 7 | 5 | 12 | 11 | 12 | 9 | 13 |
| Liver disease | **161** | **4** | 3 | 4 | 5 | 4 | 3 | 4 | 5 | 4 | 4 | 5 | **578** | **4** | 4 | 4 | 6 | 4 | 4 | 4 | 4 | 4 | 4 | 5 |
| Osteoporosis | **739** | **19** | 11 | 10 | 20 | 17 | 18 | 18 | 18 | 23 | 25 | 33 | **2,480** | **18** | 11 | 12 | 15 | 18 | 19 | 20 | 23 | 22 | 30 | 30 |
| Rheumatoid arthritis and other inflammatory arthropathies | **394** | **10** | 8 | 13 | 11 | 10 | 10 | 8 | 11 | 9 | 10 | 12 | **1,389** | **10** | 10 | 8 | 10 | 10 | 11 | 9 | 8 | 11 | 15 | 14 |
| Systemic connective tissue diseases | **169** | **4** | 6 | 4 | 4 | 4 | 3 | 5 | 4 | 5 | 4 | 4 | **646** | **5** | 6 | 5 | 3 | 5 | 4 | 4 | 6 | 5 | 3 | 5 |
| Malignancy | **968** | **25** | 21 | 21 | 24 | 23 | 26 | 24 | 27 | 25 | 32 | 30 | **3,307** | **24** | 17 | 23 | 23 | 25 | 24 | 24 | 24 | 25 | 29 | 34 |
| Depressive disorders | **218** | **6** | 6 | 6 | 5 | 4 | 4 | 6 | 6 | 8 | 5 | 7 | **789** | **6** | 6 | 5 | 7 | 5 | 4 | 7 | 7 | 5 | 5 | 7 |
| Pregnancy (at the index date) | **0** | **0** | 0 | 0 | 0 | 0 | 0 | 0 | 0 | 0 | 0 | 0 | **0** | **0** | 0 | 0 | 0 | 0 | 0 | 0 | 0 | 0 | 0 | 0 |
| Asthma | **495** | **13** | 8 | 10 | 12 | 17 | 13 | 11 | 13 | 14 | 16 | 16 | **1,790** | **13** | 9 | 11 | 14 | 13 | 15 | 14 | 11 | 16 | 17 | 16 |
| Respiratory medications | **2,960** | **77** | 45 | 56 | 59 | 69 | 78 | 89 | 91 | 93 | 94 | 95 | **9,711** | **69** | 44 | 51 | 59 | 69 | 79 | 84 | 91 | 93 | 95 | 96 |
| Inhaled short-acting muscarinic antagonists (SAMAs) | **13** | **0** | 0 | 0 | 0 | 1 | 0 | 0 | 1 | 0 | 0 | 1 | **39** | **0** | 0 | 0 | 0 | 0 | 0 | 0 | 0 | 1 | 1 | 1 |
| Inhaled long-acting muscarinic antagonists (LAMAs) | **1,436** | **37** | 1 | 3 | 5 | 16 | 29 | 51 | 62 | 65 | 70 | 72 | **3,770** | **27** | 1 | 2 | 6 | 14 | 30 | 49 | 62 | 67 | 73 | 72 |
| Inhaled short-acting beta2-agonists (SABAs) | **1,712** | **45** | 35 | 40 | 36 | 41 | 44 | 43 | 51 | 50 | 50 | 56 | **5,839** | **42** | 32 | 35 | 39 | 45 | 44 | 41 | 48 | 52 | 54 | 53 |
| Inhaled long-acting beta2-agonists (LABAs) | **0** | **0** | 0 | 0 | 0 | 0 | 0 | 0 | 0 | 0 | 0 | 0 | **0** | **0** | 0 | 0 | 0 | 0 | 0 | 0 | 0 | 0 | 0 | 0 |
| Inhaled glucocorticosteroids (ICS) | **514** | **13** | 3 | 10 | 11 | 17 | 11 | 12 | 14 | 15 | 20 | 21 | **1,624** | **12** | 4 | 6 | 12 | 14 | 18 | 11 | 12 | 20 | 16 | 21 |
| Fixed combinations of SABA and SAMA | **88** | **2** | 0 | 0 | 0 | 0 | 2 | 3 | 3 | 4 | 4 | 8 | **235** | **2** | 0 | 0 | 0 | 1 | 2 | 2 | 4 | 4 | 7 | 6 |
| Fixed combinations of SABA and ICS | **0** | **0** | 0 | 0 | 0 | 0 | 0 | 0 | 0 | 0 | 0 | 0 | **0** | **0** | 0 | 0 | 0 | 0 | 0 | 0 | 0 | 0 | 0 | 0 |
| Fixed combinations of LABA and ICS | **0** | **0** | 0 | 0 | 0 | 0 | 0 | 0 | 0 | 0 | 0 | 0 | **0** | **0** | 0 | 0 | 0 | 0 | 0 | 0 | 0 | 0 | 0 | 0 |
| Systemic glucocorticosteroids | **763** | **20** | 10 | 14 | 15 | 14 | 23 | 26 | 16 | 23 | 27 | 30 | **2,490** | **18** | 11 | 12 | 17 | 17 | 19 | 18 | 21 | 25 | 28 | 33 |
| Systemic beta2-agonists | **10** | **0** | 0 | 0 | 0 | 1 | 0 | 0 | 0 | 0 | 0 | 1 | **30** | **0** | 0 | 0 | 0 | 0 | 0 | 0 | 0 | 0 | 1 | 0 |
| Xanthines and adrenergics | **11** | **0** | 0 | 0 | 1 | 0 | 1 | 0 | 0 | 0 | 1 | 0 | **50** | **0** | 0 | 0 | 1 | 0 | 1 | 0 | 0 | 0 | 0 | 0 |
| Roflumilast | **0** | **0** | 0 | 0 | 0 | 0 | 0 | 0 | 0 | 0 | 0 | 0 | **0** | **0** | 0 | 0 | 0 | 0 | 0 | 0 | 0 | 0 | 0 | 0 |
| Nasal glucocorticosteroids | **182** | **5** | 4 | 5 | 5 | 5 | 4 | 5 | 6 | 5 | 3 | 3 | **668** | **5** | 4 | 4 | 5 | 4 | 7 | 5 | 5 | 5 | 5 | 4 |
| Omalizumab | **0** | **0** | 0 | 0 | 0 | 0 | 0 | 0 | 0 | 0 | 0 | 0 | **0** | **0** | 0 | 0 | 0 | 0 | 0 | 0 | 0 | 0 | 0 | 0 |
| Leukotriene receptor antagonists | **32** | **1** | 0 | 1 | 1 | 1 | 1 | 0 | 1 | 1 | 1 | 1 | **85** | **1** | 0 | 0 | 0 | 1 | 1 | 0 | 0 | 1 | 1 | 3 |
| Cromoglicic acid | **0** | **0** | 0 | 0 | 0 | 0 | 0 | 0 | 0 | 0 | 0 | 0 | **0** | **0** | 0 | 0 | 0 | 0 | 0 | 0 | 0 | 0 | 0 | 0 |
| Nedocromil | **0** | **0** | 0 | 0 | 0 | 0 | 0 | 0 | 0 | 0 | 0 | 0 | **0** | **0** | 0 | 0 | 0 | 0 | 0 | 0 | 0 | 0 | 0 | 0 |
| Oxygen therapy | **443** | **12** | 3 | 5 | 8 | 8 | 13 | 12 | 12 | 15 | 18 | 21 | **1,361** | **10** | 3 | 4 | 8 | 13 | 12 | 12 | 11 | 15 | 15 | 23 |
| Nebuliser therapy | **0** | **0** | 0 | 0 | 0 | 0 | 0 | 0 | 0 | 0 | 0 | 0 | **0** | **0** | 0 | 0 | 0 | 0 | 0 | 0 | 0 | 0 | 0 | 0 |
| Cardiovascular medications | **2,774** | **72** | 71 | 69 | 69 | 69 | 72 | 72 | 71 | 73 | 76 | 79 | **10,092** | **72** | 75 | 67 | 70 | 74 | 73 | 71 | 70 | 75 | 73 | 75 |
| Cardiac glycosides and antiarrhythmics, Class I and III | **239** | **6** | 3 | 3 | 4 | 5 | 7 | 8 | 6 | 8 | 11 | 6 | **717** | **5** | 2 | 3 | 5 | 4 | 7 | 6 | 8 | 6 | 8 | 10 |
| Vasodilators used in cardiac diseases | **278** | **7** | 6 | 5 | 7 | 6 | 6 | 9 | 6 | 10 | 8 | 9 | **969** | **7** | 7 | 6 | 7 | 7 | 7 | 8 | 6 | 7 | 9 | 9 |
| Cardiac stimulants and other cardiac preparations | **13** | **0** | 0 | 0 | 0 | 0 | 0 | 0 | 0 | 1 | 1 | 1 | **35** | **0** | 0 | 0 | 0 | 0 | 0 | 0 | 0 | 0 | 1 | 2 |
| Diuretics | **1,377** | **36** | 22 | 30 | 29 | 33 | 36 | 35 | 38 | 45 | 46 | 45 | **4,769** | **34** | 28 | 30 | 32 | 36 | 39 | 36 | 37 | 38 | 37 | 42 |
| Peripheral vasodilators | **0** | **0** | 0 | 0 | 0 | 0 | 0 | 0 | 0 | 0 | 0 | 0 | **0** | **0** | 0 | 0 | 0 | 0 | 0 | 0 | 0 | 0 | 0 | 0 |
| Vasoprotective agents | **84** | **2** | 4 | 2 | 2 | 1 | 2 | 3 | 1 | 1 | 3 | 3 | **298** | **2** | 2 | 3 | 2 | 2 | 2 | 3 | 2 | 1 | 2 | 5 |
| Beta blocking agents | **1,153** | **30** | 31 | 27 | 27 | 28 | 29 | 32 | 30 | 30 | 34 | 32 | **4,085** | **29** | 30 | 25 | 30 | 28 | 33 | 29 | 30 | 32 | 32 | 27 |
| Calcium channel blockers | **840** | **22** | 18 | 19 | 18 | 19 | 24 | 22 | 23 | 27 | 20 | 28 | **3,057** | **22** | 21 | 20 | 20 | 24 | 23 | 24 | 22 | 23 | 24 | 19 |
| Antihypertensives | **31** | **1** | 0 | 0 | 1 | 1 | 0 | 0 | 1 | 2 | 1 | 2 | **99** | **1** | 0 | 0 | 0 | 0 | 2 | 1 | 2 | 2 | 2 | 1 |
| Agents acting on the renin-angiotensin system | **1,436** | **37** | 42 | 39 | 39 | 35 | 37 | 36 | 34 | 37 | 40 | 36 | **5,400** | **38** | 44 | 37 | 38 | 39 | 40 | 37 | 35 | 38 | 34 | 32 |
| Angiotensin-converting-enzyme inhibitors | **712** | **19** | 24 | 21 | 20 | 18 | 18 | 18 | 17 | 17 | 17 | 16 | **2,772** | **20** | 27 | 19 | 20 | 22 | 19 | 18 | 18 | 19 | 13 | 11 |
| Angiotensin II receptor antagonists | **742** | **19** | 18 | 19 | 20 | 18 | 19 | 18 | 17 | 21 | 23 | 20 | **2,702** | **19** | 19 | 19 | 18 | 19 | 22 | 19 | 18 | 19 | 21 | 20 |
| Renin-inhibitors | **<5** | **0** | 0 | 0 | 0 | 0 | 0 | 0 | 0 | 0 | 0 | 0 | **<5** | **0** | 0 | 0 | 0 | 0 | 0 | 0 | 0 | 0 | 0 | 0 |
| Lipid-modifying agents | **1,463** | **38** | 47 | 38 | 40 | 38 | 40 | 38 | 36 | 31 | 38 | 36 | **5,581** | **40** | 51 | 38 | 36 | 39 | 42 | 37 | 38 | 37 | 33 | 30 |
| HMG-CoA reductase inhibitors (statins) | **1,438** | **37** | 46 | 37 | 40 | 37 | 39 | 37 | 35 | 30 | 38 | 36 | **5,472** | **39** | 50 | 37 | 36 | 38 | 42 | 37 | 36 | 36 | 33 | 30 |
| Other lipid-modifying agents | **47** | **1** | 1 | 1 | 1 | 3 | 1 | 1 | 1 | 1 | 1 | 1 | **193** | **1** | 1 | 1 | 2 | 1 | 1 | 1 | 2 | 2 | 1 | 1 |
| HMG-CoA reductase inhibitors (statins), other combinations with acetylsalicylic acid | **0** | **0** | 0 | 0 | 0 | 0 | 0 | 0 | 0 | 0 | 0 | 0 | **0** | **0** | 0 | 0 | 0 | 0 | 0 | 0 | 0 | 0 | 0 | 0 |
| Antithrombotic agents | **1,821** | **47** | 41 | 44 | 43 | 48 | 50 | 45 | 46 | 49 | 54 | 55 | **6,487** | **46** | 44 | 39 | 44 | 47 | 48 | 51 | 49 | 50 | 50 | 55 |
| Platelet aggregation inhibitors | **1,198** | **31** | 29 | 29 | 32 | 32 | 33 | 27 | 30 | 31 | 33 | 36 | **4,332** | **31** | 31 | 28 | 29 | 30 | 31 | 33 | 33 | 31 | 32 | 35 |
| Systemic antibacterials | **1,740** | **45** | 27 | 39 | 39 | 45 | 44 | 49 | 45 | 50 | 54 | 60 | **5,831** | **42** | 24 | 32 | 41 | 49 | 47 | 41 | 48 | 55 | 55 | 63 |
| Iron preparations | **63** | **2** | 0 | 1 | 1 | 1 | 1 | 2 | 2 | 2 | 3 | 3 | **191** | **1** | 1 | 1 | 1 | 1 | 2 | 1 | 2 | 1 | 3 | 4 |
| Proton pump inhibitors | **1,280** | **33** | 25 | 26 | 31 | 32 | 33 | 32 | 34 | 36 | 40 | 43 | **4,461** | **32** | 23 | 26 | 32 | 33 | 32 | 35 | 35 | 40 | 40 | 45 |
| Drugs used in diabetes | **556** | **14** | 9 | 12 | 16 | 11 | 15 | 14 | 15 | 17 | 18 | 18 | **1,926** | **14** | 12 | 12 | 15 | 16 | 11 | 15 | 12 | 17 | 15 | 18 |
| Insulins | **175** | **5** | 3 | 4 | 5 | 3 | 5 | 4 | 5 | 5 | 5 | 6 | **566** | **4** | 2 | 3 | 5 | 3 | 5 | 6 | 3 | 6 | 5 | 8 |
| Blood glucose–lowering drugs | **488** | **13** | 8 | 11 | 15 | 10 | 14 | 12 | 13 | 14 | 16 | 16 | **1,708** | **12** | 11 | 11 | 14 | 15 | 9 | 12 | 11 | 15 | 12 | 16 |
| Drugs for musculoskeletal system | **512** | **13** | 17 | 15 | 13 | 15 | 9 | 10 | 17 | 13 | 10 | 15 | **1,904** | **14** | 15 | 11 | 15 | 15 | 14 | 13 | 15 | 13 | 12 | 11 |
| Anti-inflammatory and antirheumatic products, non-steroids (non-steroidal anti-inflammatory drugs) | **494** | **13** | 17 | 15 | 13 | 15 | 9 | 10 | 16 | 13 | 9 | 12 | **1,847** | **13** | 15 | 11 | 15 | 14 | 14 | 13 | 14 | 12 | 11 | 9 |
| Acetylsalicylic acid (other analgesics and antipyretics) | **20** | **1** | 0 | 0 | 0 | 0 | 0 | 0 | 1 | 0 | 1 | 3 | **69** | **0** | 0 | 0 | 0 | 1 | 1 | 0 | 1 | 1 | 1 | 2 |
| Other antirheumatic agents: anti-inflammatory/antirheumatic agents in combination, specific antirheumatic agents | **0** | **0** | 0 | 0 | 0 | 0 | 0 | 0 | 0 | 0 | 0 | 0 | **0** | **0** | 0 | 0 | 0 | 0 | 0 | 0 | 0 | 0 | 0 | 0 |
| Antidepressants | **819** | **21** | 14 | 17 | 21 | 23 | 19 | 18 | 22 | 21 | 25 | 33 | **2,782** | **20** | 13 | 14 | 21 | 24 | 19 | 19 | 22 | 26 | 26 | 31 |
| Selective serotonin reuptake inhibitors | **459** | **12** | 6 | 8 | 11 | 12 | 9 | 12 | 11 | 14 | 16 | 19 | **1,560** | **11** | 6 | 8 | 13 | 14 | 10 | 12 | 13 | 14 | 15 | 16 |
| Antineoplastic agents | **<5** | **0** | 0 | 0 | 0 | 0 | 0 | 0 | 0 | 0 | 0 | 0 | **8** | **0** | 0 | 0 | 0 | 0 | 0 | 0 | 0 | 0 | 0 | 0 |
| Immunosuppressants | **65** | **2** | 1 | 1 | 2 | 2 | 1 | 2 | 3 | 2 | 1 | 2 | **193** | **1** | 1 | 1 | 1 | 2 | 1 | 1 | 2 | 2 | 2 | 3 |
| Antivirals for systemic use | **37** | **1** | 2 | 2 | 1 | 1 | 1 | 2 | 0 | 0 | 0 | 0 | **141** | **1** | 1 | 1 | 0 | 1 | 2 | 1 | 1 | 1 | 1 | 1 |
| Hormone-replacement therapy: Estrogens, progestogens, progestogens and estrogens in combination | **225** | **6** | 8 | 6 | 6 | 7 | 6 | 8 | 5 | 3 | 5 | 4 | **902** | **6** | 10 | 5 | 6 | 6 | 8 | 5 | 5 | 8 | 5 | 2 |
| Drugs used in nicotine dependence | **78** | **2** | 3 | 3 | 2 | 2 | 2 | 2 | 1 | 2 | 2 | 2 | **341** | **2** | 3 | 2 | 3 | 2 | 2 | 3 | 2 | 3 | 2 | 1 |
| **COPD severity category** |  |  |  |  |  |  |  |  |  |  |  |  |  |  |  |  |  |  |  |  |  |  |  |  |
| Mild | **792** | **21** | 63 | 49 | 33 | 20 | 15 | 6 | 6 | 6 | 4 | 3 | **3,991** | **28** | 63 | 51 | 34 | 20 | 12 | 12 | 8 | 4 | 5 | 3 |
| Moderate | **1,052** | **27** | 17 | 18 | 19 | 25 | 29 | 39 | 41 | 38 | 27 | 22 | **3,647** | **26** | 18 | 22 | 22 | 23 | 33 | 39 | 41 | 30 | 26 | 21 |
| Severe | **1,183** | **31** | 17 | 24 | 32 | 33 | 30 | 32 | 32 | 33 | 38 | 36 | **3,919** | **28** | 14 | 21 | 30 | 33 | 31 | 27 | 33 | 42 | 41 | 34 |
| Very severe | **816** | **21** | 3 | 9 | 16 | 21 | 26 | 23 | 21 | 23 | 30 | 39 | **2,472** | **18** | 5 | 7 | 15 | 25 | 25 | 22 | 19 | 24 | 28 | 43 |
| **CCI score** |  |  |  |  |  |  |  |  |  |  |  |  |  |  |  |  |  |  |  |  |  |  |  |  |
| 1 | **1,220** | **32** | 34 | 35 | 33 | 34 | 30 | 34 | 32 | 34 | 26 | 23 | **4,613** | **33** | 35 | 38 | 33 | 33 | 31 | 30 | 33 | 35 | 27 | 21 |
| 2 | **726** | **19** | 29 | 23 | 19 | 18 | 22 | 21 | 16 | 15 | 12 | 15 | **2,921** | **21** | 31 | 20 | 22 | 19 | 19 | 19 | 17 | 14 | 17 | 14 |
| 3+ | **1,897** | **49** | 37 | 42 | 48 | 48 | 49 | 45 | 51 | 51 | 61 | 62 | **6,495** | **46** | 34 | 41 | 45 | 49 | 50 | 51 | 50 | 51 | 56 | 65 |
| Hip fracture | **29** | **1** | 0 | 0 | 1 | 0 | 1 | 1 | 1 | 1 | 1 | 1 | **97** | **1** | 1 | 0 | 1 | 2 | 1 | 0 | 1 | 1 | 1 | 1 |
| Lung cancer | **104** | **3** | 0 | 0 | 1 | 0 | 4 | 3 | 3 | 2 | 6 | 8 | **212** | **2** | 0 | 1 | 0 | 1 | 1 | 3 | 2 | 3 | 5 | 5 |
| Other markers of bad fall | **17** | **0** | 1 | 0 | 0 | 1 | 0 | 1 | 1 | 1 | 0 | 0 | **67** | **0** | 1 | 0 | 1 | 1 | 0 | 0 | 0 | 1 | 0 | 0 |
| Metastatic cancer | **30** | **1** | 1 | 0 | 1 | 1 | 2 | 0 | 1 | 0 | 2 | 1 | **62** | **0** | 0 | 0 | 1 | 0 | 0 | 1 | 0 | 0 | 1 | 1 |
| Pulmonary cachexia | **15** | **0** | 0 | 1 | 1 | 0 | 1 | 0 | 1 | 0 | 0 | 0 | **41** | **0** | 0 | 0 | 0 | 0 | 1 | 0 | 1 | 0 | 1 | 1 |
| Right-sided heart failure | **26** | **1** | 0 | 0 | 1 | 1 | 1 | 1 | 0 | 2 | 1 | 1 | **68** | **0** | 1 | 0 | 0 | 1 | 0 | 0 | 0 | 1 | 1 | 1 |
| **Number of hospitalisations within 180 days** |  |  |  |  |  |  |  |  |  |  |  |  |  |  |  |  |  |  |  |  |  |  |  |  |
| 0 | **2,871** | **75** | 93 | 85 | 77 | 74 | 73 | 77 | 76 | 70 | 64 | 56 | **10,980** | **78** | 91 | 87 | 81 | 74 | 75 | 72 | 73 | 73 | 64 | 57 |
| 1 | **680** | **18** | 5 | 13 | 18 | 18 | 21 | 17 | 18 | 19 | 22 | 25 | **2,282** | **16** | 8 | 11 | 16 | 22 | 18 | 19 | 18 | 17 | 24 | 28 |
| 2 | **187** | **5** | 1 | 2 | 3 | 5 | 4 | 4 | 2 | 7 | 10 | 10 | **484** | **3** | 0 | 1 | 2 | 3 | 4 | 6 | 6 | 7 | 7 | 8 |
| 3-4 | **83** | **2** | 1 | 0 | 1 | 2 | 1 | 1 | 3 | 3 | 3 | 7 | **211** | **2** | 0 | 0 | 1 | 1 | 1 | 2 | 4 | 2 | 5 | 6 |
| 5+ | **22** | **1** | 0 | 0 | 1 | 1 | 0 | 1 | 1 | 1 | 0 | 1 | **72** | **1** | 0 | 1 | 0 | 0 | 1 | 1 | 0 | 1 | 0 | 1 |
| **Number of hospitalisations within 365 days** |  |  |  |  |  |  |  |  |  |  |  |  |  |  |  |  |  |  |  |  |  |  |  |  |
| 0 | **2,181** | **57** | 81 | 70 | 61 | 55 | 53 | 56 | 59 | 54 | 42 | 36 | **8,591** | **61** | 80 | 73 | 60 | 54 | 55 | 56 | 61 | 52 | 41 | 40 |
| 1 | **940** | **24** | 12 | 19 | 23 | 26 | 27 | 25 | 24 | 26 | 32 | 31 | **3,222** | **23** | 15 | 18 | 27 | 29 | 26 | 22 | 22 | 25 | 32 | 27 |
| 2 | **375** | **10** | 5 | 8 | 8 | 10 | 13 | 10 | 7 | 11 | 11 | 15 | **1,212** | **9** | 3 | 6 | 8 | 10 | 11 | 13 | 9 | 12 | 12 | 14 |
| 3-4 | **270** | **7** | 2 | 3 | 6 | 7 | 6 | 7 | 9 | 8 | 10 | 12 | **801** | **6** | 2 | 3 | 5 | 6 | 6 | 8 | 7 | 9 | 11 | 11 |
| 5+ | **77** | **2** | 0 | 0 | 1 | 1 | 1 | 2 | 1 | 2 | 5 | 7 | **203** | **1** | 0 | 1 | 0 | 1 | 2 | 2 | 2 | 2 | 4 | 8 |
| **Number of hospitalisations with COPD within 90 days** |  |  |  |  |  |  |  |  |  |  |  |  |  |  |  |  |  |  |  |  |  |  |  |  |
| 0 | **3,843** | **100** | 100 | 100 | 100 | 100 | 100 | 100 | 100 | 100 | 100 | 100 | **14,029** | **100** | 100 | 100 | 100 | 100 | 100 | 100 | 100 | 100 | 100 | 100 |
| **Number of hospitalisations with COPD within 180 days** |  |  |  |  |  |  |  |  |  |  |  |  |  |  |  |  |  |  |  |  |  |  |  |  |
| 0 | **3,559** | **93** | 98 | 97 | 93 | 93 | 96 | 92 | 93 | 91 | 89 | 86 | **13,148** | **94** | 98 | 97 | 95 | 93 | 92 | 93 | 91 | 90 | 89 | 85 |
| 1 | **248** | **6** | 2 | 3 | 7 | 6 | 4 | 7 | 6 | 8 | 9 | 13 | **757** | **5** | 2 | 2 | 4 | 7 | 6 | 5 | 7 | 9 | 10 | 13 |
| 2+ | **36** | **1** | 1 | 1 | 1 | 1 | 0 | 1 | 1 | 1 | 3 | 1 | **124** | **1** | 0 | 1 | 0 | 1 | 1 | 2 | 2 | 1 | 1 | 2 |
| **Number of COPD exacerbations within 90 days** |  |  |  |  |  |  |  |  |  |  |  |  |  |  |  |  |  |  |  |  |  |  |  |  |
| 0 | **2,690** | **70** | 81 | 75 | 74 | 75 | 68 | 64 | 71 | 68 | 63 | 61 | **10,089** | **72** | 82 | 78 | 73 | 71 | 70 | 69 | 65 | 62 | 64 | 61 |
| 1 | **911** | **24** | 17 | 22 | 21 | 21 | 25 | 28 | 24 | 26 | 26 | 27 | **3,203** | **23** | 17 | 20 | 23 | 25 | 25 | 26 | 25 | 24 | 28 | 27 |
| 2 | **196** | **5** | 1 | 2 | 5 | 3 | 5 | 6 | 4 | 6 | 9 | 10 | **627** | **4** | 1 | 2 | 4 | 3 | 5 | 5 | 8 | 11 | 6 | 9 |
| 3+ | **46** | **1** | 0 | 1 | 1 | 1 | 1 | 2 | 1 | 1 | 3 | 2 | **110** | **1** | 0 | 0 | 1 | 1 | 1 | 1 | 2 | 2 | 1 | 3 |
| **Number of COPD exacerbations within 180 days** |  |  |  |  |  |  |  |  |  |  |  |  |  |  |  |  |  |  |  |  |  |  |  |  |
| 0 | **2,119** | **55** | 72 | 64 | 60 | 59 | 55 | 49 | 56 | 49 | 47 | 41 | **8,230** | **59** | 76 | 67 | 58 | 54 | 54 | 59 | 51 | 47 | 46 | 36 |
| 1 | **1,033** | **27** | 22 | 25 | 24 | 25 | 26 | 28 | 30 | 33 | 25 | 31 | **3,632** | **26** | 19 | 24 | 28 | 30 | 27 | 23 | 28 | 27 | 30 | 36 |
| 2 | **447** | **12** | 6 | 10 | 13 | 11 | 12 | 14 | 10 | 10 | 17 | 13 | **1,489** | **11** | 5 | 8 | 12 | 13 | 11 | 11 | 15 | 15 | 15 | 9 |
| 3+ | **244** | **6** | 1 | 1 | 3 | 5 | 7 | 9 | 4 | 8 | 11 | 15 | **678** | **5** | 0 | 1 | 2 | 3 | 8 | 6 | 7 | 12 | 9 | 18 |

Data were handled according to the Danish Act on Processing of Personal Data. The possibility of unintentional (deductive) disclosure arises when cells with small numbers of patients are quoted. When reporting the data, Danish policy is that no cell should contain fewer than 5 events. Cells with 0 counts are reported. Cells with “<5” indicate counts between 1 and 4, and exact number and percentage are not reported. To avoid back calculation, when needed, complementary cells are not reported.

Table S15. Standardised Bias for the Comparison of Key Covariates Between New Users of Olodaterol and of Other LABAs in Propensity Score–Trimmed Study Cohorts by Propensity Score Deciles, Restricting the Population to Users of LABA/LAMA Who Are LABA Naive and Have No Hospitalisations for COPD in the Last 90 Days

| **Variables** | **Untrimmed (overall)** | **Trimmed (overall)** | **Trimmed (averaged)** | STdiff D1 | STdiff D2 | STdiff D3 | STdiff D4 | STdiff D5 | STdiff D6 | STdiff D7 | STdiff D8 | STdiff D9 | STdiff D10 |
| --- | --- | --- | --- | --- | --- | --- | --- | --- | --- | --- | --- | --- | --- |
| Age group (years): 40-60 | **-0.01249** | **-0.00039** | **0.01** | -0.01 | 0.03780 | -0.02920 | -0.00923 | 0.00030 | 0.00712 | -0.06455 | -0.03241 | 0.06251 | 0.10037 |
| Age group (years): 61-74 | **0.01083** | **0.01950** | **0.01** | 0.08 | -0.00875 | 0.09905 | -0.03347 | -0.01765 | 0.00250 | 0.05995 | -0.01054 | -0.01925 | -0.07118 |
| Age group (years): 75-84 | **0.00529** | **-0.01413** | **-0.01** | -0.01 | -0.01439 | -0.08389 | 0.04531 | -0.04597 | -0.05845 | -0.01248 | 0.03248 | -0.03305 | 0.04225 |
| Age group (years): 85 or more | **-0.01223** | **-0.01085** | **-0.01** | -0.11 | -0.01356 | -0.00201 | -0.00205 | 0.09658 | 0.07725 | -0.01141 | 0.00391 | 0.00291 | -0.09806 |
| Female | **-0.00098** | **-0.00464** | **0.01** | 0.05 | -0.09130 | -0.11731 | -0.05925 | 0.05374 | 0.08072 | 0.04194 | 0.02345 | 0.06619 | 0.04478 |
| Male | **0.00098** | **0.00464** | **-0.01** | -0.05 | 0.09130 | 0.11731 | 0.05925 | -0.05374 | -0.08072 | -0.04194 | -0.02345 | -0.06619 | -0.04478 |
| Calendar year at index: 2015 | **-0.00061** | **-0.00530** | **-0.00** | 0.07 | -0.03507 | 0.05957 | -0.02739 | -0.06985 | -0.06898 | -0.15581 | -0.01560 | 0.08282 | 0.12639 |
| Calendar year at index: 2016 | **0.00894** | **-0.00358** | **-0.01** | 0.02 | 0.07397 | -0.04111 | -0.05619 | 0.02071 | -0.08268 | -0.00160 | 0.04058 | 0.01555 | -0.04483 |
| Calendar year at index: 2017 | **0.00622** | **0.00731** | **0.00** | -0.07 | 0.03493 | 0.03469 | 0.01023 | -0.04232 | 0.05694 | -0.04126 | 0.12960 | -0.02298 | -0.06767 |
| Calendar year at index: 2018-2019 | **-0.01322** | **-0.00148** | **-0.00** | 0.01 | -0.08044 | -0.03162 | 0.04683 | 0.05605 | 0.04619 | 0.10945 | -0.16008 | -0.03679 | 0.03109 |
| Cardiovascular diseases: No | **0.03690** | **0.01795** | **-0.01** | -0.15 | 0.07718 | -0.01722 | -0.00362 | -0.04378 | -0.06762 | 0.00610 | -0.03870 | 0.05348 | 0.13256 |
| Cardiovascular diseases: Yes | **-0.03690** | **-0.01795** | **0.01** | 0.15 | -0.07718 | 0.01722 | 0.00362 | 0.04378 | 0.06762 | -0.00610 | 0.03870 | -0.05348 | -0.13256 |
| Ischaemic heart disease: No | **0.00788** | **-0.00251** | **0.00** | -0.04 | -0.06246 | -0.09072 | 0.05072 | -0.06428 | -0.10332 | 0.05163 | 0.00991 | 0.06029 | 0.19109 |
| Ischaemic heart disease: Yes | **-0.00788** | **0.00251** | **-0.00** | 0.04 | 0.06246 | 0.09072 | -0.05072 | 0.06428 | 0.10332 | -0.05163 | -0.00991 | -0.06029 | -0.19109 |
| Angina pectoris: No | **0.00567** | **0.00000** | **0.00** | -0.01 | -0.04482 | -0.01020 | 0.02985 | -0.14156 | -0.02483 | 0.00573 | -0.02449 | 0.06906 | 0.15807 |
| Angina pectoris: Yes | **-0.00567** | **-0.00000** | **-0.00** | 0.01 | 0.04482 | 0.01020 | -0.02985 | 0.14156 | 0.02483 | -0.00573 | 0.02449 | -0.06906 | -0.15807 |
| Acute myocardial infarction: No | **0.01223** | **-0.00514** | **-0.00** | -0.03 | -0.05050 | -0.03231 | -0.00947 | -0.06298 | -0.01753 | -0.03692 | 0.03924 | 0.10116 | 0.06297 |
| Acute myocardial infarction: Yes | **-0.01223** | **0.00514** | **0.00** | 0.03 | 0.05050 | 0.03231 | 0.00947 | 0.06298 | 0.01753 | 0.03692 | -0.03924 | -0.10116 | -0.06297 |
| Other acute or subacute ischaemic heart disease: No | **0.00501** | **-0.00430** | **0.01** | -0.08 | 0.10911 | -0.00300 | 0.09729 | -0.06811 | -0.08596 | -0.07726 | 0.12943 | 0.05781 | -0.00647 |
| Other acute or subacute ischaemic heart disease: Yes | **-0.00501** | **0.00430** | **-0.01** | 0.08 | -0.10911 | 0.00300 | -0.09729 | 0.06811 | 0.08596 | 0.07726 | -0.12943 | -0.05781 | 0.00647 |
| Chronic ischaemic heart disease: No | **0.01280** | **0.00219** | **0.00** | -0.07 | -0.10132 | -0.10916 | 0.00742 | 0.02177 | -0.02681 | -0.02965 | 0.10648 | 0.05609 | 0.17565 |
| Chronic ischaemic heart disease: Yes | **-0.01280** | **-0.00219** | **-0.00** | 0.07 | 0.10132 | 0.10916 | -0.00742 | -0.02177 | 0.02681 | 0.02965 | -0.10648 | -0.05609 | -0.17565 |
| Coronary reperfusion surgery and procedures: No | **0.01215** | **-0.00169** | **-0.01** | 0.01 | -0.10175 | -0.13695 | 0.08770 | 0.01535 | -0.02272 | -0.08616 | 0.04704 | 0.06239 | 0.05147 |
| Coronary reperfusion surgery and procedures: Yes | **-0.01215** | **0.00169** | **0.01** | -0.01 | 0.10175 | 0.13695 | -0.08770 | -0.01535 | 0.02272 | 0.08616 | -0.04704 | -0.06239 | -0.05147 |
| Conduction disorders: No | **0.01042** | **0.01541** | **0.00** | 0.07 | 0.00963 | -0.00690 | 0.02251 | 0.06905 | -0.10947 | 0.01215 | 0.02880 | -0.04646 | -0.00148 |
| Conduction disorders: Yes | **-0.01042** | **-0.01541** | **-0.00** | -0.07 | -0.00963 | 0.00690 | -0.02251 | -0.06905 | 0.10947 | -0.01215 | -0.02880 | 0.04646 | 0.00148 |
| Cardiac arrest: No | **0.01310** | **0.01451** | **0.03** | 0.07 | -0.04901 | -0.03616 | -0.00443 | -0.05256 | 0.40790 | -0.07120 | 0.10927 | 0.00450 | -0.04919 |
| Cardiac arrest: Yes | **-0.01310** | **-0.01451** | **-0.04** | -0.07 | 0.04901 | 0.03616 | 0.00443 | . | -0.40790 | 0.07120 | -0.10927 | -0.00450 | 0.04919 |
| Arrhythmias: No | **0.04976** | **0.04362** | **0.01** | 0.03 | 0.11682 | -0.05125 | -0.04871 | 0.05012 | -0.08325 | 0.09362 | 0.00167 | 0.13046 | -0.12512 |
| Arrhythmias: Yes | **-0.04976** | **-0.04362** | **-0.01** | -0.03 | -0.11682 | 0.05125 | 0.04871 | -0.05012 | 0.08325 | -0.09362 | -0.00167 | -0.13046 | 0.12512 |
| Paroxysmal tachycardia: No | **0.02057** | **0.00916** | **0.00** | -0.04 | 0.04114 | 0.03486 | 0.13687 | 0.03436 | -0.10839 | 0.04815 | -0.05905 | -0.04465 | -0.03088 |
| Paroxysmal tachycardia: Yes | **-0.02057** | **-0.00916** | **-0.00** | 0.04 | -0.04114 | -0.03486 | -0.13687 | -0.03436 | 0.10839 | -0.04815 | 0.05905 | 0.04465 | 0.03088 |
| Ventricular tachycardia: No | **0.03874** | **0.03443** | **0.02** | 0.02 | 0.01529 | 0.07663 | 0.01881 | 0.05409 | -0.05788 | 0.05942 | -0.08565 | 0.03151 | 0.01646 |
| Ventricular tachycardia: Yes | **-0.03874** | **-0.03443** | **-0.02** | -0.02 | -0.01529 | -0.07663 | -0.01881 | -0.05409 | 0.05788 | -0.05942 | 0.08565 | -0.03151 | -0.01646 |
| Supraventricular tachycardia and unspecified: No | **0.00127** | **-0.00258** | **0.01** | -0.06 | 0.04431 | -0.04376 | 0.24780 | -0.01046 | -0.04094 | 0.03664 | -0.01418 | -0.09380 | 0.02090 |
| Supraventricular tachycardia and unspecified: Yes | **-0.00127** | **0.00258** | **-0.01** | 0.06 | -0.04431 | 0.04376 | -0.24780 | 0.01046 | 0.04094 | -0.03664 | 0.01418 | 0.09380 | -0.02090 |
| Atrial fibrillation and flutter: No | **0.05352** | **0.04224** | **0.01** | 0.05 | 0.11292 | -0.08878 | -0.06527 | -0.01258 | 0.01858 | 0.09053 | -0.00231 | 0.13326 | -0.14864 |
| Atrial fibrillation and flutter: Yes | **-0.05352** | **-0.04224** | **-0.01** | -0.05 | -0.11292 | 0.08878 | 0.06527 | 0.01258 | -0.01858 | -0.09053 | 0.00231 | -0.13326 | 0.14864 |
| Other cardiac arrhythmias: No | **0.02251** | **0.01760** | **0.01** | -0.06 | 0.11267 | 0.06184 | -0.04301 | 0.02257 | -0.15033 | 0.10217 | -0.02695 | 0.08776 | -0.05643 |
| Other cardiac arrhythmias: Yes | **-0.02251** | **-0.01760** | **-0.01** | 0.06 | -0.11267 | -0.06184 | 0.04301 | -0.02257 | 0.15033 | -0.10217 | 0.02695 | -0.08776 | 0.05643 |
| Other cardiac arrhythmias (subgroup): No | **0.02140** | **0.01633** | **0.01** | -0.06 | 0.10490 | 0.06467 | -0.04820 | 0.02061 | -0.15033 | 0.15442 | -0.03641 | 0.09592 | -0.05993 |
| Other cardiac arrhythmias (subgroup): Yes | **-0.02140** | **-0.01633** | **-0.01** | 0.06 | -0.10490 | -0.06467 | 0.04820 | -0.02061 | 0.15033 | -0.15442 | 0.03641 | -0.09592 | 0.05993 |
| Heart failure: No | **0.04464** | **0.02760** | **0.00** | 0.04 | -0.08309 | 0.01452 | 0.00577 | -0.08971 | -0.04764 | 0.06389 | 0.10540 | 0.02887 | -0.00810 |
| Heart failure: Yes | **-0.04464** | **-0.02760** | **-0.00** | -0.04 | 0.08309 | -0.01452 | -0.00577 | 0.08971 | 0.04764 | -0.06389 | -0.10540 | -0.02887 | 0.00810 |
| Cerebrovascular disease: No | **0.06127** | **0.03632** | **0.00** | -0.06 | 0.03993 | -0.12987 | -0.02619 | 0.03937 | -0.05326 | -0.03256 | 0.03848 | 0.10161 | 0.09248 |
| Cerebrovascular disease: Yes | **-0.06127** | **-0.03632** | **-0.00** | 0.06 | -0.03993 | 0.12987 | 0.02619 | -0.03937 | 0.05326 | 0.03256 | -0.03848 | -0.10161 | -0.09248 |
| Cerebral haemorrhage (subarachnoid, intracerebral, other non-traumatic): No | **0.05111** | **0.04223** | **0.03** | -0.06 | 0.00365 | 0.00395 | 0.05159 | 0.03863 | 0.15959 | -0.07645 | 0.01864 | 0.14120 | -0.00471 |
| Cerebral haemorrhage (subarachnoid, intracerebral, other non-traumatic): Yes | **-0.05111** | **-0.04223** | **-0.03** | 0.06 | -0.00365 | -0.00395 | -0.05159 | -0.03863 | -0.15959 | 0.07645 | -0.01864 | -0.14120 | 0.00471 |
| Cerebral infarction and stroke: No | **0.08437** | **0.03888** | **-0.01** | -0.03 | -0.01943 | -0.11005 | -0.08457 | -0.05914 | -0.06318 | 0.04939 | 0.03423 | 0.07676 | 0.06689 |
| Cerebral infarction and stroke: Yes | **-0.08437** | **-0.03888** | **0.01** | 0.03 | 0.01943 | 0.11005 | 0.08457 | 0.05914 | 0.06318 | -0.04939 | -0.03423 | -0.07676 | -0.06689 |
| Transient ischaemic attack: No | **0.02471** | **0.03187** | **0.02** | 0.03 | 0.00579 | -0.03165 | 0.01312 | 0.03051 | 0.01663 | -0.03089 | 0.02837 | 0.02984 | 0.09434 |
| Transient ischaemic attack: Yes | **-0.02471** | **-0.03187** | **-0.02** | -0.03 | -0.00579 | 0.03165 | -0.01312 | -0.03051 | -0.01663 | 0.03089 | -0.02837 | -0.02984 | -0.09434 |
| Other cerebrovascular disease and sequelae of cerebrovascular disease: No | **0.05519** | **0.03196** | **-0.00** | -0.03 | 0.03310 | -0.10513 | -0.04600 | 0.13411 | -0.04766 | -0.06436 | 0.07706 | 0.03918 | -0.00574 |
| Other cerebrovascular disease and sequelae of cerebrovascular disease: Yes | **-0.05519** | **-0.03196** | **0.00** | 0.03 | -0.03310 | 0.10513 | 0.04600 | -0.13411 | 0.04766 | 0.06436 | -0.07706 | -0.03918 | 0.00574 |
| Hypertension and hypertensive heart disease: No | **0.00375** | **0.00004** | **0.01** | -0.06 | -0.08150 | -0.01006 | -0.09252 | -0.01874 | 0.04115 | 0.03847 | 0.01767 | 0.09523 | 0.15071 |
| Hypertension and hypertensive heart disease: Yes | **-0.00375** | **-0.00004** | **-0.01** | 0.06 | 0.08150 | 0.01006 | 0.09252 | 0.01874 | -0.04115 | -0.03847 | -0.01767 | -0.09523 | -0.15071 |
| Diseases of arteries, arterioles, and capillaries: No | **-0.00485** | **-0.01128** | **-0.01** | 0.02 | -0.00470 | 0.05369 | -0.02987 | -0.01360 | -0.07719 | -0.06766 | -0.00898 | 0.04440 | 0.00941 |
| Diseases of arteries, arterioles, and capillaries: Yes | **0.00485** | **0.01128** | **0.01** | -0.02 | 0.00470 | -0.05369 | 0.02987 | 0.01360 | 0.07719 | 0.06766 | 0.00898 | -0.04440 | -0.00941 |
| Peripheral arterial revascularisation procedures: No | **0.01280** | **0.01292** | **0.02** | -0.11 | 0.09997 | 0.07307 | 0.05663 | -0.02402 | -0.10160 | -0.02251 | 0.06038 | 0.02492 | 0.10026 |
| Peripheral arterial revascularisation procedures: Yes | **-0.01280** | **-0.01292** | **-0.02** | 0.11 | -0.09997 | -0.07307 | -0.05663 | 0.02402 | 0.10160 | 0.02251 | -0.06038 | -0.02492 | -0.10026 |
| Other form of heart diseases: No | **0.05926** | **0.04581** | **0.01** | -0.08 | 0.03991 | 0.04968 | -0.07196 | 0.03222 | -0.03278 | 0.06883 | 0.03646 | 0.02924 | 0.00058 |
| Other form of heart diseases: Yes | **-0.05926** | **-0.04581** | **-0.01** | 0.08 | -0.03991 | -0.04968 | 0.07196 | -0.03222 | 0.03278 | -0.06883 | -0.03646 | -0.02924 | -0.00058 |
| Hyperlipidaemia: No | **0.03688** | **0.01144** | **-0.01** | -0.02 | -0.06928 | -0.09625 | -0.05997 | -0.00254 | -0.01098 | 0.01093 | -0.05389 | 0.10331 | 0.11319 |
| Hyperlipidaemia: Yes | **-0.03688** | **-0.01144** | **0.01** | 0.02 | 0.06928 | 0.09625 | 0.05997 | 0.00254 | 0.01098 | -0.01093 | 0.05389 | -0.10331 | -0.11319 |
| Diabetes mellitus: No | **0.04492** | **0.02866** | **0.01** | -0.11 | -0.01074 | -0.00942 | -0.08554 | 0.10173 | -0.02687 | 0.13221 | 0.03339 | 0.06526 | -0.00424 |
| Diabetes mellitus: Yes | **-0.04492** | **-0.02866** | **-0.01** | 0.11 | 0.01074 | 0.00942 | 0.08554 | -0.10173 | 0.02687 | -0.13221 | -0.03339 | -0.06526 | 0.00424 |
| Renal disease: No | **0.06051** | **0.03014** | **-0.01** | 0.09 | 0.03217 | 0.02089 | 0.05072 | -0.05007 | -0.06508 | 0.00742 | -0.10077 | -0.02186 | -0.06982 |
| Renal disease: Yes | **-0.06051** | **-0.03014** | **0.01** | -0.09 | -0.03217 | -0.02089 | -0.05072 | 0.05007 | 0.06508 | -0.00742 | 0.10077 | 0.02186 | 0.06982 |
| Chronic kidney disease: No | **0.03333** | **0.01374** | **-0.00** | 0.13 | -0.03954 | -0.05095 | 0.01620 | 0.07334 | -0.16163 | 0.01862 | 0.05330 | -0.07124 | 0.02618 |
| Chronic kidney disease: Yes | **-0.03333** | **-0.01374** | **0.00** | -0.13 | 0.03954 | 0.05095 | -0.01620 | -0.07334 | 0.16163 | -0.01862 | -0.05330 | 0.07124 | -0.02618 |
| Other renal disorders: No | **0.05556** | **0.03218** | **-0.00** | 0.10 | 0.04862 | 0.05021 | 0.05501 | -0.06136 | -0.04239 | -0.03167 | -0.10088 | 0.02085 | -0.08581 |
| Other renal disorders: Yes | **-0.05556** | **-0.03218** | **0.00** | -0.10 | -0.04862 | -0.05021 | -0.05501 | 0.06136 | 0.04239 | 0.03167 | 0.10088 | -0.02085 | 0.08581 |
| Anaemias: No | **0.07143** | **0.03781** | **-0.01** | -0.03 | -0.04748 | 0.02131 | 0.16964 | 0.03657 | 0.00021 | -0.05165 | 0.03022 | -0.10378 | -0.10555 |
| Anaemias: Yes | **-0.07143** | **-0.03781** | **0.01** | 0.03 | 0.04748 | -0.02131 | -0.16964 | -0.03657 | -0.00021 | 0.05165 | -0.03022 | 0.10378 | 0.10555 |
| Nutritional anaemias: No | **0.07210** | **0.02788** | **-0.02** | -0.03 | -0.10634 | -0.05137 | 0.13170 | 0.01297 | -0.03921 | -0.03169 | -0.00811 | 0.01394 | -0.09080 |
| Nutritional anaemias: Yes | **-0.07210** | **-0.02788** | **0.02** | 0.03 | 0.10634 | 0.05137 | -0.13170 | -0.01297 | 0.03921 | 0.03169 | 0.00811 | -0.01394 | 0.09080 |
| Iron deficiency anaemias: No | **0.06909** | **0.02334** | **-0.02** | -0.00 | -0.11700 | -0.08250 | 0.13791 | 0.01703 | -0.07702 | -0.04033 | -0.01791 | 0.07730 | -0.09370 |
| Iron deficiency anaemias: Yes | **-0.06909** | **-0.02334** | **0.02** | 0.00 | 0.11700 | 0.08250 | -0.13791 | -0.01703 | 0.07702 | 0.04033 | 0.01791 | -0.07730 | 0.09370 |
| Other anaemias: No | **0.05333** | **0.02996** | **-0.00** | -0.04 | -0.03522 | -0.00082 | 0.13334 | 0.08960 | -0.02860 | -0.02874 | 0.04325 | -0.11978 | -0.05536 |
| Other anaemias: Yes | **-0.05333** | **-0.02996** | **0.00** | 0.04 | 0.03522 | 0.00082 | -0.13334 | -0.08960 | 0.02860 | 0.02874 | -0.04325 | 0.11978 | 0.05536 |
| Peptic ulcer disease: No | **0.02260** | **0.00494** | **-0.01** | -0.08 | 0.06935 | -0.04415 | 0.06102 | 0.12443 | -0.11536 | -0.06451 | -0.08100 | 0.03456 | 0.02956 |
| Peptic ulcer disease: Yes | **-0.02260** | **-0.00494** | **0.01** | 0.08 | -0.06935 | 0.04415 | -0.06102 | -0.12443 | 0.11536 | 0.06451 | 0.08100 | -0.03456 | -0.02956 |
| Liver disease: No | **-0.00237** | **0.00349** | **0.00** | -0.04 | -0.00297 | -0.00195 | -0.02505 | -0.01716 | 0.00526 | 0.10517 | -0.01210 | 0.01733 | 0.00952 |
| Liver disease: Yes | **0.00237** | **-0.00349** | **-0.00** | 0.04 | 0.00297 | 0.00195 | 0.02505 | 0.01716 | -0.00526 | -0.10517 | 0.01210 | -0.01733 | -0.00952 |
| Osteoporosis: No | **0.07951** | **0.04069** | **-0.02** | -0.01 | -0.07660 | 0.14756 | -0.02286 | -0.02358 | -0.04853 | -0.11038 | 0.03033 | -0.11268 | 0.05100 |
| Osteoporosis: Yes | **-0.07951** | **-0.04069** | **0.02** | 0.01 | 0.07660 | -0.14756 | 0.02286 | 0.02358 | 0.04853 | 0.11038 | -0.03033 | 0.11268 | -0.05100 |
| Rheumatoid arthritis and other inflammatory arthropathies: No | **0.01258** | **0.01177** | **0.00** | -0.05 | 0.16526 | 0.04624 | 0.00180 | -0.03498 | -0.02743 | 0.14426 | -0.03909 | -0.13635 | -0.04027 |
| Rheumatoid arthritis and other inflammatory arthropathies: Yes | **-0.01258** | **-0.01177** | **-0.00** | 0.05 | -0.16526 | -0.04624 | -0.00180 | 0.03498 | 0.02743 | -0.14426 | 0.03909 | 0.13635 | 0.04027 |
| Systemic connective tissue diseases: No | **-0.01921** | **-0.00988** | **-0.00** | 0.02 | -0.02892 | 0.09144 | -0.02308 | -0.07065 | 0.06020 | -0.07442 | 0.00603 | 0.05258 | -0.06409 |
| Systemic connective tissue diseases: Yes | **0.01921** | **0.00988** | **0.00** | -0.02 | 0.02892 | -0.09144 | 0.02308 | 0.07065 | -0.06020 | 0.07442 | -0.00603 | -0.05258 | 0.06409 |
| Malignancy: No | **0.04910** | **0.03807** | **0.01** | 0.09 | -0.04687 | 0.01930 | -0.04381 | 0.03467 | 0.00702 | 0.05408 | 0.00390 | 0.05902 | -0.08186 |
| Malignancy: Yes | **-0.04910** | **-0.03807** | **-0.01** | -0.09 | 0.04687 | -0.01930 | 0.04381 | -0.03467 | -0.00702 | -0.05408 | -0.00390 | -0.05902 | 0.08186 |
| Depressive disorders: No | **0.01169** | **0.00211** | **-0.00** | 0.01 | 0.04954 | -0.06561 | -0.02030 | 0.00310 | -0.02142 | -0.04125 | 0.11890 | -0.02826 | -0.00462 |
| Depressive disorders: Yes | **-0.01169** | **-0.00211** | **0.00** | -0.01 | -0.04954 | 0.06561 | 0.02030 | -0.00310 | 0.02142 | 0.04125 | -0.11890 | 0.02826 | 0.00462 |
| Asthma: No | **0.03493** | **0.00364** | **-0.02** | -0.04 | -0.02194 | -0.05770 | 0.10611 | -0.06585 | -0.06281 | 0.06083 | -0.04222 | -0.04496 | -0.00921 |
| Asthma: Yes | **-0.03493** | **-0.00364** | **0.02** | 0.04 | 0.02194 | 0.05770 | -0.10611 | 0.06585 | 0.06281 | -0.06083 | 0.04222 | 0.04496 | 0.00921 |
| Respiratory medications: No | **0.19992** | **0.16903** | **0.01** | 0.03 | 0.09358 | -0.00323 | -0.00339 | -0.01463 | 0.14971 | 0.00448 | 0.01318 | -0.05991 | -0.06323 |
| Respiratory medications: Yes | **-0.19992** | **-0.16903** | **-0.01** | -0.03 | -0.09358 | 0.00323 | 0.00339 | 0.01463 | -0.14971 | -0.00448 | -0.01318 | 0.05991 | 0.06323 |
| Inhaled short-acting muscarinic antagonists (SAMAs): No | **0.02031** | **0.01145** | **0.00** | 0.00 | 0.05448 | -0.00343 | 0.07597 | 0.03326 | -0.01213 | 0.00643 | -0.09157 | -0.04570 | 0.03167 |
| Inhaled short-acting muscarinic antagonists (SAMAs): Yes | **-0.02031** | **-0.01145** | **-0.02** | N.E. | -0.05448 | 0.00343 | -0.07597 | -0.03326 | 0.01213 | -0.00643 | N.E. | 0.04570 | -0.03167 |
| Inhaled long-acting muscarinic antagonists (LAMAs): No | **0.27505** | **0.23672** | **0.00** | 0.01 | 0.07590 | -0.01575 | 0.06833 | -0.02307 | 0.03218 | -0.00916 | -0.03987 | -0.07853 | 0.01341 |
| Inhaled long-acting muscarinic antagonists (LAMAs): Yes | **-0.27505** | **-0.23672** | **-0.00** | -0.01 | -0.07590 | 0.01575 | -0.06833 | 0.02307 | -0.03218 | 0.00916 | 0.03987 | 0.07853 | -0.01341 |
| Inhaled short-acting beta2-agonists (SABAs): No | **0.07103** | **0.05939** | **0.01** | 0.05 | 0.10959 | -0.07327 | -0.07642 | 0.01069 | 0.02937 | 0.05763 | -0.03169 | -0.07446 | 0.06354 |
| Inhaled short-acting beta2-agonists (SABAs): Yes | **-0.07103** | **-0.05939** | **-0.01** | -0.05 | -0.10959 | 0.07327 | 0.07642 | -0.01069 | -0.02937 | -0.05763 | 0.03169 | 0.07446 | -0.06354 |
| Inhaled glucocorticosteroids (ICS): No | **0.08337** | **0.05623** | **0.01** | -0.04 | 0.15652 | -0.03851 | 0.07936 | -0.17643 | 0.06006 | 0.06775 | -0.12283 | 0.09368 | 0.01320 |
| Inhaled glucocorticosteroids (ICS): Yes | **-0.08337** | **-0.05623** | **-0.01** | 0.04 | -0.15652 | 0.03851 | -0.07936 | 0.17643 | -0.06006 | -0.06775 | 0.12283 | -0.09368 | -0.01320 |
| Fixed combinations of SABA and SAMA: No | **0.14542** | **0.04790** | **-0.02** | -0.04 | N.E. | -0.02364 | -0.05723 | -0.01193 | 0.05169 | -0.03583 | -0.01513 | -0.11120 | 0.07399 |
| Fixed combinations of SABA and SAMA: Yes | **-0.14542** | **-0.04790** | **0.02** | N.E. | N.E. | N.E. | 0.05723 | 0.01193 | -0.05169 | 0.03583 | 0.01513 | 0.11120 | -0.07399 |
| Systemic glucocorticosteroids: No | **0.08811** | **0.05510** | **-0.00** | -0.03 | 0.05404 | -0.04180 | -0.06620 | 0.10589 | 0.20005 | -0.10963 | -0.04654 | -0.02592 | -0.05973 |
| Systemic glucocorticosteroids: Yes | **-0.08811** | **-0.05510** | **0.00** | 0.03 | -0.05404 | 0.04180 | 0.06620 | -0.10589 | -0.20005 | 0.10963 | 0.04654 | 0.02592 | 0.05973 |
| Systemic beta2-agonists: No | **0.02925** | **0.01004** | **-0.03** | 0.00 | N.E. | -0.03344 | 0.01181 | -0.04550 | -0.01213 | -0.06911 | -0.00910 | -0.08649 | N.E. |
| Systemic beta2-agonists: Yes | **-0.02925** | **-0.01004** | **0.00** | N.E. | N.E. | N.E. | -0.01181 | N.E. | 0.01213 | N.E. | 0.00910 | N.E. | N.E. |
| Xanthines and adrenergics: No | **-0.00831** | **-0.01177** | **-0.02** | -0.03 | -0.06508 | 0.00677 | 0.01625 | -0.00389 | 0.00258 | N.E. | -0.06462 | N.E. | N.E. |
| Xanthines and adrenergics: Yes | **0.00831** | **0.01177** | **0.00** | 0.03 | N.E. | -0.00677 | -0.01625 | 0.00389 | -0.00258 | N.E. | N.E. | N.E. | N.E. |
| Roflumilast: No | **0.22370** | **N.E.** | **0.00** | 0.00 | N.E. | N.E. | N.E. | N.E. | N.E. | N.E. | N.E. | N.E. | N.E. |
| Roflumilast: Yes | **-0.22370** | **N.E.** | **N.E.** | N.E. | N.E. | N.E. | N.E. | N.E. | N.E. | N.E. | N.E. | N.E. | N.E. |
| Nasal glucocorticosteroids: No | **-0.00121** | **-0.00121** | **0.00** | 0.01 | 0.06226 | -0.00857 | 0.09577 | -0.09440 | -0.01488 | 0.05955 | 0.02577 | -0.07940 | -0.04295 |
| Nasal glucocorticosteroids: Yes | **0.00121** | **0.00121** | **-0.00** | -0.01 | -0.06226 | 0.00857 | -0.09577 | 0.09440 | 0.01488 | -0.05955 | -0.02577 | 0.07940 | 0.04295 |
| Leukotriene receptor antagonists: No | **0.03191** | **0.02923** | **0.03** | 0.11 | 0.10149 | 0.05048 | -0.03321 | -0.01765 | -0.01213 | 0.10703 | 0.06799 | 0.00583 | -0.08156 |
| Leukotriene receptor antagonists: Yes | **-0.03191** | **-0.02923** | **-0.03** | -0.11 | -0.10149 | -0.05048 | 0.03321 | 0.01765 | 0.01213 | -0.10703 | -0.06799 | -0.00583 | 0.08156 |
| Oxygen therapy: No | **0.09184** | **0.06170** | **0.00** | -0.03 | 0.08277 | 0.01791 | -0.15880 | 0.00203 | 0.01131 | 0.03455 | -0.00242 | 0.09470 | -0.04697 |
| Oxygen therapy: Yes | **-0.09184** | **-0.06170** | **-0.00** | 0.03 | -0.08277 | -0.01791 | 0.15880 | -0.00203 | -0.01131 | -0.03455 | 0.00242 | -0.09470 | 0.04697 |
| Nebuliser therapy: No | **0.05632** | **N.E.** | **0.00** | 0.00 | N.E. | N.E. | N.E. | N.E. | N.E. | N.E. | N.E. | N.E. | N.E. |
| Nebuliser therapy: Yes | **-0.05632** | **N.E.** | **N.E.** | N.E. | N.E. | N.E. | N.E. | N.E. | N.E. | N.E. | N.E. | N.E. | N.E. |
| Cardiovascular medications: No | **0.01570** | **0.00549** | **-0.00** | -0.09 | 0.04744 | -0.00622 | -0.11986 | -0.02863 | 0.03884 | 0.03404 | -0.06093 | 0.05905 | 0.09765 |
| Cardiovascular medications: Yes | **-0.01570** | **-0.00549** | **0.00** | 0.09 | -0.04744 | 0.00622 | 0.11986 | 0.02863 | -0.03884 | -0.03404 | 0.06093 | -0.05905 | -0.09765 |
| Cardiac glycosides and antiarrhythmics, Class I and III: No | **0.06681** | **0.05033** | **0.02** | 0.07 | -0.00450 | -0.03659 | 0.04736 | 0.01763 | 0.08600 | -0.06151 | 0.05051 | 0.12013 | -0.12327 |
| Cardiac glycosides and antiarrhythmics, Class I and III: Yes | **-0.06681** | **-0.05033** | **-0.02** | -0.07 | 0.00450 | 0.03659 | -0.04736 | -0.01763 | -0.08600 | 0.06151 | -0.05051 | -0.12013 | 0.12327 |
| Vasodilators used in cardiac diseases: No | **0.01785** | **0.01289** | **0.00** | -0.02 | -0.04603 | -0.00691 | -0.01238 | -0.02711 | 0.03450 | 0.02128 | 0.08721 | -0.02099 | 0.01379 |
| Vasodilators used in cardiac diseases: Yes | **-0.01785** | **-0.01289** | **-0.00** | 0.02 | 0.04603 | 0.00691 | 0.01238 | 0.02711 | -0.03450 | -0.02128 | -0.08721 | 0.02099 | -0.01379 |
| Cardiac stimulants and other cardiac preparations: No | **0.03663** | **0.01780** | **-0.00** | 0.00 | -0.03063 | 0.02289 | -0.02508 | 0.01200 | -0.04975 | 0.01598 | 0.09768 | 0.02071 | -0.07851 |
| Cardiac stimulants and other cardiac preparations: Yes | **-0.03663** | **-0.01780** | **-0.02** | N.E. | N.E. | -0.02289 | N.E. | -0.01200 | N.E. | -0.01598 | -0.09768 | -0.02071 | 0.07851 |
| Diuretics: No | **0.06715** | **0.03879** | **0.01** | -0.14 | 0.00157 | -0.05754 | -0.06589 | -0.05210 | -0.02622 | 0.01815 | 0.14674 | 0.18555 | 0.05568 |
| Diuretics: Yes | **-0.06715** | **-0.03879** | **-0.01** | 0.14 | -0.00157 | 0.05754 | 0.06589 | 0.05210 | 0.02622 | -0.01815 | -0.14674 | -0.18555 | -0.05568 |
| Peripheral vasodilators: No | **-0.01334** | **N.E.** | **0.00** | 0.00 | N.E. | N.E. | N.E. | N.E. | N.E. | N.E. | N.E. | N.E. | N.E. |
| Peripheral vasodilators: Yes | **N.E.** | **N.E.** | **N.E.** | N.E. | N.E. | N.E. | N.E. | N.E. | N.E. | N.E. | N.E. | N.E. | N.E. |
| Vasoprotective agents: No | **0.01598** | **0.00427** | **-0.00** | 0.19 | -0.03903 | -0.03160 | -0.10348 | 0.01951 | 0.05233 | -0.02516 | -0.05612 | 0.06080 | -0.08178 |
| Vasoprotective agents: Yes | **-0.01598** | **-0.00427** | **0.00** | -0.19 | 0.03903 | 0.03160 | 0.10348 | -0.01951 | -0.05233 | 0.02516 | 0.05612 | -0.06080 | 0.08178 |
| Beta blocking agents: No | **0.01489** | **0.01947** | **0.01** | 0.03 | 0.04553 | -0.05461 | 0.01162 | -0.08226 | 0.06016 | -0.00007 | -0.03046 | 0.04938 | 0.10869 |
| Beta blocking agents: Yes | **-0.01489** | **-0.01947** | **-0.01** | -0.03 | -0.04553 | 0.05461 | -0.01162 | 0.08226 | -0.06016 | 0.00007 | 0.03046 | -0.04938 | -0.10869 |
| Calcium channel blockers: No | **0.01204** | **0.00163** | **-0.00** | -0.08 | -0.02342 | -0.05736 | -0.11875 | 0.02966 | -0.04200 | 0.02958 | 0.09512 | -0.08165 | 0.24212 |
| Calcium channel blockers: Yes | **-0.01204** | **-0.00163** | **0.00** | 0.08 | 0.02342 | 0.05736 | 0.11875 | -0.02966 | 0.04200 | -0.02958 | -0.09512 | 0.08165 | -0.24212 |
| Antihypertensives: No | **0.04995** | **0.01206** | **0.01** | -0.02 | 0.01687 | 0.12292 | 0.13569 | -0.10266 | -0.05610 | -0.05760 | -0.01522 | -0.02439 | 0.06046 |
| Antihypertensives: Yes | **-0.04995** | **-0.01206** | **-0.01** | N.E. | -0.01687 | -0.12292 | -0.13569 | 0.10266 | 0.05610 | 0.05760 | 0.01522 | 0.02439 | -0.06046 |
| Agents acting on the renin-angiotensin system: No | **-0.02505** | **-0.02312** | **-0.00** | -0.05 | 0.02956 | 0.01928 | -0.09076 | -0.07361 | -0.02131 | -0.02793 | -0.00605 | 0.12537 | 0.08563 |
| Agents acting on the renin-angiotensin system: Yes | **0.02505** | **0.02312** | **0.00** | 0.05 | -0.02956 | -0.01928 | 0.09076 | 0.07361 | 0.02131 | 0.02793 | 0.00605 | -0.12537 | -0.08563 |
| Angiotensin-converting-enzyme inhibitors: No | **-0.04085** | **-0.03094** | **0.01** | -0.05 | 0.04313 | -0.01155 | -0.08614 | -0.02012 | -0.00053 | -0.02527 | -0.04717 | 0.12468 | 0.14362 |
| Angiotensin-converting-enzyme inhibitors: Yes | **0.04085** | **0.03094** | **-0.01** | 0.05 | -0.04313 | 0.01155 | 0.08614 | 0.02012 | 0.00053 | 0.02527 | 0.04717 | -0.12468 | -0.14362 |
| Angiotensin II receptor antagonists: No | **0.00065** | **0.00121** | **-0.00** | -0.04 | 0.00866 | 0.04253 | -0.01873 | -0.06865 | -0.02573 | -0.00976 | 0.04635 | 0.05567 | -0.00759 |
| Angiotensin II receptor antagonists: Yes | **-0.00065** | **-0.00121** | **0.00** | 0.04 | -0.00866 | -0.04253 | 0.01873 | 0.06865 | 0.02573 | 0.00976 | -0.04635 | -0.05567 | 0.00759 |
| Renin-inhibitors: No | **-0.00897** | **-0.00148** | **123254.87** | 246509.78 | N.E. | -0.04731 | N.E. | N.E. | N.E. | N.E. | N.E. | N.E. | N.E. |
| Renin-inhibitors: Yes | **0.00897** | **0.00148** | **N.E.** | N.E. | N.E. | N.E. | N.E. | N.E. | N.E. | N.E. | N.E. | N.E. | N.E. |
| Lipid-modifying agents: No | **-0.05184** | **-0.03499** | **-0.00** | -0.09 | -0.00308 | 0.08196 | -0.00772 | -0.05539 | 0.00612 | -0.05602 | -0.12086 | 0.09211 | 0.12577 |
| Lipid-modifying agents: Yes | **0.05184** | **0.03499** | **0.00** | 0.09 | 0.00308 | -0.08196 | 0.00772 | 0.05539 | -0.00612 | 0.05602 | 0.12086 | -0.09211 | -0.12577 |
| HMG-CoA reductase inhibitors (statins): No | **-0.05130** | **-0.03252** | **-0.00** | -0.08 | 0.00747 | 0.08487 | -0.02828 | -0.06158 | 0.01441 | -0.03329 | -0.12796 | 0.09472 | 0.12714 |
| HMG-CoA reductase inhibitors (statins): Yes | **0.05130** | **0.03252** | **0.00** | 0.08 | -0.00747 | -0.08487 | 0.02828 | 0.06158 | -0.01441 | 0.03329 | 0.12796 | -0.09472 | -0.12714 |
| Other lipid-modifying agents: No | **-0.00885** | **-0.01311** | **-0.01** | -0.03 | 0.00330 | -0.09546 | 0.13934 | -0.02470 | -0.01332 | -0.06581 | -0.02048 | 0.02071 | 0.02237 |
| Other lipid-modifying agents: Yes | **0.00885** | **0.01311** | **0.01** | 0.03 | -0.00330 | 0.09546 | -0.13934 | 0.02470 | 0.01332 | 0.06581 | 0.02048 | -0.02071 | -0.02237 |
| Antithrombotic agents: No | **0.04008** | **0.02296** | **-0.01** | -0.06 | 0.09948 | -0.02114 | 0.02334 | 0.02386 | -0.12166 | -0.06102 | -0.02350 | 0.07813 | -0.00100 |
| Antithrombotic agents: Yes | **-0.04008** | **-0.02296** | **0.01** | 0.06 | -0.09948 | 0.02114 | -0.02334 | -0.02386 | 0.12166 | 0.06102 | 0.02350 | -0.07813 | 0.00100 |
| Platelet aggregation inhibitors: No | **0.01878** | **0.00638** | **-0.00** | -0.06 | 0.03128 | 0.06016 | 0.04126 | 0.04274 | -0.12523 | -0.07767 | -0.01480 | 0.01891 | 0.03912 |
| Platelet aggregation inhibitors: Yes | **-0.01878** | **-0.00638** | **0.00** | 0.06 | -0.03128 | -0.06016 | -0.04126 | -0.04274 | 0.12523 | 0.07767 | 0.01480 | -0.01891 | -0.03912 |
| Systemic antibacterials: No | **0.10705** | **0.07534** | **-0.00** | 0.08 | 0.13723 | -0.02827 | -0.08056 | -0.07020 | 0.17177 | -0.04533 | -0.10926 | -0.01800 | -0.05467 |
| Systemic antibacterials: Yes | **-0.10705** | **-0.07534** | **0.00** | -0.08 | -0.13723 | 0.02827 | 0.08056 | 0.07020 | -0.17177 | 0.04533 | 0.10926 | 0.01800 | 0.05467 |
| Iron preparations: No | **0.05208** | **0.02398** | **0.01** | -0.06 | 0.05686 | -0.02130 | 0.00216 | -0.06119 | 0.11015 | 0.00091 | 0.07470 | -0.00729 | -0.04440 |
| Iron preparations: Yes | **-0.05208** | **-0.02398** | **-0.01** | 0.06 | -0.05686 | 0.02130 | -0.00216 | 0.06119 | -0.11015 | -0.00091 | -0.07470 | 0.00729 | 0.04440 |
| Proton pump inhibitors: No | **0.06822** | **0.03240** | **-0.02** | 0.06 | -0.00879 | -0.03365 | -0.01878 | 0.03440 | -0.06488 | -0.01259 | -0.08467 | 0.01048 | -0.03772 |
| Proton pump inhibitors: Yes | **-0.06822** | **-0.03240** | **0.02** | -0.06 | 0.00879 | 0.03365 | 0.01878 | -0.03440 | 0.06488 | 0.01259 | 0.08467 | -0.01048 | 0.03772 |
| Drugs used in diabetes: No | **0.03406** | **0.02148** | **0.01** | -0.10 | 0.00315 | 0.02789 | -0.12108 | 0.13428 | -0.02500 | 0.08710 | 0.00911 | 0.06189 | 0.00036 |
| Drugs used in diabetes: Yes | **-0.03406** | **-0.02148** | **-0.01** | 0.10 | -0.00315 | -0.02789 | 0.12108 | -0.13428 | 0.02500 | -0.08710 | -0.00911 | -0.06189 | -0.00036 |
| Insulins: No | **0.04346** | **0.02639** | **0.01** | 0.02 | 0.05707 | 0.01052 | 0.02144 | 0.00981 | -0.09120 | 0.14892 | -0.02294 | 0.00655 | -0.06307 |
| Insulins: Yes | **-0.04346** | **-0.02639** | **-0.01** | -0.02 | -0.05707 | -0.01052 | -0.02144 | -0.00981 | 0.09120 | -0.14892 | 0.02294 | -0.00655 | 0.06307 |
| Blood glucose–lowering drugs: No | **0.02395** | **0.01601** | **0.01** | -0.11 | -0.00023 | 0.02574 | -0.13269 | 0.15348 | -0.00210 | 0.06587 | -0.01287 | 0.11138 | -0.01216 |
| Blood glucose–lowering drugs: Yes | **-0.02395** | **-0.01601** | **-0.01** | 0.11 | 0.00023 | -0.02574 | 0.13269 | -0.15348 | 0.00210 | -0.06587 | 0.01287 | -0.11138 | 0.01216 |
| Drugs for musculoskeletal system: No | **-0.00941** | **-0.00727** | **0.00** | 0.05 | 0.11049 | -0.06377 | 0.01258 | -0.14302 | -0.07980 | 0.05331 | 0.01272 | -0.04608 | 0.12489 |
| Drugs for musculoskeletal system: Yes | **0.00941** | **0.00727** | **-0.00** | -0.05 | -0.11049 | 0.06377 | -0.01258 | 0.14302 | 0.07980 | -0.05331 | -0.01272 | 0.04608 | -0.12489 |
| Anti-inflammatory and antirheumatic products, non-steroids (non-steroidal anti-inflammatory drugs): | **-0.01443** | **-0.00920** | **0.01** | 0.05 | 0.10562 | -0.06815 | 0.02722 | -0.12934 | -0.08765 | 0.05107 | 0.04875 | -0.04867 | 0.09961 |
| Anti-inflammatory and antirheumatic products, non-steroids (non-steroidal anti-inflammatory drugs): | **0.01443** | **0.00920** | **-0.01** | -0.05 | -0.10562 | 0.06815 | -0.02722 | 0.12934 | 0.08765 | -0.05107 | -0.04875 | 0.04867 | -0.09961 |
| Acetylsalicylic acid (other analgesics and antipyretics): No | **0.03090** | **0.00409** | **-0.01** | -0.04 | 0.00540 | 0.04474 | -0.07109 | -0.08328 | 0.06187 | 0.01400 | -0.09443 | -0.03169 | 0.08732 |
| Acetylsalicylic acid (other analgesics and antipyretics): Yes | **-0.03090** | **-0.00409** | **-0.01** | N.E. | -0.00540 | -0.04474 | N.E. | N.E. | -0.06187 | -0.01400 | 0.09443 | 0.03169 | -0.08732 |
| Antidepressants: No | **0.07108** | **0.03715** | **-0.01** | 0.03 | 0.08724 | 0.00704 | -0.03588 | -0.00427 | -0.03140 | -0.01249 | -0.10214 | -0.03129 | 0.04087 |
| Antidepressants: Yes | **-0.07108** | **-0.03715** | **0.01** | -0.03 | -0.08724 | -0.00704 | 0.03588 | 0.00427 | 0.03140 | 0.01249 | 0.10214 | 0.03129 | -0.04087 |
| Selective serotonin reuptake inhibitors: No | **0.05444** | **0.02621** | **-0.01** | -0.01 | 0.01890 | -0.06850 | -0.03331 | -0.02841 | 0.02429 | -0.06803 | -0.01738 | 0.02551 | 0.08953 |
| Selective serotonin reuptake inhibitors: Yes | **-0.05444** | **-0.02621** | **0.01** | 0.01 | -0.01890 | 0.06850 | 0.03331 | 0.02841 | -0.02429 | 0.06803 | 0.01738 | -0.02551 | -0.08953 |
| Antineoplastic agents: No | **0.00427** | **-0.00209** | **-0.04** | 0.00 | N.E. | -0.04096 | N.E. | N.E. | -0.04061 | N.E. | N.E. | -0.06105 | N.E. |
| Antineoplastic agents: Yes | **-0.00427** | **0.00209** | **N.E.** | N.E. | N.E. | N.E. | N.E. | N.E. | N.E. | N.E. | N.E. | N.E. | N.E. |
| Immunosuppressants: No | **0.03001** | **0.02710** | **0.01** | 0.00 | 0.06410 | 0.09060 | 0.03804 | 0.01950 | 0.02787 | 0.05352 | -0.02603 | -0.04867 | -0.09353 |
| Immunosuppressants: Yes | **-0.03001** | **-0.02710** | **-0.01** | -0.00 | -0.06410 | -0.09060 | -0.03804 | -0.01950 | -0.02787 | -0.05352 | 0.02603 | 0.04867 | 0.09353 |
| Antivirals for systemic use: No | **-0.01463** | **-0.00424** | **0.00** | 0.09 | 0.04489 | 0.16795 | -0.07025 | -0.03200 | 0.06240 | -0.04990 | -0.03597 | -0.05616 | -0.06889 |
| Antivirals for systemic use: Yes | **0.01463** | **0.00424** | **-0.00** | -0.09 | -0.04489 | -0.16795 | 0.07025 | 0.03200 | -0.06240 | 0.04990 | 0.03597 | 0.05616 | 0.06889 |
| Hormone-replacement therapy: Estrogens, progestogens, progestogens and estrogens in combination: No | **-0.03790** | **-0.02343** | **0.01** | -0.06 | 0.03776 | 0.00848 | 0.02117 | -0.07834 | 0.15181 | 0.01457 | -0.17269 | 0.00611 | 0.17328 |
| Hormone-replacement therapy: Estrogens, progestogens, progestogens and estrogens in combination: Yes | **0.03790** | **0.02343** | **-0.01** | 0.06 | -0.03776 | -0.00848 | -0.02117 | 0.07834 | -0.15181 | -0.01457 | 0.17269 | -0.00611 | -0.17328 |
| Drugs used in nicotine dependence: No | **-0.01198** | **-0.02604** | **-0.01** | -0.00 | 0.07073 | -0.06677 | -0.01409 | 0.01951 | -0.04948 | -0.01816 | -0.08928 | -0.01304 | 0.03069 |
| Drugs used in nicotine dependence: Yes | **0.01198** | **0.02604** | **0.01** | 0.00 | -0.07073 | 0.06677 | 0.01409 | -0.01951 | 0.04948 | 0.01816 | 0.08928 | 0.01304 | -0.03069 |
| COPD severity: mild | **0.20566** | **0.17376** | **0.01** | 0.01 | 0.02687 | 0.01630 | -0.01039 | -0.12235 | 0.16373 | 0.07106 | -0.10548 | 0.04135 | -0.01646 |
| COPD severity: moderate | **-0.02484** | **-0.03142** | **0.00** | 0.01 | 0.09250 | 0.06696 | -0.05513 | 0.08795 | 0.00638 | 0.00142 | -0.16650 | -0.01639 | -0.01838 |
| COPD severity: severe | **-0.08014** | **-0.06348** | **-0.01** | -0.08 | -0.06678 | -0.05472 | -0.01839 | 0.02653 | -0.09597 | 0.00899 | 0.18433 | 0.05458 | -0.05548 |
| COPD severity: very severe | **-0.12470** | **-0.09482** | **-0.01** | 0.08 | -0.09653 | -0.02861 | 0.08341 | -0.03374 | -0.03163 | -0.06184 | 0.01331 | -0.06420 | 0.07350 |
| CCI score: 1 | **0.03624** | **0.024178** | **-0.00** | 0.01 | 0.06152 | -0.00185 | -0.04050 | 0.04096 | -0.10009 | 0.01502 | 0.00535 | 0.01804 | -0.04636 |
| CCI score: 2 | **0.06275** | **0.047525** | **0.01** | 0.06 | -0.06043 | 0.06966 | 0.03169 | -0.06786 | -0.05165 | 0.02425 | -0.00788 | 0.13441 | -0.04311 |
| CCI score: 3+ | **-0.08548** | **-0.061479** | **-0.01** | -0.07 | -0.01115 | -0.05623 | 0.01317 | 0.01523 | 0.13205 | -0.03237 | 0.00043 | -0.11852 | 0.07080 |
| Hip fracture: No | **0.01388** | **0.007626** | **0.01** | -0.04 | N.E. | 0.04380 | -0.12635 | 0.01583 | 0.08754 | 0.04595 | 0.00034 | 0.03151 | 0.02237 |
| Hip fracture: Yes | **-0.01388** | **-0.007626** | **-0.03** | 0.04 | N.E. | -0.04380 | N.E. | -0.01583 | -0.08754 | -0.04595 | -0.00034 | -0.03151 | -0.02237 |
| Lung cancer: No | **0.09243** | **0.097959** | **24,651.02** | 246,509.78 | -0.08414 | 0.10474 | -0.07075 | 0.20651 | -0.01554 | 0.09869 | -0.02176 | 0.05501 | 0.10555 |
| Lung cancer: Yes | **-0.09243** | **-0.097959** | **-0.06** | N.E. | N.E. | -0.10474 | 0.07075 | -0.20651 | 0.01554 | -0.09869 | 0.02176 | -0.05501 | -0.10555 |
| Other markers of bad fall: No | **-0.01466** | **-0.005109** | **-0.00** | 0.02 | -0.04846 | -0.03989 | 0.01881 | 0.01200 | 0.03321 | 0.02263 | -0.04294 | N.E. | 0.02742 |
| Other markers of bad fall: Yes | **0.01466** | **0.005109** | **-0.01** | -0.02 | N.E. | 0.03989 | -0.01881 | -0.01200 | -0.03321 | -0.02263 | 0.04294 | N.E. | -0.02742 |
| Metastatic cancer: No | **0.03195** | **0.051061** | **73952.96** | 739529.34 | -0.03752 | 0.02164 | 0.02301 | 0.19426 | -0.05610 | 0.08196 | 0.04877 | 0.03983 | -0.07341 |
| Metastatic cancer: Yes | **-0.03195** | **-0.051061** | **-0.03** | N.E. | N.E. | -0.02164 | -0.02301 | -0.19426 | 0.05610 | -0.08196 | -0.04877 | -0.03983 | 0.07341 |
| Pulmonary cachexia: No | **0.01440** | **0.018167** | **0.05** | 0.00 | 0.33933 | 0.19742 | 0.03764 | -0.02013 | N.E. | 0.00216 | N.E. | -0.04570 | -0.08755 |
| Pulmonary cachexia: Yes | **-0.01440** | **-0.018167** | **-0.05** | -0.00 | -0.33933 | -0.19742 | -0.03764 | 0.02013 | N.E. | -0.00216 | N.E. | 0.04570 | 0.08755 |
| Right-sided heart failure: No | **0.01783** | **0.027623** | **0.03** | -0.07 | -0.02489 | 0.12292 | 0.02820 | 0.08118 | 0.05492 | -0.03134 | 0.05197 | -0.02583 | 0.08831 |
| Right-sided heart failure: Yes | **-0.01783** | **-0.027623** | **-0.04** | N.E. | 0.02489 | -0.12292 | -0.02820 | -0.08118 | -0.05492 | 0.03134 | -0.05197 | 0.02583 | -0.08831 |
| Number of hospitalisations within 180 days: 0 | **0.13801** | **0.086297** | **0.00** | -0.06 | 0.06619 | 0.09822 | -0.00974 | 0.04585 | -0.11652 | -0.08388 | 0.06165 | 0.00355 | 0.01796 |
| Number of hospitalisations within 180 days: 1 | **-0.06096** | **-0.038699** | **0.01** | 0.11 | -0.07398 | -0.04477 | 0.09555 | -0.09427 | 0.05688 | -0.00383 | -0.04489 | 0.02925 | 0.06445 |
| Number of hospitalisations within 180 days: 2 | **-0.10459** | **-0.077585** | **-0.04** | -0.18 | -0.00518 | -0.15484 | -0.11624 | -0.00151 | 0.09237 | 0.13914 | 0.00087 | -0.11675 | -0.08104 |
| Number of hospitalisations within 180 days: 3-4 | **-0.10817** | **-0.053876** | **-0.02** | -0.15 | -0.05448 | 0.04404 | -0.13542 | 0.04125 | 0.05290 | 0.05438 | -0.05767 | 0.06224 | -0.04869 |
| Number of hospitalisations within 180 days: 5+ | **-0.00495** | **-0.008291** | **-0.04** | N.E. | 0.04037 | -0.30795 | -0.08271 | 0.09927 | 0.00485 | -0.04419 | -0.02027 | 0.03343 | -0.05497 |
| Number of hospitalisations within 365 days: 0 | **0.13237** | **0.092053** | **0.01** | -0.03 | 0.05574 | -0.01284 | -0.02640 | 0.03932 | -0.00110 | 0.03254 | -0.03625 | -0.02991 | 0.09095 |
| Number of hospitalisations within 365 days: 1 | **-0.04162** | **-0.035504** | **-0.00** | 0.08 | -0.03323 | 0.07439 | 0.06328 | -0.02596 | -0.07566 | -0.04445 | -0.00514 | 0.00843 | -0.07679 |
| Number of hospitalisations within 365 days: 2 | **-0.04970** | **-0.039821** | **-0.01** | -0.09 | -0.05673 | -0.01791 | 0.00672 | -0.06140 | 0.07195 | 0.05213 | 0.03354 | 0.00978 | -0.02795 |
| Number of hospitalisations within 365 days: 3-4 | **-0.06829** | **-0.056725** | **-0.01** | -0.00 | -0.02128 | -0.04209 | -0.04270 | 0.00286 | 0.03767 | -0.08736 | 0.03534 | 0.03574 | -0.03565 |
| Number of hospitalisations within 365 days: 5+ | **-0.12603** | **-0.046613** | **-0.05** | N.E. | 0.05673 | -0.52900 | -0.09729 | 0.07183 | -0.02050 | 0.06844 | -0.00459 | -0.01748 | 0.03855 |
| Number of hospitalisations with COPD within 180 days: 0 | **0.09115** | **0.045763** | **-0.00** | 0.02 | 0.01132 | 0.10835 | -0.00901 | -0.11644 | 0.04159 | -0.04585 | -0.00906 | 0.00585 | -0.03451 |
| Number of hospitalisations with COPD within 180 days: 1 | **-0.08473** | **-0.046797** | **-0.00** | 0.01 | -0.03795 | -0.09975 | 0.01784 | 0.08137 | -0.09124 | 0.02054 | 0.03655 | 0.03588 | 0.01314 |
| Number of hospitalisations with COPD within 180 days: 2+ | **-0.03169** | **-0.005650** | **-0.01** | -0.11 | 0.03934 | -0.04606 | -0.02820 | 0.09578 | 0.07283 | 0.05760 | -0.10927 | -0.10725 | 0.05914 |
| Number of COPD exacerbations within 90 days: 0 | **0.07483** | **0.042676** | **-0.01** | 0.03 | 0.06789 | -0.02183 | -0.08572 | 0.02503 | 0.10604 | -0.11967 | -0.11592 | 0.01208 | 0.00374 |
| Number of COPD exacerbations within 90 days: 1 | **-0.02705** | **-0.020826** | **0.01** | -0.01 | -0.06296 | 0.04124 | 0.09533 | -0.00310 | -0.06026 | 0.01949 | -0.03439 | 0.05993 | 0.01290 |
| Number of COPD exacerbations within 90 days: 2 | **-0.08158** | **-0.030531** | **0.01** | -0.04 | -0.00151 | -0.03769 | 0.02683 | -0.02751 | -0.03177 | 0.13721 | 0.16801 | -0.08795 | -0.03573 |
| Number of COPD exacerbations within 90 days: 3+ | **-0.05619** | **-0.046813** | **-0.04** | -0.11 | -0.05926 | -0.00278 | -0.09729 | -0.05409 | -0.18624 | 0.08696 | 0.11805 | -0.09251 | 0.01621 |
| Number of COPD exacerbations within 180 days: 0 | **0.10486** | **0.071583** | **-0.00** | 0.09 | 0.07190 | -0.04213 | -0.08394 | -0.03000 | 0.20528 | -0.10688 | -0.03895 | -0.01402 | -0.09452 |
| Number of COPD exacerbations within 180 days: 1 | **-0.02634** | **-0.022620** | **0.00** | -0.08 | -0.02194 | 0.08792 | 0.09629 | 0.02764 | -0.10845 | -0.04937 | -0.13384 | 0.10808 | 0.10203 |
| Number of COPD exacerbations within 180 days: 2 | **-0.03309** | **-0.033044** | **-0.01** | -0.03 | -0.09889 | -0.01554 | 0.05032 | -0.02261 | -0.07590 | 0.13257 | 0.11912 | -0.07301 | -0.11749 |
| Number of COPD exacerbations within 180 days: 3+ | **-0.13472** | **-0.070706** | **-0.01** | -0.03 | 0.01611 | -0.10164 | -0.11139 | 0.03717 | -0.12783 | 0.11256 | 0.11344 | -0.05704 | 0.07982 |

CCI = Charlson Comorbidity Index; COPD = chronic obstructive pulmonary disease; HMG-CoA = hydroxymethylglutaryl-coenzyme A; ICS = inhaled glucocorticosteroid; LABA = long-acting beta2-agonist; LAMA = long-acting muscarinic antagonist; NE = not estimable; SABA = short-acting beta2-agonist; SAMA = short-acting muscarinic antagonist; STdiff Dx = standardised difference per each propensity score decile.

Note on colour codes:

ORANGE: Cells in orange indicate standardised bias ≥ 0.1 and < 0.2.

YELLOW: Cells in yellow indicate standardised bias ≥ 0.2.

Table S16. Key Baseline Characteristics of Patients in Four Cohorts: Overall Population, Users of FDC LABA/LAMA and LABA Naive, Users of FDC LABA/LAMA and LABA Naive With No Hospitalisations for COPD in the 90 Days Before the Index Date, and Users of FDC LABA/LAMA and LABA Naive Further Restricted and Propensity Score 10% Trimmed

|  | Overall population | | | | FDC LABA/LAMA, LABA naive | | | | FDC LABA/LAMA, LABA naive, with no COPD hospitalisations in the 90 days before the index date | | | | FDC LABA/LAMA, LABA naive 10% trimmed | | | |
| --- | --- | --- | --- | --- | --- | --- | --- | --- | --- | --- | --- | --- | --- | --- | --- | --- |
|  | Olodaterol cohort (N = 14,239) | | Other LABA cohort (N = 51,167) | | Olodaterol cohort (N = 5,677) | | Other LABA cohort (N=20,514) | | Olodaterol cohort (N = 3,843) | | Other LABA cohort (N=14,029) | | Olodaterol cohort (N=4,191) | | Other LABA cohort (N=11,504) | |
|  | N | % | N | % | N | % | N | % | N | % | N | % | N | % | N | % |
| Oxygen therapy: yes | 2,092 | 15 | 5,701 | 11 | 994 | 18 | 2,804 | 14 | 443 | 12 | 1,361 | 10 | 731 | 17 | 1,710 | 15 |
| COPD severity: severe | 6,651 | 47 | 20,444 | 40 | 2,400 | 42 | 7,258 | 35 | 1,183 | 31 | 3,919 | 28 | 1,771 | 42 | 4,406 | 38 |
| COPD severity: very severe | 3,339 | 23 | 9,272 | 18 | 1,458 | 26 | 4,321 | 21 | 816 | 21 | 2,472 | 18 | 1,097 | 26 | 2,701 | 23 |
| Number of COPD exacerbations within 90 days before the index date: 1 | 4,425 | 31 | 14,666 | 29 | 1,929 | 34 | 5,932 | 29 | 911 | 24 | 3,203 | 23 | 1,461 | 35 | 3,646 | 32 |
| Number of COPD exacerbations within 90 days before the index date: 2 | 2,354 | 17 | 5,787 | 11 | 864 | 15 | 2,210 | 11 | 196 | 5 | 627 | 4 | 613 | 15 | 1,273 | 11 |
| Number of COPD exacerbations within 90 days before the index date: 3 or more | 1,200 | 8 | 2,132 | 4 | 285 | 5 | 644 | 3 | 46 | 1 | 110 | 1 | 148 | 4 | 286 | 2 |
| Number of COPD hospitalisations within 90 days before the index date: 1 | 3,577 | 25 | 8,189 | 16 | 1,614 | 28 | 3,345 | 16 | None |  | None |  | 1,126 | 27 | 1,989 | 17 |
| Number of COPD hospitalisations within 90 days before the index date: 2 or more | 960 | 7 | 1,382 | 3 | 242 | 4 | 504 | 2 | None |  | None |  | 128 | 3 | 200 | 2 |
| Number of hospitalisations within 180 days before the index date: 1 | 4,029 | 28 | 12,143 | 24 | 1,871 | 33 | 5,236 | 26 | 680 | 18 | 2,282 | 16 | 1,382 | 33 | 3,290 | 29 |
| Hospitalisations within 180 days before the index date: 2 | 1,519 | 11 | 3,511 | 7 | 561 | 10 | 1,373 | 7 | 187 | 5 | 484 | 3 | 359 | 9 | 741 | 6 |
| Number of hospitalisations within 180 days before the index date: 3 to 4 | 963 | 7 | 1,874 | 4 | 321 | 6 | 732 | 4 | 83 | 2 | 211 | 2 | 159 | 4 | 299 | 3 |
| Number of hospitalisations within 180 days before the index date: 5 or more | 280 | 2 | 461 | 1 | 100 | 2 | 214 | 1 | 22 | 1 | 72 | 1 | 55 | 1 | 128 | 1 |

COPD = chronic obstructive pulmonary disease; FDC = fixed-dose combination; LABA = long-acting beta2-agonist; LAMA = long-acting muscarinic antagonist.

## Supplemental Figures

Figure S1. Overview of Study Design and Eligibility Criteria for the Study Cohorts


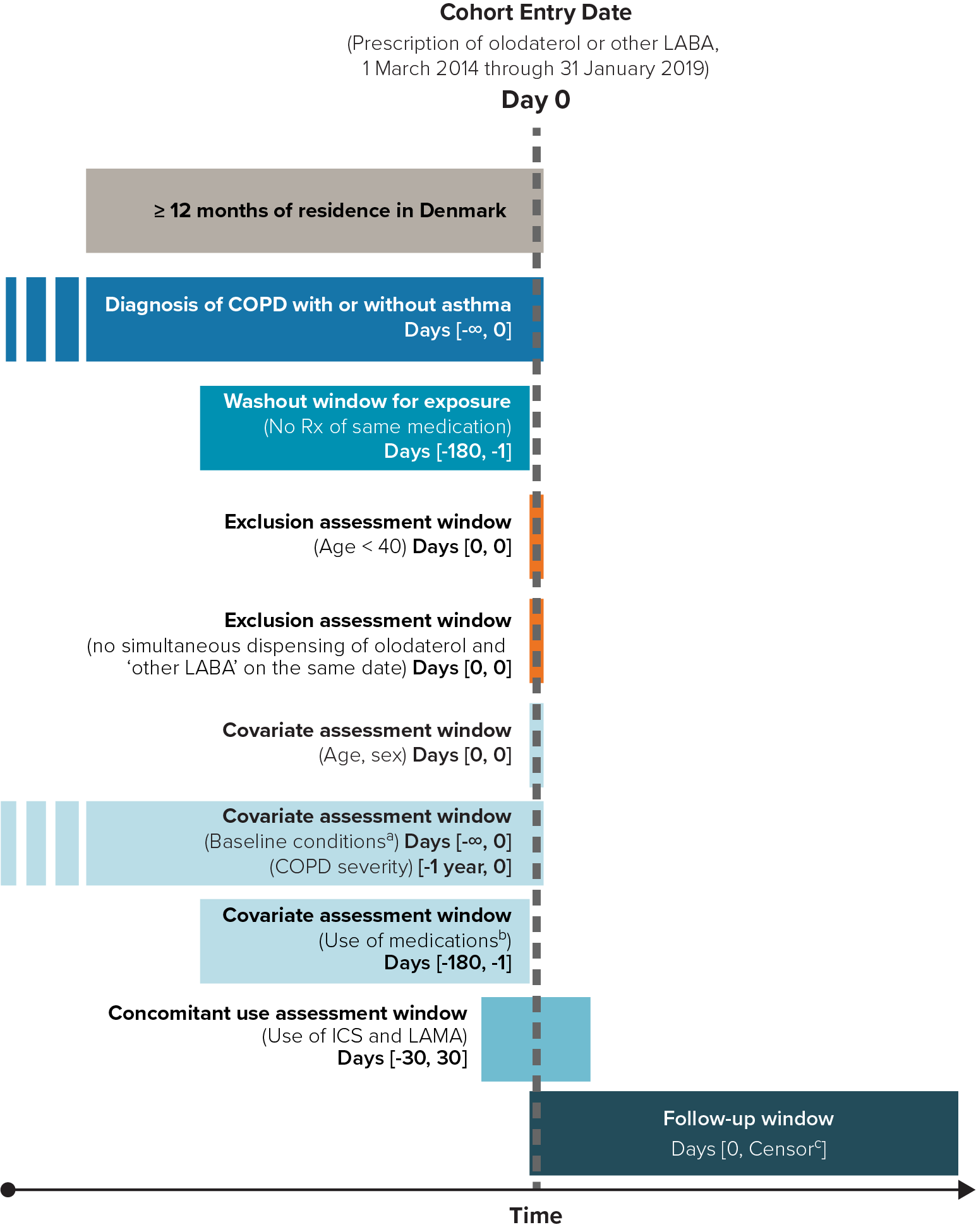


COPD = chronic obstructive pulmonary disease; ICS = inhaled glucocorticoids; LABA = long-acting beta2-agonist; LAMA = long-acting muscarinic antagonist.

^a^ Baseline conditions: cardiovascular disease, hyperlipidaemia, diabetes mellitus, renal disease, anaemias, peptic ulcer disease, liver disease, osteoporosis, rheumatoid arthritis and other inflammatory arthropathies, systemic connective tissue diseases, malignancies, and depressive disorders ever before index date. Pregnancy at index date. COPD severity based on the 12 months before the index date. Variables included in additional analysis: number of hospitalisations overall, within 180 days and 1 year before index date; number of hospitalisations because of COPD, within 90 and 180 days before index date; COPD exacerbations without hospitalisation, within 90 and 180 days before index date; Charlson Comorbidity Index based on disease diagnosis recorded any time before index date; and the following potential markers of frailty: hip fracture, lung cancer, other markers of a clinically important fall, metastatic cancer, pulmonary cachexia, and right-sided heart failure.

^b^ Use of respiratory and non-respiratory medications dispensed in the 180 days before index date (cardiovascular medications, lipid-lowering drugs, antithrombotic agents, systemic antibacterials, iron preparations, proton pump inhibitors, drugs used for diabetes, drugs for musculoskeletal system, non-steroidal anti-inflammatory and antirheumatic products, other antirheumatic agents, antidepressants, antineoplastic agents, immunosuppressants, antivirals for systemic use, hormone-replacement therapy, drugs used in nicotine dependence).

^c^ End of follow-up: earliest occurrence of outcome of interest, death, 14 days after estimated discontinuation of the last dispensing for the index LABA, addition of a second LABA, emigration, or end of the study period.

Source: The original design diagram template can be found at [www.repeatinitiative.org/projects.html](http://www.repeatinitiative.org/projects.html).

Figure S2. Distribution of Propensity Scores by Study Medication Cohort


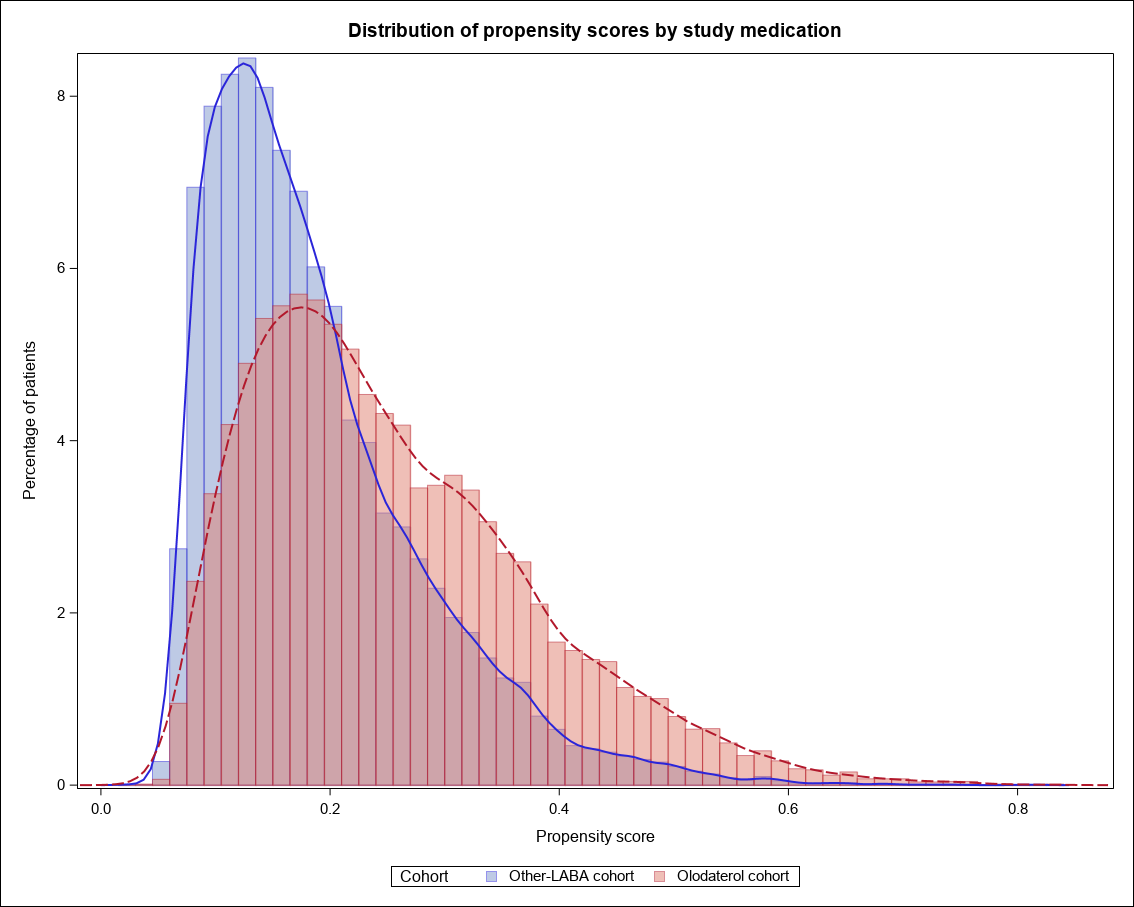


## Bias analysis

A bias analysis to evaluate under what conditions the observed association between olodaterol use and all-cause mortality could be explained by unmeasured confounding was conducted. The array-based method described by Schneeweiss (2006)^5^ was used. As described below, the method varies values for the prevalence of a putative unmeasured confounder between the cohorts and also varies the strength of association between the unmeasured confounder and the all-cause mortality outcome.

Using these inputs, the apparent adjusted relative rate (i.e., IRR) obtained from the study is further adjusted for the unmeasured confounder, according to the following formula:

, where

1. *ARR* is the apparent relative rate obtained from the main analysis, adjusting for measured covariates
2. *RR_CD_* is the relative risk of the association between unmeasured confounder and outcome
3. *P_C1_* is the prevalence of the unmeasured confounder in exposed cohort
4. *P_C0_* is the prevalence of the unmeasured confounder in the unexposed cohort
5. *RR_adj_*_._ is the value of IRR after adjusting for the effect of the unmeasured confounder

Several factors that predict mortality risk in COPD could not be ascertained from the data available for this study, including information about body mass index (BMI), smoking, subjective dyspnoea, and measures of airway obstruction (i.e., FEV_1_).^6-8^ We obtained estimates of the effect of these factors from results of the TIOSPIR (TIOtropium Safety and Performance In Respimat [TIOSPIR]) trial, which followed more than 17,000 patients with COPD randomised to different forms of tiotropium and followed for more than 2 years for a mortality outcome. We evaluated the potential effect of each of these variables measured at cohort entry. The relative risk of association between unmeasured confounder and outcome (*RR_CD_*) was estimated from the hazards ratios (HRs) reported by Calverley et al. (2017)^7^:

1. Very low BMI (< 18.5 vs. ≥ 18.5), HR = 2.8;
2. Severe airflow impairment (GOLD stage 4 vs. stages 3 or 2, reflecting decrements in predicted FEV_1_), HR = 2.0;
3. One-point difference in the modified Medical Research Council (mMRC) Dyspnea Scale, HR = 1.4;
4. Current smoking, HR = 1.2, and
5. History of heavy smoking (> 40 pack-years of cigarettes), HR = 1.2.

We present results from two different arrays, based on the adjusted IRR of all-cause mortality among the overall population, after adjusting the outcome model for additional variables, 1.40, as well as the adjusted IRR of all-cause mortality among the most restricted subpopulation (i.e., users of fixed-dose combination of LABA/LAMA who were LABA naive and had no hospitalisations for COPD in the 90 days prior to the index date; IRR = 1.26). Each assessment evaluated the effect of each potential unmeasured confounder across a range of empirically observed prevalence differences in the study data.

Table S16 presents the bias analysis that begins with an apparent IRR of 1.26, obtained from the analysis of the restricted cohort (Table 3). Table S16 illustrates sequential adjustment for each of the suspected unmeasured confounders, across four scenarios of confounder prevalence. In each set of scenarios, the prevalence is assumed to be 0.10 for the comparator cohort, while the prevalence for the exposed cohort ranges from 0.20 to 0.15. Differences in prevalence of this magnitude were observed for some covariates in empirical data for this subcohorts of the main study. For example, the proportion of patients with prior LAMA use was 37% (olodaterol cohort) and 27% (“other LABA” cohort). In several scenarios, including those that assumed a smaller difference in confounder prevalence, adjustment attenuated the IRR to values suggesting minimal or no elevated risk.

Table S17. Effect of Adjusting for Unmeasured Confounders Across a Range of Scenarios When the Apparent Relative Risk is 1.26

| Scenario set^a^  Apparent relative risk | Association between the unmeasured confounder and all-cause mortality (*RR_CD_*) | Prevalence in the olodaterol cohort (*P_C1_*) | Prevalence in the other LABA cohort (*P_C0_*) | Apparent IRR further adjusted for the unmeasured confounder | Confounder |
| --- | --- | --- | --- | --- | --- |
| 1 |  |  |  |  |  |
| 1.26 | 2.8 | 0.20 | 0.10 | 1.09 | Low BMI |
| 1.09 | 2.0 | 0.20 | 0.10 | 1.00 | GOLD stage 4 FEV1 vs. others |
| 1.00 | 1.4 | 0.20 | 0.10 | 0.97 | 1 point more on dyspnoea scale |
| 0.97 | 1.2 | 0.20 | 0.10 | 0.95 | Current smoker |
| 0.95 | 1.2 | 0.20 | 0.10 | 0.93 | Heavy former smoker |
| 2 |  |  |  |  |  |
| 1.26 | 2.8 | 0.18 | 0.10 | 1.12 | Low BMI |
| 1.12 | 2.0 | 0.18 | 0.10 | 1.05 | GOLD stage 4 FEV1 vs. others |
| 1.05 | 1.4 | 0.18 | 0.10 | 1.02 | 1 point more on dyspnoea scale |
| 1.02 | 1.2 | 0.18 | 0.10 | 1.00 | Current smoker |
| 1.00 | 1.2 | 0.18 | 0.10 | 0.98 | Heavy former smoker |
| 3 |  |  |  |  |  |
| 1.26 | 2.8 | 0.16 | 0.10 | 1.15 | Low BMI |
| 1.15 | 2 | 0.16 | 0.10 | 1  .09 | GOLD stage 4 FEV1 vs. others |
| 1.09 | 1.4 | 0.16 | 0.10 | 1.07 | 1 point more on dyspnoea scale |
| 1.07 | 1.2 | 0.16 | 0.10 | 1.06 | Current smoker |
| 1.06 | 1.2 | 0.16 | 0.10 | 1.05 | Heavy former smoker |

^a^In each set of scenarios, the apparent relative risk values that are indented represent partial adjustment by the factor in the row above.

Table S17 presents alternative sets of bias analyses that begin with an apparent IRR of 1.40, obtained from the adjusted analysis of the main study population (Table 3). Table S17 illustrates sequential adjustment for each of the suspected unmeasured confounders across three sets of scenarios of confounder prevalence. In each set of scenarios, the prevalence is assumed to be 0.10 for the comparator cohort, while the prevalence for the exposed cohort ranges from 0.22 to 0.18. Differences of this magnitude were empirically observed for some measured covariates. These differences were smaller than in this further restricted cohort than in the cohort users of fixed-dose combination of LABA/LAMA who were LABA naive. For example, the prevalence of prior LAMA use differed between cohort by 12% (51% vs. 39%). Similarly, prevalence of the combination of severe or very severe COPD also differed by this this magnitude (70% vs. 58%). In the first two sets of scenarios, when the prevalence differed by 12% and 10%, respectively, the fully adjusted IRRs indicated no elevated risk. In the final scenarios, which assumed a prevalence difference of 8%, the IRR was attenuated substantially but not completely. In summary, results from the bias analysis showed that residual confounding could readily account for much of the remaining association.

Table S18. Effect of Adjusting for Unmeasured Confounders Across a Range of Scenarios When the Apparent Relative Risk is 1.40

| Scenario set^a^  Apparent relative risk | Association between the unmeasured confounder and all-cause mortality (*RR_CD_*) | Prevalence in the olodaterol cohort (*P_C1_*) | Prevalence in the other LABA cohort (*P_C0_*) | Apparent IRR further adjusted for the unmeasured confounder | Confounder |
| --- | --- | --- | --- | --- | --- |
| 1 |  |  |  |  |  |
| 1.40 | 2.8 | 0.22 | 0.10 | 1.18 | Low BMI |
| 1.18 | 2 | 0.22 | 0.10 | 1.07 | GOLD stage 4 FEV1 vs. others |
| 1.07 | 1.4 | 0.22 | 0.10 | 1.02 | 1 point more on dyspnoea scale |
| 1.02 | 1.2 | 0.22 | 0.10 | 1.00 | Current smoker |
| 1.00 | 1.2 | 0.22 | 0.10 | 0.97 | Heavy former smoker |
| 2 |  |  |  |  |  |
| 1.40 | 2.8 | 0.20 | 0.10 | 1.21 | Low BMI |
| 1.21 | 2 | 0.20 | 0.10 | 1.11 | GOLD stage 4 FEV_1_ vs. others |
| 1.11 | 1.4 | 0.20 | 0.10 | 1.07 | 1 point more on dyspnoea scale |
| 1.07 | 1.2 | 0.20 | 0.10 | 1.05 | Current smoker |
| 1.05 | 1.2 | 0.20 | 0.10 | 1.03 | Heavy former smoker |
| 3 |  |  |  |  |  |
| 1.40 | 2.8 | 0.18 | 0.10 | 1.25 | Low BMI |
| 1.25 | 2 | 0.18 | 0.10 | 1.16 | GOLD stage 4 FEV_1_ vs. others |
| 1.16 | 1.4 | 0.18 | 0.10 | 1.13 | 1 point more on dyspnoea scale |
| 1.13 | 1.2 | 0.18 | 0.10 | 1.11 | Current smoker |
| 1.11 | 1.2 | 0.18 | 0.10 | 1.09 | Heavy former smoker |

a In each set of scenarios, the apparent relative risk values that are indented represent partial adjustment by the factor in the row above.

References

1. Curkendall SM, DeLuise C, Jones JK, Lanes S, Stang MR, Goehring E, Jr., et al. Cardiovascular disease in patients with chronic obstructive pulmonary disease, Saskatchewan Canada cardiovascular disease in COPD patients. Ann Epidemiol. 2006 Jan;16(1):63-70.

2. Soriano JB, Maier WC, Visick G, Pride NB. Validation of general practitioner-diagnosed COPD in the UK General Practice Research Database. Eur J Epidemiol. 2001;17(12):1075-80.

3. GOLD. Global strategy for the diagnosis, management, and prevention of chronic obstructive pulmonary disease. 2016. Available at: <http://goldcopd.org/global-strategy-diagnosis-management-prevention-copd-2016/>. Accessed 10 June 2016.

4. Verhamme KM, Afonso AS, van Noord C, Haag MD, Koudstaal PJ, Brusselle GG, et al. Tiotropium Handihaler and the risk of cardio- or cerebrovascular events and mortality in patients with COPD. Pulm Pharmacol Ther. 2012 Feb;25(1):19-26.

5. Schneeweiss S. Sensitivity analysis and external adjustment for unmeasured confounders in epidemiologic database studies of therapeutics. Pharmacoepidemiol Drug Saf. 2006 May;15(5):291-303.

6. Celli BR, Cote CG, Marin JM, Casanova C, Montes de Oca M, Mendez RA, et al. The body-mass index, airflow obstruction, dyspnea, and exercise capacity index in chronic obstructive pulmonary disease. N Engl J Med. 2004 Mar 4;350(10):1005-12.

7. Calverley PM, Tetzlaff K, Dusser D, Wise RA, Mueller A, Metzdorf N, et al. Determinants of exacerbation risk in patients with COPD in the TIOSPIR study. Int J Chron Obstruct Pulmon Dis. 2017;12:3391-405.

8. Ramsey SD, Hobbs FD. Chronic obstructive pulmonary disease, risk factors, and outcome trials: comparisons with cardiovascular disease. Proc Am Thorac Soc. 2006 Sep;3(7):635-40.
